# Design, synthesis and application of a magnetic H-bond catalyst in the preparation of new nicotinonitriles *via* cooperative vinylogous anomeric-based oxidation†

**DOI:** 10.1039/d4ra01163e

**Published:** 2024-05-22

**Authors:** Mahdiyeh Navazeni, Mohammad Ali Zolfigol, Hossein Ahmadi, Hassan Sepehrmansourie, Ardeshir Khazaei, Mojtaba Hosseinifard

**Affiliations:** a Department of Organic Chemistry, Faculty of Chemistry and Petroleum Sciences, Bu-Ali Sina University Hamedan 6517838683 Iran zolfi@basu.ac.ir mzolfigol@yahoo.com Khazaei_1326@yahoo.com +98 8138380709 +98 8138282807; b Department of Energy, Materials and Energy Research Center P. O. Box 31787-316 Karaj 401602 Iran

## Abstract

Herein, we designed and synthesized a new H-bond magnetic catalyst with 2-tosyl-*N*-(3-(triethoxysilyl)propyl)hydrazine-1-carboxamide as a sensitive H-bond donor/acceptor. We created an organic structure with a urea moiety on the magnetic nanoparticles, which can function as a hydrogen bond catalyst. Hydrogen bond catalysts serve as multi-donor/-acceptor sites. Additionally, we utilized magnetic nanoparticles in the production of the target catalyst, giving it the ability to be recycled and easily separated from the reaction medium with an external magnet. We evaluated the catalytic application of Fe_3_O_4_@SiO_2_@tosyl-carboxamide as a new magnetic H-bond catalyst in the synthesis of new nicotinonitrile compounds through a multicomponent reaction under solvent-free and green conditions with high yields (50–73%). We confirmed the structure of Fe_3_O_4_@SiO_2_@tosyl-carboxamide using various techniques. In addition, the structures of the desired nicotinonitriles were confirmed using melting point, ^1^H-NMR, ^13^C-NMR and HR-mass spectrometry analysis. The final step of the reaction mechanism was preceded *via* cooperative vinylogous anomeric-based oxidation (CVABO).

## Abstract 2

Herein, we designed and synthesized a new H-bond magnetic catalyst with 2-tosyl-*N*-(3-(triethoxysilyl)propyl)hydrazine-1-carboxamide as a sensitive H-bond donor/acceptor in the synthesis of new nicotinonitrile compounds.

## Graphical abstract

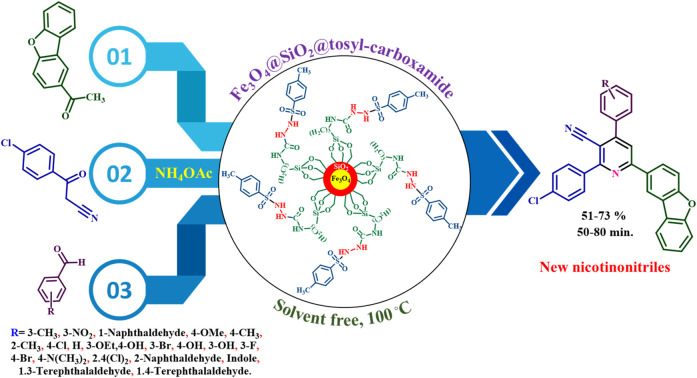

## Introduction

Magnetic nanoparticles (MNPs) have special properties. These particles are usually composed of magnetic materials, such as iron, titanium, cobalt, nickel and their corresponding alloys.^1,2^ The characteristic feature of magnetic nanoparticles is the ability to change their shape and size owing to their nanometer dimensions. By reducing the size of magnetic particles to nanometers, their surface area increases relative to their volume, and this increases the nonlinear magnetic behavior and their unique properties.^3–5^ On account of the remarkable properties of magnetic nanoparticles, they are used in numerous fields. For example, in medicine, these particles are used as imaging and therapeutic agents in the diagnosis and treatment of diseases. They also have other applications in various fields, such as electronics, catalysts, energy, environmental protection and nanoindustries.^6–9^ Magnetic nanoparticles can be modified for catalytic applications and offer suitable catalytic properties using different organic linkers. In recent years, various reports have been presented on the modification of MNPs to perform multicomponent reactions, oxidation, reduction, coupling and other organic reactions.^10–12^ One of the characteristics of catalysts used in different fields is proper catalytic performance, such as high selectivity, no side reactions and easy separation from the reaction medium with an external magnet.^13,14^

Hydrogen bond catalysts are substances that facilitate secondary bond formation between hydrogen and lone pairs within molecules.^15,16^ Hydrogen bonds occur when a hydrogen atom is attracted by a lone pair from an electronegative atom (such as oxygen, nitrogen, or fluorine) of another molecule. These bonds can significantly affect the physical and chemical properties of molecules and play an important role in various biological and chemical processes.^17^ While hydrogen bonding occurs naturally between certain molecules, catalysts can speed up the formation or strengthening of these bonds. Catalysts work by providing an environment or specific functional groups that promote hydrogen bond formation. They can also stabilize transition states during the reaction and reduce the activation energy required for the process.^18,19^ There are different types of hydrogen bonding catalysts, including organic compounds, metal complexes, enzymes, and even some solid materials.^20–22^

**Fig. 1:**
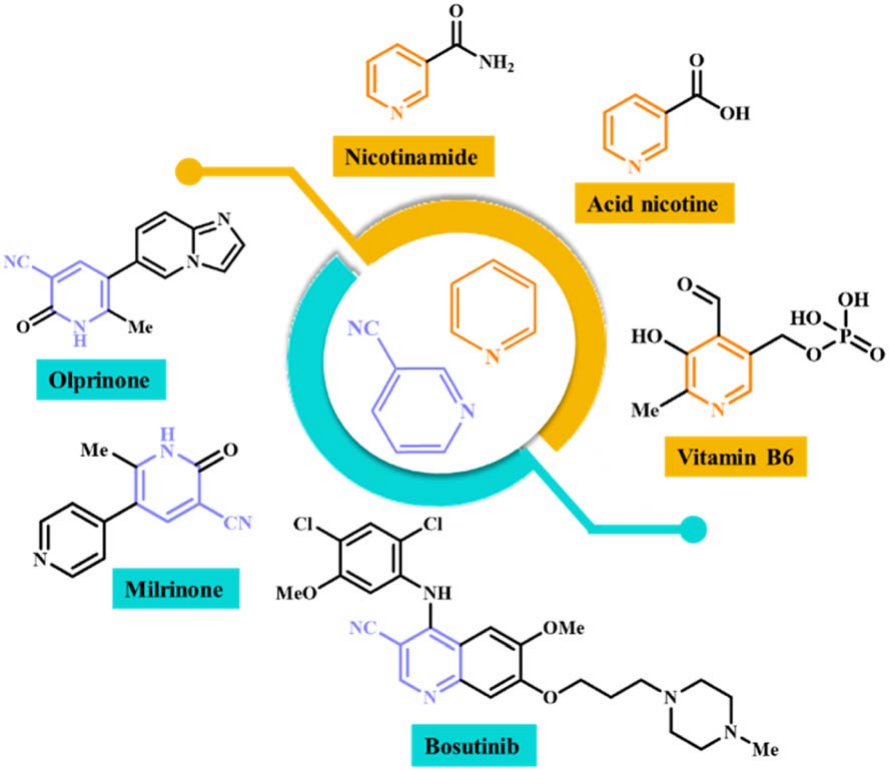
Structure of medicinal compounds containing nicotinonitrile and pyridine groups.

Nicotinonitriles are a class of organic compounds that contain a nitrile group (–CN) attached to the pyridine ring of nicotine.^23^ Various nicotinonitriles that have medicinal and biological properties have been introduced in recent years. These compounds have antibacterial, antifungal, anti-depressant, anti-aging, anti-inflammatory and anti-Alzheimer properties.^24–26^ Also, the compounds that have pyridine nuclei in their structure have shown good medicinal properties. Pyridine derivatives have shown anti-inflammatory properties and mediators involved in the inflammatory process.^27,28^ They can inhibit cancer cell proliferation, promote apoptosis (cell death) in tumor cells, or interfere with specific molecular targets involved in cancer development. Some pyridine compounds have cardiovascular effects, including vasodilation or inhibition of platelet aggregation, which may be beneficial in conditions such as hypertension or thrombotic disorders.^29,30^Fig. 1 shows a few structures with pyridine and nicotinonitrile cores, whose medicinal and biological properties have been proven.^31–33^

**Fig. 2:**
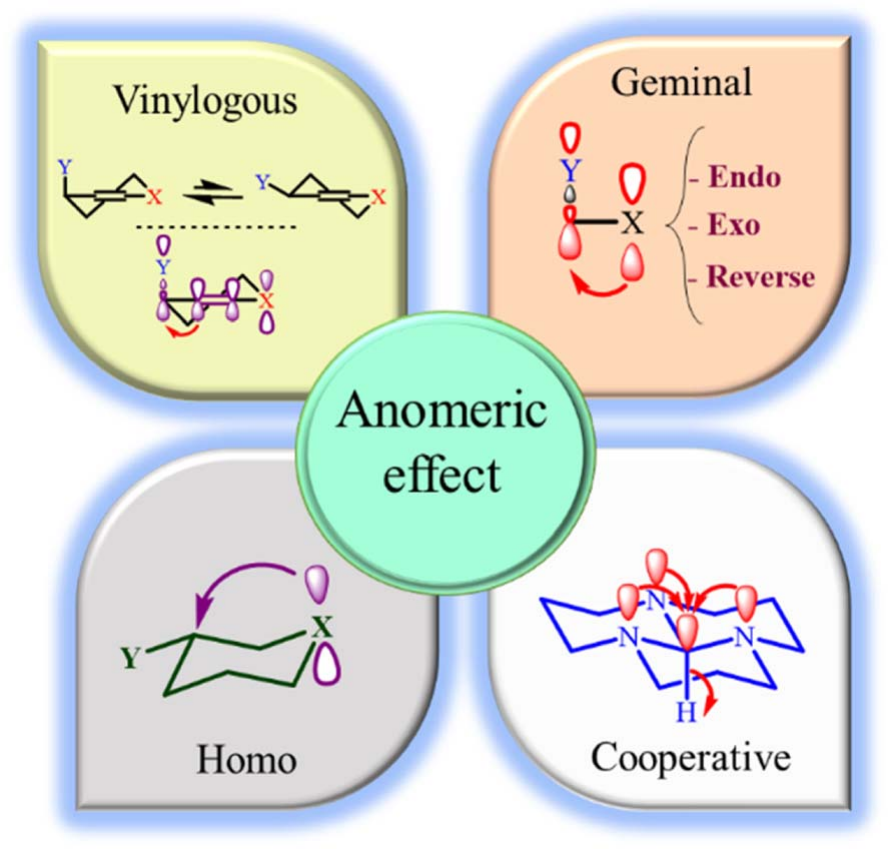
The endo, exo, homo, geminal and vinylogous anomeric effect.

The anomeric effect (AE) is the main driving force for the aromatization of pyridines. Recently, we have introduced anomeric-based oxidation (ABO) *via* the sharing of electrons of the heteroatom lone pair (X: N, O) at the tetrahedral carbon to the C–H antibonding orbital (nX σ*C–H). Also, the role of AE as a strong driving force in organic synthesis has been comprehensively reviewed by four international research groups, including us.^34,35^ Due to the importance of this concept in promoting the synthesis of organic compounds with biological properties, the development of cooperative vinylogous anomeric-based oxidation (CVABO) is our main interest. To the best of our knowledge, the anomeric effect has different subcategories, such as endo, exo, homo, geminal, inverse, network, and vinylogous (Fig. 2).^36–38^ Our research group has recently explored the application of AE to explain the activities of organic molecules.^39–43^

Expanding the design of heterogeneous catalysts is one of the main goals of this report. The importance of magnetic nanoparticles-based catalysts has prompted us to introduce Fe_3_O_4_@SiO_2_@tosyl-carboxamide as a new magnetic H-bond catalyst. This catalyst is based on magnetic Fe nanoparticles to create urea moieties on the magnetic nanoparticles. The presence of urea moieties on the structure of a magnetic catalyst enables it to give hydrogen bonds with donor/acceptor sites on the other molecules. The magnetic property of the presented catalyst helps the user to easily separate it from the reaction medium with an external magnet. The synthesized catalyst was used to prepare nicotinonitriles for studying its catalytic performance based on hydrogen bonding activity.

## Experimental section

### Preparation of 2-tosyl-*N*-(3-(triethoxysilyl)propyl)hydrazine-1-carboxamide as a new H-bond linker

To prepare the target ligand, 10 mmol (1.9 g) of *p*-toluenesulfonyl chloride was dissolved in 20 mL of THF, and placed in an ice bath. Then, 40 mmol (1.6 mL) of 80% hydrazine hydrate was gradually added. The reaction was stirred for 30 min in an ice bath. Then, the reaction mixture was warmed up to 25 °C and stirred for 2 h. After the completion of the reaction, the reaction mixture was decanted into a mixture of EtOAc and H_2_O (2 : 1). After that, the organic phase was separated and dried with sodium sulfate. The EtOAc solvent was then evaporated to give *p*-toluenesulfonyl hydrazide.^44^ In the next step, 5 mmol (0.93 g) of *p*-toluenesulfonyl hydrazide and 6 mmol (1.48 g) of triethoxy(3-isocyanatopropyl)silane were added in 10 mL of CH_2_Cl_2_. The mixture was vigorously stirred for 24 h at 40 °C until the reaction was completed. After this time, the solvent was evaporated and the obtained solid was washed with CH_2_Cl_2_. Finally, a white powder as a desired linker was obtained.

### Preparation of Fe_3_O_4_@SiO_2_@tosyl-carboxamide as a new magnetic H-bond catalyst

For the synthesis of the final catalyst, Fe_3_O_4_@SiO_2_ was synthesized according to the previous reports.^45^ Next, 1 g of Fe_3_O_4_@SiO_2_ was dispersed in 120 mL of toluene using ultrasonic radiation. Then, 2 mmol (0.87 g) of the combined linker was added to the mixture. Then, the obtained mixture was refluxed for 48 h. The resulting catalyst was washed several times with hot toluene. Chloroform was added to the precipitate to remove any unreacted linker. Finally, the obtained catalyst was separated using an external magnet and dried at 100 °C for 24 h.

### The general method for the preparation of nicotinonitrile derivatives

First, starting material 3-(4-chlorophenyl)-3-oxopropanenitrile and 1-(dibenzo[*b*,*d*]furan-2-yl)ethan-1-one were synthesized according to the previous report.^46^ For this synthesis, dibenzo[*b*,*d*]furan (5 mmol, 0.8 g), acetyl chloride (10 mmol, 0.78 g, 0.91 mL), AlCl_3_ (5 mmol, 0.65 g) and CH_2_Cl_2_ (30 mL) were added into the 50 mL bottom flask and stirred for 60 min at 25 °C. After the completion of the reaction, the reaction mixture was decanted to the mixture of EtOAc and H_2_O (1 : 1) solvents.^47^ Then, for the synthesis of nicotinonitrile derivatives, a mixture of 1-(dibenzo[*b*,*d*]furan-2-yl)ethanone (1 mmol, 0.21 g), 3-(4-chlorophenyl)-3-oxopropanenitrile (1 mmol, 0.179 g), ammonium acetate (1.5 mmol, 0.115 g), aromatic aldehyde (1 mmol) and 10 mg of Fe_3_O_4_@SiO_2_@tosyl-carboxamide as a new magnetic H-bond catalyst were stirred under solvent-free conditions at 100 °C. The progress of the reaction was monitored using TLC technique (*n*-hexane and ethyl acetate). When the reaction was completed, hot acetone (15 mL) was poured into the reaction mixture. The catalyst was separated from the reaction mixture with an external magnet. Then, the acetone solvent was evaporated. The remaining solid was washed several times with hot ethanol to obtain the pure product. The structures of the new nicotinonitriles were evaluated and confirmed using melting point, ^1^H-NMR, ^13^C-NMR and HR-Mass techniques (spectral specifications are available in ESI†).

## Results and discussion

### Catalyst preparation strategy

**Fig. 3:**
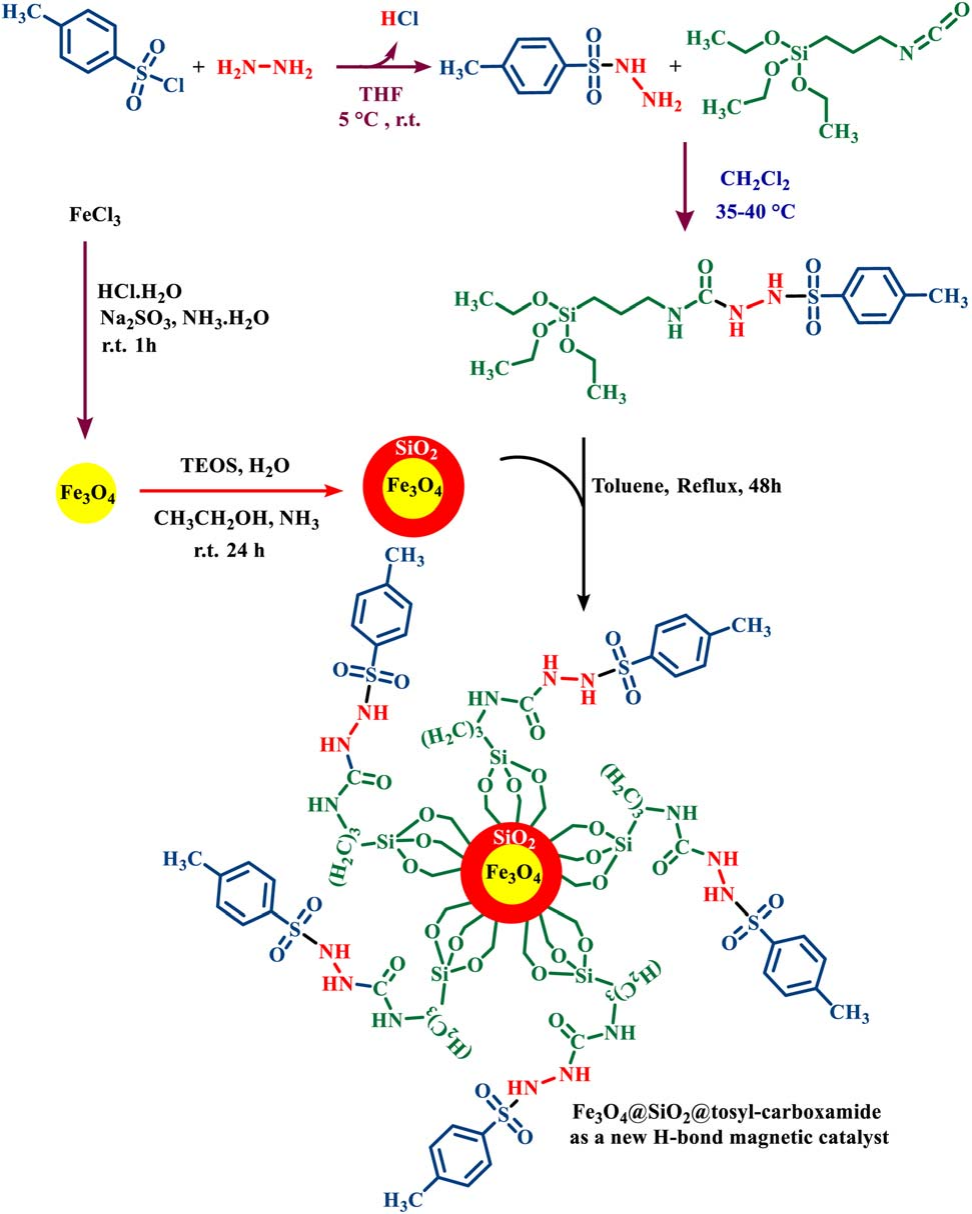
Catalyst preparation strategy for the synthesis of Fe_3_O_4_@SiO_2_@tosyl-carboxamide as a new magnetic H-bond catalyst with multi-donor/-acceptor linkers.

The rational design of catalysts on the basis of hydrogen bonding has received much attention due to their ability to selectively carry out organic reactions.^15,18^ In this study, a magnetic catalyst based on its hydrogen bonding activity has been designed and synthesized. First, magnetic iron nanoparticles (Fe_3_O_4_) were synthesized. The primary catalyst bed was designed and synthesized (Fe_3_O_4_@SiO_2_) by covering SiO_2_ on the Fe_3_O_4_. On the other hand, by using *p*-toluenesulfonyl chloride, hydrazine and triethoxy(3-isocyanatopropyl)silane, a ligand with a urea moiety was synthesized. The abovementioned ligand was reacted with the magnetic nanoparticles to produce Fe_3_O_4_@SiO_2_@tosyl-carboxamide as a new magnetic H-bond catalyst under refluxing toluene (Fig. 3). The catalyst designed in this research works based on the hydrogen bonding interaction. The presence of the urea moieties in the catalyst structure creates the connecting part of the H-bond catalysis. The NH groups in urea moieties can activate various functional groups in the structure of raw materials because the presence of carbonyl adjacent to the NH group is susceptible to donor/acceptor H-bond catalysis. This capability of urea groups has been used to create the target catalyst. The structure of the desired catalyst was approved using various techniques, such as FT-IR, XRD, SEM, EDS, elemental mapping, VSM, TGA and DTG.

**Fig. 4:**
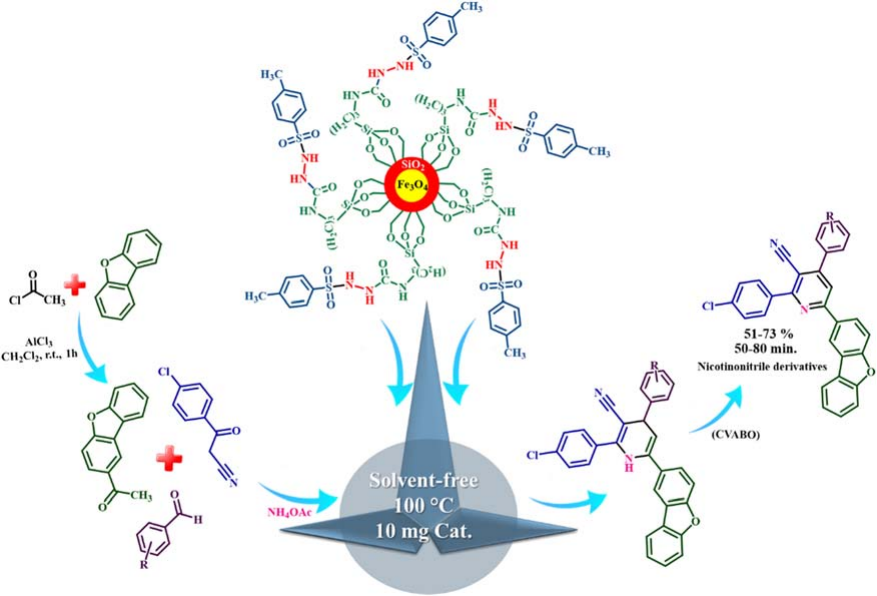
Catalytic application strategy for the preparation of nicotinonitrile derivatives using Fe_3_O_4_@SiO_2_@tosyl-carboxamide as a new magnetic H-bond catalyst.

The presence of pyridine and nicotinonitrile derivatives in biological derivatives is very important and they show good medicinal properties.^25^ In line with the development of these materials, after the design and synthesis of the target catalyst, its catalytic efficiency was evaluated in the synthesis of a wide range of new nicotinonitriles. Fe_3_O_4_@SiO_2_@tosyl-carboxamide showed that it can catalyze the synthesis of target compounds with high efficiency and a relatively suitable reaction time (Fig. 4). The structure of new nicotinonitriles was confirmed using melting point, ^1^H-NMR, ^13^C-NMR and HR-Mass techniques (spectral specifications are available in ESI†).

The structure of new nicotinonitriles was confirmed using melting point, ^1^H-NMR, ^13^C-NMR and HR-Mass techniques (spectral specifications are available in ESI†). The mechanism of the reaction was also investigated. For this purpose, it was observed that the aromatization of the pyridine ring is done through a cooperative vinylogous anomeric based oxidation (CVABO).^36^

**Fig. 5:**
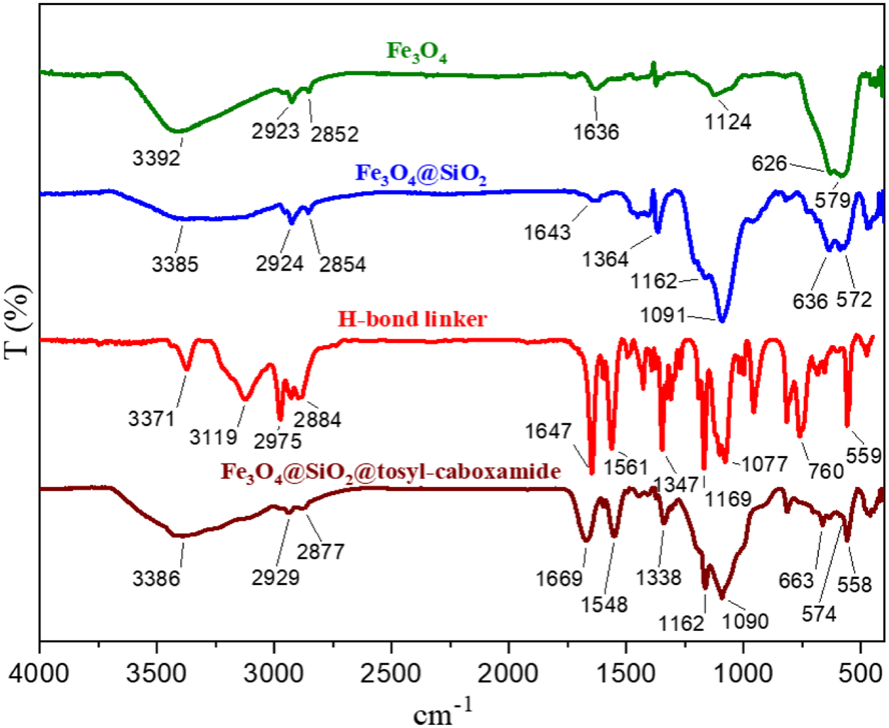
FT-IR spectra of different stages of the synthesis of the catalyst.

FT-IR technique was used to check the functional groups of different stages of catalyst synthesis (Fig. 5). In the FT-IR spectrum of Fe_3_O_4_ magnetite nanoparticles, two sharp peaks appeared in the region of 579 cm^−1^ and 626 cm^−1^, attributed to the stretching vibration of Fe–O bonds. The broad band appearing at 3392 cm^−1^ is related to the stretching vibration of the O–H bond. The FT-IR spectrum of the Fe_3_O_4_@SiO_2_ core–shell magnetic nanoparticles is also shown in this figure. The strong peak at the region of 1091 cm^−1^ is related to the asymmetric stretching vibration of the Si–O–Si bond, and the appearing peak at the region of 813 cm^−1^ is related to the symmetric stretching vibration of this bond. The FT-IR pattern of the synthesized linker is also shown in this figure. A comparison of the FT-IR patterns for the synthesized catalyst, Fe_3_O_4_@SiO_2_ magnetic substrate, as well as a linker, is shown in Fig. 5. All of the peaks found in different stages of the catalyst synthesis, as well as the corresponding linker, appear in the FT-IR pattern of the final catalyst, demonstrating the correct synthesis and structure of the catalyst.

**Fig. 6:**
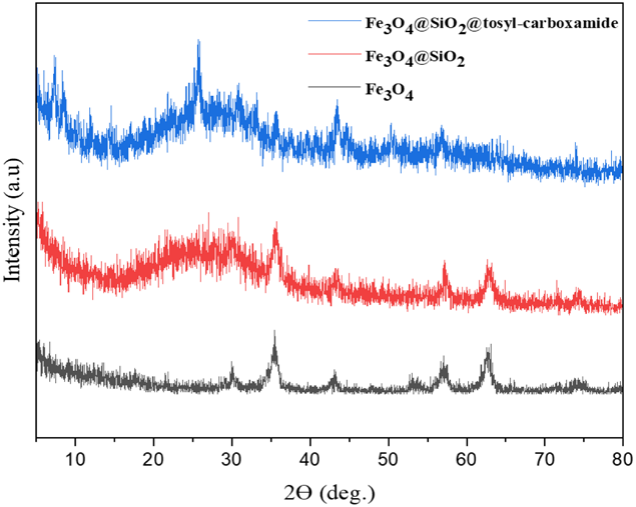
Comparison XRD pattern of Fe_3_O_4_, Fe_3_O_4_@SiO_2_ and Fe_3_O_4_@SiO_2_@tosyl-carboxamide as a new magnetic H-bond catalyst.

The proof of the pattern of crystal plates of Fe_3_O_4_, Fe_3_O_4_@SiO_2_ and Fe_3_O_4_@SiO_2_@tosyl-carboxamide as a new magnetic H-bond catalyst is shown in Fig. 6 in a comparative way.

**Fig. 7:**
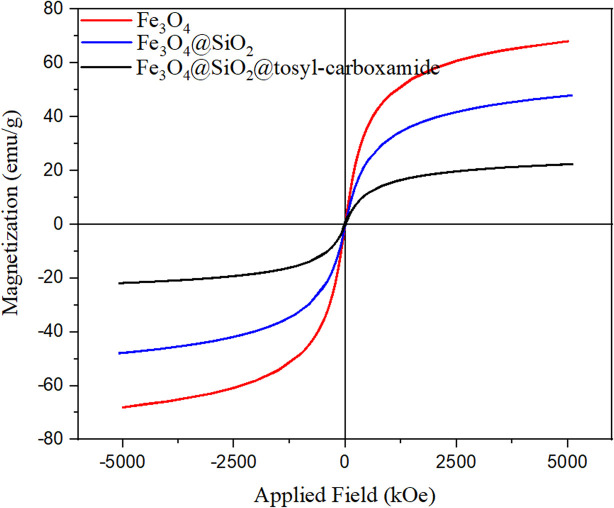
Magnetization curves of Fe_3_O_4_@SiO_2_@tosyl-carboxamide as a H-bond catalyst.

The crystal pattern of Fe_3_O_4_ agrees well with previous reports.^39^ Accordingly, the peaks of the areas in 2*θ* = 18.23°, 30.35°, 35.43°, 43.72°, 53.69°, 57.36°, 62.91° and 74.58° correspond to the Fe_3_O_4_ diffraction lines (111), (220), (311), (400), (422), (511), (440) and (533), respectively. The wide peak of the 2*θ* = 20–30°, range is related to SiO_2_, which is well placed on Fe_3_O_4_. In the crystal model of the final catalyst, the peaks corresponding to Fe_3_O_4_ and SiO_2_ are visible, and the new peaks are probably related to the added linker, which indicates the correct synthesis of the catalyst. Also, to confirm the formation of the synthesized catalyst, the obtained VSM analysis results of all three stages were checked (Fig. 7).

The obtained results of this analysis for the Fe_3_O_4_ magnetic nanoparticles showed a high magnetic property with a saturation magnetization of 68 emu g^−1^. By placing a SiO_2_ coating on Fe_3_O_4_, as expected, a decrease in saturation magnetism was observed (48 emu g^−1^). The VSM analysis of the catalyst showed a lower magnetic property than the initial substrate (22 emu g^−1^), which is a confirmation of the linker immobilization on the surface of the magnetic substrate.

**Fig. 8:**
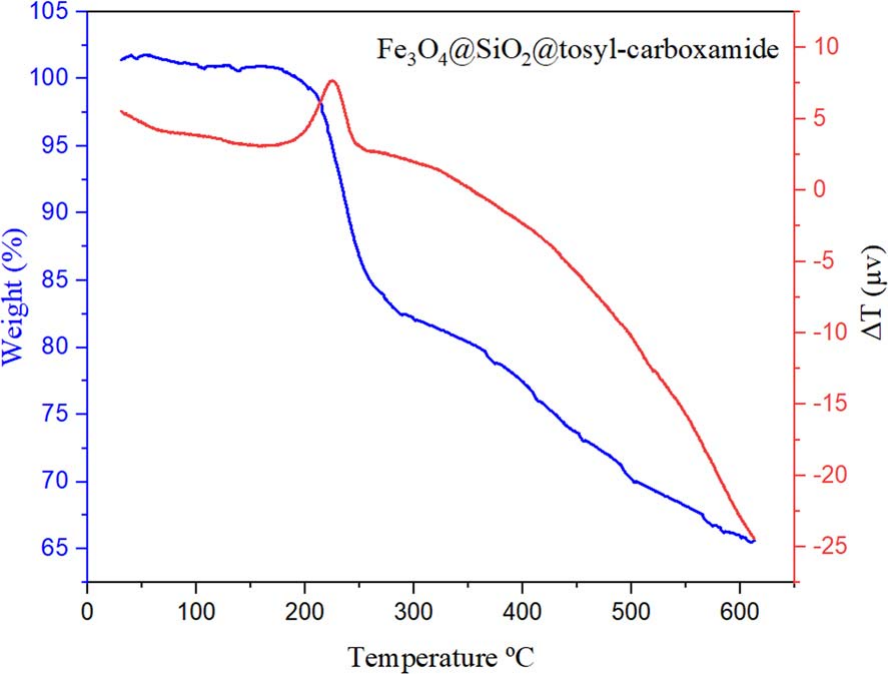
TG & DTA analysis of Fe_3_O_4_@SiO_2_@tosyl-carboxamide as a new magnetic H-bond catalyst.

TGA & DTA techniques also were used to prove the thermal stability of the final synthesized catalyst. According to the obtained results, this catalyst can be used up to a temperature of 230 °C without its structure collapsing (Fig. 8).

**Fig. 9:**
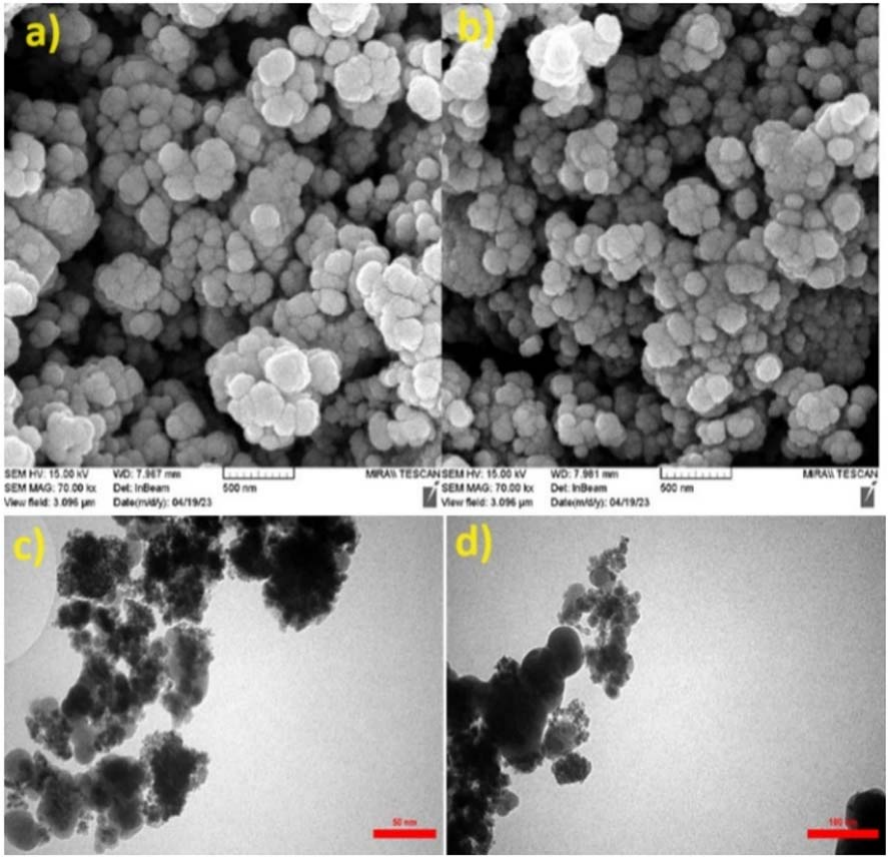
Scanning electron microscopy (SEM) (a and b) and transmission electron microscopy (TEM) (c and d) images of Fe_3_O_4_@SiO_2_@tosyl-carboxamide as a new magnetic H-bond catalyst.

SEM analysis was performed to detect the surface morphology of the synthesized catalyst. The SEM micrographs of Fe_3_O_4_@SiO_2_@tosyl-carboxamide as a catalyst showed that the particles were not aggregated, and the cauliflower morphology was established for this structure (Fig. 9a and b). The TEM images were recorded with further focusing, and it clearly shows that they are made from nanoparticles and possess a core–shell structure. The TEM results confirmed the obtained data from the SEM images (Fig. 9c and d).

### Catalytic performance

To expand the range of nicotinonitrile derivatives, the reaction conditions were optimized. In the optimization conditions, the reaction between 1-(dibenzo[*b*,*d*]furan-2-yl)ethenone (1 mmol, 0.21 g), 3-(4-chlorophenyl)-3-oxopropanenitrile (1 mmol, 0.179 g), 4-chlorobenzaldehyde (1 mmol, 0.14 g), and ammonium acetate (1.5 mmol, 0.115 g) was selected as a model reaction. The model reaction was investigated in different conditions, such as temperature, amount of catalyst and use/non-use of solvent. Table 1 shows the results of this review. Initially, the model reaction was evaluated at different temperatures under solvent-free conditions with 10 mg of catalyst (entries 1–6). The results showed that the optimal temperature for the highest product yield and the shortest reaction time is 100 °C. Then, the model reaction was tested at this optimal temperature with different amounts of catalyst (entries 7–9). Based on these obtained results, it was found that a suitable result is obtained from only using 10 mg of catalyst. Next, the model reaction with different solvents was evaluated, and the obtained results showed that there is no improvement in the yield and reaction time of the products (entries 10–19). Therefore, the optimum conditions for the synthesis of the target derivative were obtained in solvent-free conditions, at 100 °C, and in the presence of 10 mg of catalyst (entry 4).

**Table 1:** Optimizing the conditions for the synthesis of nicotinonitrile derivatives^a^

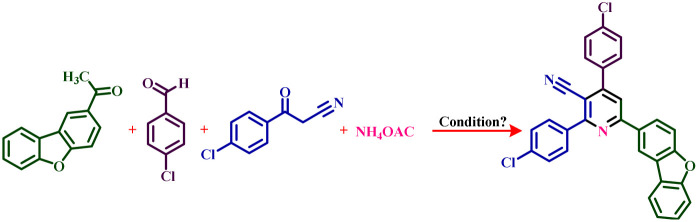					
Entry	Solvent	Amount of catalyst (mg)	Temp. (°C)	Time (min)	Yield (%)
1^a^	—	10	25	120	Trace
2^a^	—	10	50	100	61
3^a^	—	10	80	50	68
**4** ^**a**^	**—**	**10**	**100**	**50**	**73**
5^a^	—	10	110	50	72
6^a^	—	10	120	50	69
7^a^	—	5	100	50	70
8^a^	—	15	100	50	72
9^a^	—	—	100	70	64
10^b^	EtOH	10	Reflux	240	61
11^b^	MeOH	10	Reflux	240	62
12^b^	H_2_O	10	Reflux	300	Trace
13^b^	CH_3_CN	10	Reflux	260	59
14^b^	DMF	10	Reflux	270	73
15^b^	Acetone	10	Reflux	300	Trace
16^b^	EtOAc	10	Reflux	300	Trace
17^b^	*n*-Hexane	10	Reflux	300	Trace
18^b^	CHCl_3_	10	Reflux	300	Trace
19^b^	CH_2_Cl_2_	10	Reflux	300	Trace
20^c^	—	50	100	300	55

a The reaction condition: ^a,b^1-(dibenzo[*b*,*d*]furan-2-yl)ethenone (1 mmol, 0.21 g), 3-(4-chlorophenyl)-3-oxopropanenitrile (1 mmol, 0.179 g), 4-chlorobenzaldehyde (1 mmol, 0.14 g), and ammonium acetate (1.5 mmol, 0.115 g) with different amounts of catalyst and solvent. ^c^(Large scale): 1-(dibenzo[*b*,*d*]furan-2-yl)ethenone (5 mmol, 1.05 g), 3-(4-chlorophenyl)-3-oxopropanenitrile (5 mmol, 0.895 g), 4-chlorobenzaldehyde (5 mmol, 0.7 g), and ammonium acetate (8 mmol, 0.616 g) and 50 mg catalyst were stirred under solvent-free conditions at 100 °C to obtain the H-7 derivative (1.354 g, 55%).

The expansion of synthesized compounds is another goal of this report. For this purpose, after determining the optimal reaction conditions, different aromatic aldehydes with donor and electron acceptor substitutions were used in ideal conditions to prepare a wide range of nicotinonitriles. The results are shown in Table 2. According to the obtained results, products with high efficiency and short reaction time have been obtained, which indicates the proper performance of Fe_3_O_4_@SiO_2_@tosyl-carboxamide as a new magnetic H-bond catalyst.

**Table 2:** Catalytic synthesis of nicotinonitrile derivatives in the presence of Fe_3_O_4_@SiO_2_@tosyl-carboxamide as a new magnetic H-bond catalyst

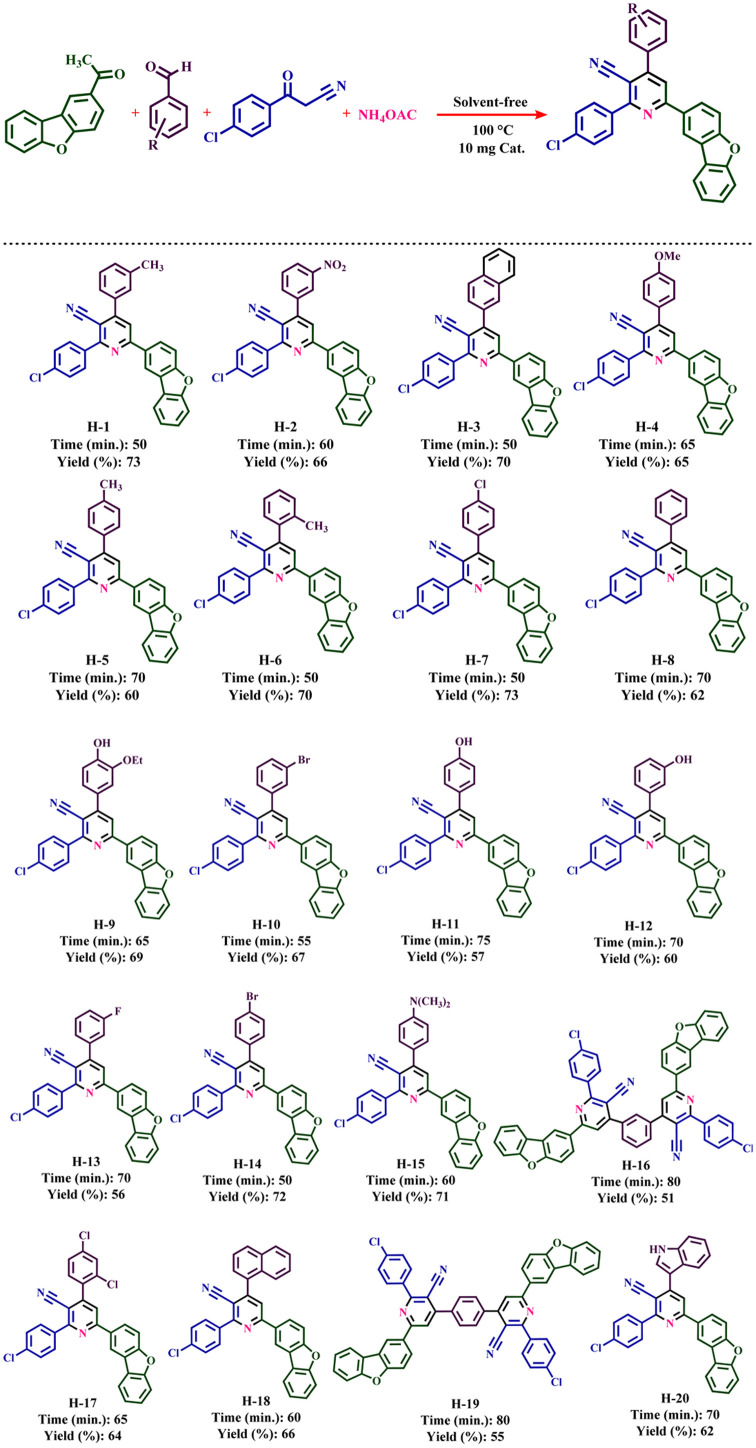

**Fig. 10:**
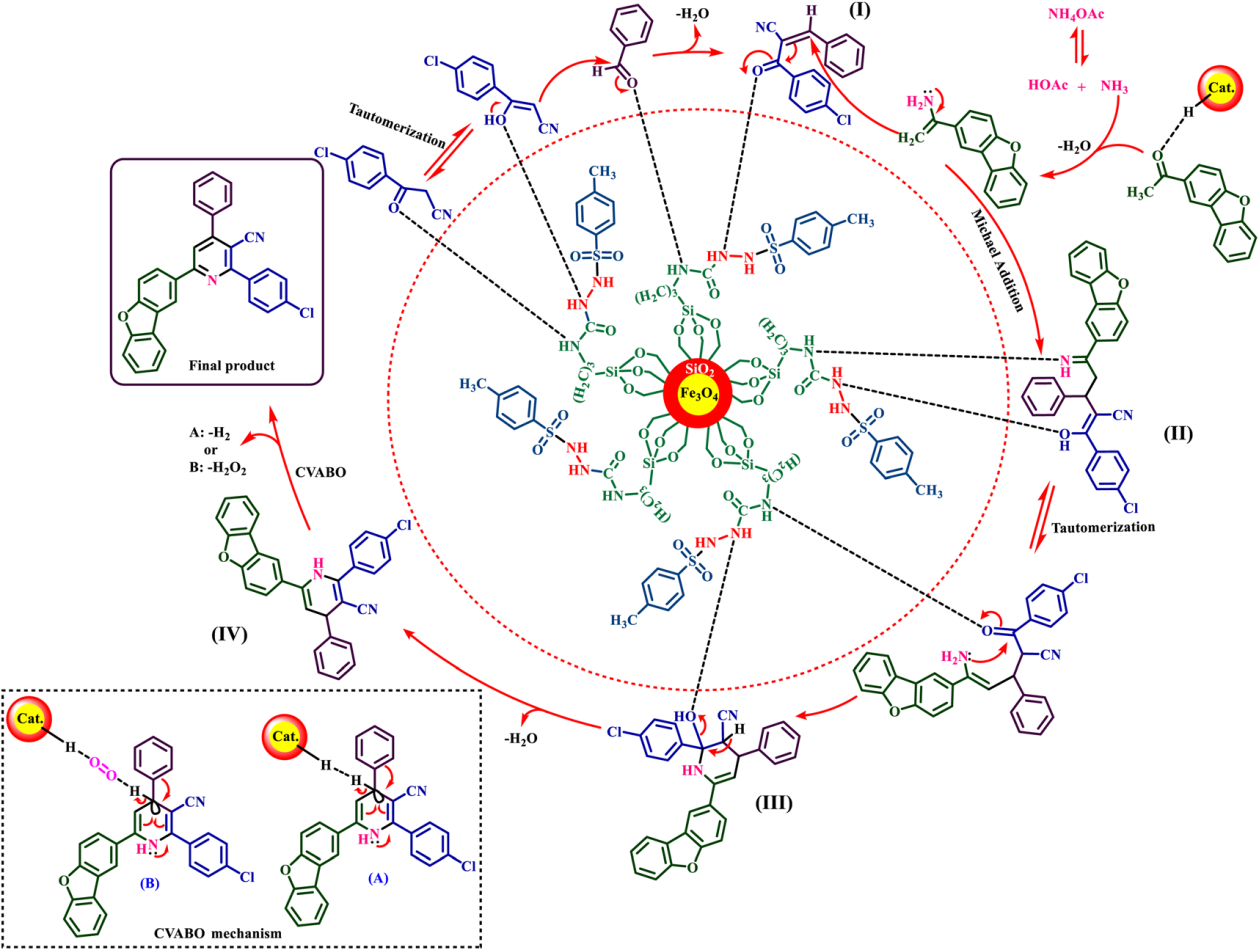
Proposed mechanism for the synthesis of nicotinonitrile derivatives using Fe_3_O_4_@SiO_2_@tosyl-carboxamide as a new magnetic H-bond catalyst.

In the proposed mechanism, the 3-(4-chlorophenyl)-3-oxopropanenitrile compound is first converted into the enol form by the interaction with the H-bond donor/acceptor sites of the presented catalyst and reacts with the aldehyde. After releasing one molecule of H_2_O, intermediate (I) is produced. On the other hand, ammonium obtained from ammonium acetate reacts with 1-(dibenzo[*b*,*d*]furan-2-yl)ethenone and intermediate (I) is produced, which acts as a Michael acceptor, and intermediate (II) is created. Intermediate (II) undergoes tautomerization and intramolecular cyclization is converted to intermediate (III). Intermediate (III) is converted to intermediate (IV) by removing another H_2_O molecule. Finally, 1,4-dihydropyridines (IV) are converted to the corresponding pyridine derivatives *via* the CVABO mechanism, liberating a molecule of hydrogen (H_2_) and/or hydrogen peroxide (H_2_O_2_)^36,48^ (Fig. 10).

In another study to prove the results obtained from this report, the model reaction was evaluated using other reported organic and inorganic catalysts, as well as the precursors of the initial stages of the presented catalyst. The results are shown in Table 3. According to the obtained results, Fe_3_O_4_@SiO_2_@tosyl-carboxamide as a new magnetic H-bond catalyst provides better efficiency and reaction time than other used catalysts.

**Table 3:** Evaluation of various catalysts for the synthesis of nicotinonitrile derivatives

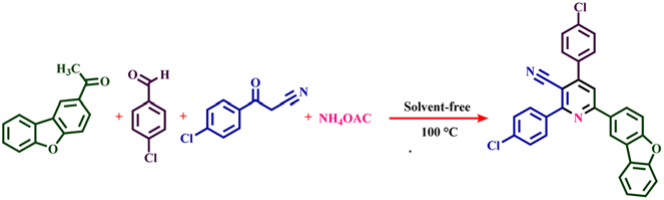				
Entry	Catalyst	Amount of catalyst	Time (min)	Yield (%)
1	SSA^49^	10 (mg)	220	30
2	NaOH	10 (mol%)	220	43
3	N(Et)_3_	10 (mol%)	120	42
4	*p*-TSA	10 (mol%)	240	37
5	K_2_CO_3_	10 (mol%)	120	Trace
6	KOH	10 (mol%)	120	52
7	Piperidine	10 (mol%)	120	61
8	H_2_SO_4_	10 (mol%)	120	Trace
9	Poly(acetic acid)	10 (mg)	120	42
10	CQDs–N(CH_2_PO_3_H_2_)_2_ (ref. 50)	10 (mg)	100	38
11	Zr-UiO-66-PDC(CH_2_)_4_-SO_3_ (ref. 41)	10 (mg)	120	65
12	UiO-66-NH_2_/melamine/[N(CH_2_PO_3_H_2_)_2_]_2_ (ref. 51)	10 (mg)	210	46
13	UiO-66-NH_2_/TCT/2-amino-pyridine@Cu(OAc)_2_ (ref. 38)	10 (mg)	120	54
14	Fe_3_O_4_	10 (mol%)	240	25
15	Triethoxysilyl propyl tosylhydrazine-1-carboxamide	10 (mg)	120	42
**16**	**Fe**_**3**_ **O**_**4**_ **@SiO**_**2**_ **@tosyl-carboxamide (this work)**	**10 (mg)**	**50**	**73**

### Catalyst recovery

**Fig. 11:**
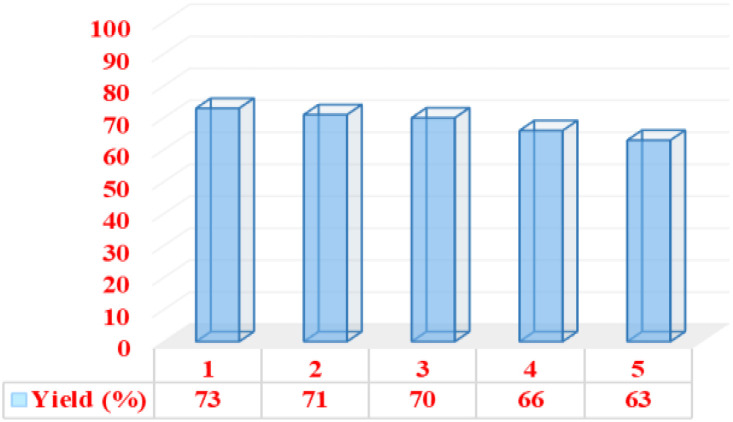
Recyclability of the catalyst in the synthesis of nicotinonitrile derivatives.

**Fig. 12:**
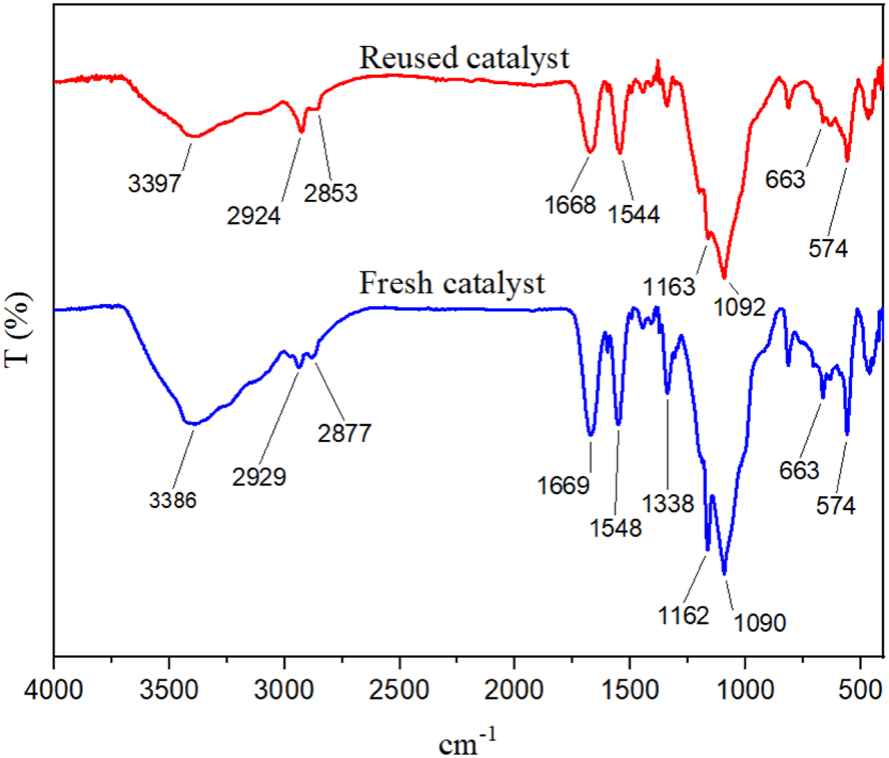
FT-IR spectra of the fresh and reused catalysts.

Catalyst recovery capability has several advantages, including environmental and economic benefits that add value to a designed task-specific catalyst. In this report, the recyclability of the catalyst under optimal conditions was investigated. The reaction mixture was dissolved in hot acetone after each step, and the catalyst was separated using an external magnet. After drying, the catalyst was prepared for the next reaction run. Our findings show that the catalyst can be recycled up to four times without a significant decrease in its catalytic activity (Fig. 11). FT-IR spectroscopy was used to prove the stability of the recovered catalyst. Fig. 12 shows the spectrum of the recovered catalyst, as well as the fresh catalyst. According to this analysis, the patterns of both spectra are almost similar, which shows the stability of the desired catalyst after being reused.

## Hot filtration test

To verify the catalytic activity of Fe_3_O_4_@SiO_2_@tosyl-carboxamide in producing nicotinonitrile derivatives, a hot filtration test was conducted using the model substrate under optimal reaction conditions. After 15 minutes of the reaction, it was stopped and the yield was determined to be 20%. In a separate reaction, the model reaction was repeated and at 15 minutes, hot acetone solvent was added to the reaction mixture. The catalyst was then separated from the system using an external magnet. Next, the solvent was evaporated and the reaction mixture was further stirred for another 1 h at the same optimum conditions. There was no noticeable improvement in the reaction yield. An ICP-OES test was also carried out for further investigation, revealing that 0.0055 × 10^−6^ mol per g Fe remained in the product. Therefore, it appeared that the reaction progressed with little efficiency after the catalyst was removed.

## Conclusion

In summary, we designed a magnetic catalyst modified with 2-tosyl-*N*-(3-(triethoxysilyl)propyl)hydrazine-1-carboxamide as an organic linker. By using and designing this method, urea groups were created on the nanoparticle structure, which turned it into a targeted catalyst with hydrogen bonding ability. The presented catalyst has recycle and reuse ability. It can be easily separated from the reaction medium with an external magnet. The structure of the target catalyst and synthesized new nicotinonitriles were confirmed using various techniques. The major advantages of the presented methodology are the recycling and reusing of the reported catalyst, high yield of products, and solvent-free and green conditions.

## Data availability

The datasets used and/or analyzed during the current study are available from the corresponding author on reasonable request.

## Author contributions

M. N., H. S.; and H. A.; methodology, validation, investigation. M. A. Z.; supervision, resources, project administration, funding acquisition, conceptualization, writing-review. A. K. supervision. M. H. Identification of synthesized products.

## Conflicts of interest

The authors declare no competing interests.

## Supplementary Material

RA-014-D4RA01163E-s001
