# Supplementary material for: Design, synthesis and application of a magnetic H-bond catalyst in the preparation of new nicotinonitriles via cooperative vinylogous anomeric-based oxidation
Source: RSC Adv. 2024 May 22;14(23):16607–16. doi: 10.1039/d4ra01163e (PMC11110150; doi:10.1039/d4ra01163e)
Supplement: RA-014-D4RA01163E-s001 [file RA-014-D4RA01163E-s001.pdf]

Electronic Supplementary Material (ESI) for RSC Advances.
This journal is © The Royal Society of Chemistry 2024

Design, synthesis and application of a magnetic H-bond catalyst in preparation of new nicotinonitriles *via* a cooperative vinylogous anomeric-based oxidation

Mahdiyeh Navazeni,[a] Mohammad Ali Zolfigol,*[a] Hossein Ahmadi,[a] Hassan Sepehrmansourie,[a] Ardeshir Khazaei,*[a] and Mojtaba Hosseinifard [b]

[a] Department of Organic Chemistry, Faculty of Chemistry and Petroleum Sciences, Bu-Ali Sina University, Hamedan 6517838683, Tel: +988138282807, Fax: +988138380709 Iran. E-mail: zolfi@basu.ac.ir & mzolfigol@yahoo.com (M. A. Zolfigol) or Khazaei_1326@yahoo.com (A. Khazaei).

[b] Department of Energy, Materials and Energy Research Center, P.O. Box 31787-316, Karaj, 401602, Iran.

Spectral data for raw materials and nicotinonitrile derivatives

2-Tosyl-N-(3-(triethoxysilyl)propyl)hydrazine-1-carboxamide

White solid; M.P.: 172-174 ˚C; R_f: 0.35; (*n*-Hexane: Ethyl acetate 3:7); FT-IR (KBr, cm^{-1}): 3373, 3124, 2975, 2897, 1646, 1562, 1347, 1168; ^1H NMR (300 MHz, DMSO-*d6*) δ_{ppm} 9.35 (s, 1H), 7.85 (s, 1H), 7.67 (d, *J* = 8.3 Hz, 2H), 7.36 (d, *J* = 8.4 Hz, 2H), 6.31 (t, *J* = 5.9 Hz, 1H), 3.72 (q, *J* = 7.0 Hz, 6H), 2.88 (q, *J* = 6.9 Hz, 2H), 2.38 (s, 3H), 1.33 (p, *J* = 7.4 Hz, 2H), 1.13 (t, *J* = 7.0 Hz, 9H), 0.48 – 0.41 (m, 2H). ^{13}C NMR (101 MHz, DMSO-*d6*) δ_{ppm} 157.5, 143.3, 135.3, 129.4, 127.7, 59.4, 57.6, 41.8, 23.2, 21.0, 18.1, 14.6, 8.2.

1-(Dibenzo[b,d]furan-2-yl)ethan-1-one

Cream solid; M.P.: 72-74 ˚C; R_f: 0.65; (*n*-Hexane: Ethyl acetate 7:3); FT-IR (KBr, cm^{-1}): 3070, 3004, 1676, 1599, 1250; ^1H NMR (400 MHz, DMSO-*d6*) δ_{ppm} 8.86 (d, *J* = 1.7 Hz, 1H), 8.31 (d, *J* = 7.8 Hz, 1H), 8.17 (s, 1H), 7.83 (d, *J* = 8.7 Hz, 1H), 7.71 (d, *J* = 8.3 Hz, 1H), 7.54 (t, *J* = 7.1 Hz, 1H), 7.42 (t, *J* = 7.0 Hz, 1H), 2.71 (s, 3H). ^{13}C NMR (101 MHz, DMSO-*d6*) δ_{ppm} 197.1, 158.0, 156.1, 128.3, 128.0, 127.6, 123.7, 123.1, 122.4, 121.7, 121.1, 111.9, 111.6, 26.9.

2-(4-Chlorophenyl)-6-(dibenzo[b,d]furan-2-yl)-4-(m-tolyl)nicotinonitrile (H-1)

Cream solid; M.P.: 245-247 ˚C; R_f: 0.48; (*n*-Hexane: Ethyl acetate 8:2); FT-IR (KBr, cm^{-1}): 3050, 2219, 1708, 1578, 1530, 1198; ^1H NMR (400 MHz, DMSO-*d6*) δ_{ppm} 9.13 (d, *J* = 2.0 Hz, 1H), 8.53 (dd, *J* = 8.7, 2.0 Hz, 1H), 8.33 (s, 1H), 8.27 (d, *J* = 7.1 Hz, 1H), 8.09 (d, *J* = 8.5 Hz, 2H), 7.86 (d, *J* = 8.9 Hz, 1H), 7.75 (d, *J* = 8.2 Hz, 1H), 7.70 (d, *J* = 8.5 Hz, 2H), 7.68 – 7.65 (m, 2H), 7.58 (t, *J* = 6.8 Hz, 1H), 7.53 (t, *J* = 7.9 Hz, 1H), 7.47 – 7.42 (m, 2H), 2.46 (s, 3H). ^{13}C NMR (101 MHz, DMSO-*d6*) δ_{ppm} 160.3, 158.2, 156.9, 156.1, 154.9, 138.2, 136.7, 136.3, 135.0, 132.1, 131.2, 130.5, 129.5, 128.5, 128.1, 127.5, 126.1, 124.3, 123.5, 121.6, 120.9, 118.9, 117.6, 112.1, 111.9, 104.0, 21.0. HR-MS m/z (%); Calc. for $C_{31}H_{19}ClN_2O$: 470.1186, found: 471.1274.

2-(4-Chlorophenyl)-6-(dibenzo[b,d]furan-2-yl)-4-(3-nitrophenyl)nicotinonitrile (H-2)

Brwon solid; M.P.: 296-298 ˚C; R_f: 0.44; (*n*-Hexane: Ethyl acetate 7:3); FT-IR (KBr, cm^{-1}): 3080, 2219, 1708, 1581, 1524, 1356, 1201; ^1H NMR (400 MHz, DMSO-*d6*) δ_{ppm} 9.15 (d, *J* = 1.9 Hz,

1H), 8.78 (s, 1H), 8.57 (dd, J = 8.7, 2.0 Hz, 1H), 8.51 – 8.47 (m, 2H), 8.36 (d, J = 8.2 Hz, 1H), 8.27 (d, J = 7.3 Hz, 1H), 8.13 (d, J = 8.5 Hz, 2H), 7.97 (t, J = 8.1 Hz, 1H), 7.89 (d, J = 8.9 Hz, 1H), 7.77 (d, J = 8.3 Hz, 1H), 7.73 (d, J = 8.5 Hz, 2H), 7.59 (t, J = 7.2 Hz, 1H), 7.47 (t, J = 7.5 Hz, 1H). ^{13}C NMR (101 MHz, DMSO-d_6) δ_{ppm} 169.9, 156.1, 147.9, 135.8, 132.0, 131.2, 130.5, 128.6, 128.2, 127.6, 124.7, 124.0, 123.5, 123.3, 121.5, 121.5, 121.0, 119.3, 111.9. HR-MS m/z (%); Calc. for $C_{30}H_{16}ClN_3O_3$: 501.0880, found: 502.0977.

2-(4-chlorophenyl)-6-(dibenzo[b,d]furan-2-yl)-4-(naphthalen-2-yl)nicotinonitrile (H-3)

Cream solid; M.P.: 257-259 ˚C; R_f: 0.5; *(n*-Hexane: Ethyl acetate 8:2); FT-IR (KBr, cm^{-1}): 3048, 2217, 1579, 1530, 1197; ^{1}H NMR (400 MHz, DMSO-d_6) δ_{ppm} 9.18 (d, J = 2.0 Hz, 1H), 8.58 (dd, J = 8.7, 1.9 Hz, 1H), 8.50 (s, 1H), 8.47 (s, 1H), 8.28 (d, J = 7.8 Hz, 1H), 8.19 (d, J = 8.6 Hz, 1H), 8.14 (d, J = 8.6 Hz, 2H), 8.10 – 8.06 (m, 2H), 8.00 (dd, J = 8.5, 1.9 Hz, 1H), 7.89 (d, J = 8.7 Hz, 1H), 7.76 (d, J = 8.2 Hz, 1H), 7.72 (d, J = 8.6 Hz, 2H), 7.70 – 7.66 (m, 2H), 7.58 (t, J = 7.1 Hz, 1H), 7.46 (t, J = 7.5 Hz, 1H). ^{13}C NMR (101 MHz, DMSO-d_6) δ_{ppm} 160.3, 158.2, 157.0, 156.1, 154.8, 136.7, 135.1, 133.8, 133.2, 132.5, 132.2, 131.2, 128.9, 128.6, 128.4, 128.1, 127.7, 127.5, 127.0, 126.2, 124.3, 123.5, 123.4, 121.5, 120.9, 119.3, 117.8, 112.2, 111.9, 104.2. HR-MS m/z (%); Calc. for $C_{34}H_{19}ClN_2O$: 506.1186, found: 507.1265.

2-(4-chlorophenyl)-6-(dibenzo[b,d]furan-2-yl)-4-(4-methoxyphenyl)nicotinonitrile (H-4)

Cream solid; M.P.: 266-268 ˚C; R_f: 0.47; *(n*-Hexane: Ethyl acetate 7:3); FT-IR (KBr, cm^{-1}): 3427, 3067, 2931, 2217, 1608, 1580, 1514, 1198; ^{1}H NMR (400 MHz, DMSO-d_6) δ_{ppm} 9.15 (d, J = 2.0 Hz, 1H), 8.55 (dd, J = 8.7, 2.0 Hz, 1H), 8.34 (s, 1H), 8.29 (d, J = 6.6 Hz, 1H), 8.10 (d, J = 8.5 Hz, 2H), 7.91 – 7.85 (m, 3H), 7.77 (d, J = 8.1 Hz, 1H), 7.71 (d, J = 8.6 Hz, 2H), 7.59 (t, J = 7.1 Hz, 1H), 7.47 (t, J = 7.1 Hz, 1H), 7.21 (d, J = 8.9 Hz, 2H), 3.89 (s, 3H). ^{13}C NMR (101 MHz, DMSO-d_6) δ_{ppm} 160.8, 158.1, 156.9, 154.4, 136.8, 131.2, 130.7, 128.5, 127.5, 124.3, 123.5, 122.6, 121.6, 120.9, 114.3, 112.1, 111.9, 105.8, 55.4. HR-MS m/z (%); Calc. for $C_{31}H_{19}ClN_2O_2$: 486.1135, found: 487.1212.

2-(4-Chlorophenyl)-6-(dibenzo[b,d]furan-2-yl)-4-(p-tolyl)nicotinonitrile (H-5)

Cream solid; M.P.: 268-270 ˚C; R_f: 0.48; *(n*-Hexane: Ethyl acetate 8:2); FT-IR (KBr, cm^{-1}): 3053, 2215, 1581, 1527, 1199; ^{1}H NMR (400 MHz, DMSO-d_6) δ_{ppm} 9.13 (d, J = 2.0 Hz, 1H), 8.52 (dd,

J = 8.8, 2.1 Hz, 1H), 8.32 (s, 1H), 8.27 (d, *J* = 7.4 Hz, 1H), 8.08 (d, *J* = 8.6 Hz, 2H), 7.86 (d, *J* = 8.7 Hz, 1H), 7.79 – 7.74 (m, 3H), 7.70 (d, *J* = 8.6 Hz, 2H), 7.58 (t, *J* = 7.3 Hz, 1H), 7.48 – 7.43 (m, 3H), 2.45 (s, 3H). ^{13}C NMR (101 MHz, DMSO-d_6) δ_{ppm} 160.3, 158.2, 156.9, 156.0, 154.8, 139.9, 136.7, 135.0, 133.4, 132.2, 131.2, 129.0, 129.0, 128.5, 128.1, 127.5, 124.3, 123.5, 123.4, 121.5, 120.9, 118.9, 117.8, 112.1, 111.9, 103.9, 20.9. HR-MS m/z (%); Calc. for $C_{31}H_{19}ClN_2O$: 470.1186, found: 471.1268.

2-(4-Chlorophenyl)-6-(dibenzo[b,d]furan-2-yl)-4-(o-tolyl)nicotinonitrile (H-6)

Cream solid; M.P.: 204-206 ˚C; R_f: 0.48; (*n*-Hexane: Ethyl acetate 8:2); FT-IR (KBr, cm^{-1}): 3060, 2924, 2217, 1582, 1530, 1199; ^{1}H NMR (400 MHz, DMSO-d_6) δ_{ppm} 9.17 (s, 1H), 8.57 (dd, *J* = 8.7, 2.0 Hz, 1H), 8.33 (s, 1H), 8.26 (d, *J* = 7.5 Hz, 1H), 8.11 (d, *J* = 8.5 Hz, 2H), 7.87 (d, *J* = 8.8 Hz, 1H), 7.76 (d, *J* = 8.3 Hz, 1H), 7.72 (d, *J* = 8.2 Hz, 2H), 7.58 (t, *J* = 7.7 Hz, 1H), 7.50 – 7.45 (m, 4H), 7.44 – 7.41 (m, 1H), 2.32 (s, 3H). ^{13}C NMR (101 MHz, DMSO-d_6) δ_{ppm} 159.6, 158.0, 157.0, 156.1, 155.6, 136.5, 136.5, 135.2, 135.1, 132.0, 131.1, 130.4, 129.5, 129.2, 128.6, 128.1, 127.4, 126.1, 124.4, 123.5, 123.4, 121.6, 120.9, 119.5, 117.0, 112.2, 111.9, 105.3, 19.6. HR-MS m/z (%); Calc. for $C_{31}H_{19}ClN_2O$: 470.1186, found: 471.1265.

2,4-bis(4-chlorophenyl)-6-(dibenzo[b,d]furan-2-yl)nicotinonitrile (H-7)

White solid; M.P.: >300 ˚C; R_f : 0.48; (*n*-Hexane: Ethyl acetate 8:2); FT-IR (KBr, cm^{-1}): 0, ^{1}H NMR (400 MHz, DMSO-d_6) δ_{ppm} 9.13 (d, *J* = 2.0 Hz, 1H), 8.53 (dd, *J* = 8.7, 2.0 Hz, 1H), 8.36 (s, 1H), 8.26 (d, *J* = 7.0 Hz, 1H), 8.09 (d, *J* = 8.6 Hz, 2H), 7.91 (d, *J* = 8.5 Hz, 2H), 7.86 (d, *J* = 8.7 Hz, 1H), 7.76 – 7.73 (m, 2H), 7.73 – 7.68 (m, 3H), 7.57 (t, *J* = 7.1 Hz, 1H), 7.46 (t, *J* = 7.5 Hz, 1H). ^{13}C NMR (101 MHz, DMSO-d_6) δ_{ppm} 160.2, 158.3, 157.0, 156.1, 153.5, 135.1, 135.0, 132.1, 131.2, 131.0, 128.9, 128.6, 127.5, 123.5, 123.4, 121.5, 121.0, 119.0, 112.2, 111.9, 104.0. HR-MS m/z (%); Calc. for $C_{30}H_{16}C_{l2}N_2O$: 490.0640, found: 490.0719.

2-(4-Chlorophenyl)-6-(dibenzo[b,d]furan-2-yl)-4-phenylnicotinonitrile (H-8)

Cream solid; M.P.: 272-274 ˚C; R_f: 0.48; (*n*-Hexane: Ethyl acetate 8:2); FT-IR (KBr, cm^{-1}): 3061, 2217, 1578, 1529, 1199; ^{1}H NMR (400 MHz, DMSO-d_6) δ_{ppm} 9.15 (s, 1H), 8.54 (d, *J* = 8.7 Hz, 1H), 8.36 (s, 1H), 8.27 (d, *J* = 7.7 Hz, 1H), 8.09 (d, *J* = 8.5 Hz, 2H), 7.90 – 7.85 (m, 3H), 7.76 (d, *J* = 8.2 Hz, 1H), 7.71 (d, *J* = 8.5 Hz, 2H), 7.67 – 7.62 (m, 3H), 7.57 (t, *J* = 8.2 Hz, 1H), 7.46 (t, *J*

= 7.5 Hz, 1H). ^{13}C NMR (101 MHz, DMSO-d_6) δ_{ppm} 160.30, 158.25, 156.94, 154.83, 136.69, 136.36, 135.03, 132.16, 131.22, 129.98, 129.98, 129.12, 129.06, 128.81, 128.55, 128.55, 128.13, 127.52, 124.34, 123.51, 121.56, 120.96, 119.07, 117.62, 112.15, 111.89, 104.05. HR-MS m/z (%); Calc. for $C_{30}H_{17}ClN_2O$: 456.1029, found: 457.1107.

2-(4-Chlorophenyl)-6-(dibenzo[b,d]furan-2-yl)-4-(4-hydroxy-3-ethoxyphenyl)nicotinonitrile (H-9)

Cream solid; M.P.: 276-278 ˚C; R_f: 0.39; *(n-*Hexane: Ethyl acetate 7:3); FT-IR (KBr, cm^{-1}): 3460, 3063, 2977, 2217, 1595, 1518, 1198; ^1H NMR (400 MHz, DMSO-d_6) δ_{ppm} 9.61 (s, 1H), 9.14 (d, *J* = 2.0 Hz, 1H), 8.53 (dd, *J* = 8.7, 1.9 Hz, 1H), 8.32 (s, 1H), 8.29 (d, *J* = 6.9 Hz, 1H), 8.10 (d, *J* = 8.6 Hz, 2H), 7.88 (d, *J* = 8.7 Hz, 1H), 7.77 (d, *J* = 8.2 Hz, 1H), 7.71 (d, *J* = 8.6 Hz, 2H), 7.59 (t, *J* = 7.1 Hz, 1H), 7.51 – 7.46 (m, 2H), 7.34 (dd, *J* = 8.2, 2.1 Hz, 1H), 7.03 (d, *J* = 8.2 Hz, 1H), 4.18 (q, *J* = 6.9 Hz, 2H), 1.41 (t, *J* = 7.0 Hz, 3H). ^{13}C NMR (101 MHz, DMSO-d_6) δ_{ppm} 160.5, 158.0, 156.9, 156.1, 154.8, 148.9, 146.7, 136.9, 134.9, 132.4, 131.3, 128.5, 128.1, 127.0, 124.3, 123.5, 123.4, 122.3, 121.6, 120.9, 118.7, 118.1, 115.7, 114.4, 112.1, 111.9, 103.7, 64.1, 14.7. HR-MS m/z (%); Calc. for $C_{32}H_{21}ClN_2O_3$: 516.1241, found: 517.1165.

4-(3-Bromophenyl)-2-(4-chlorophenyl)-6-(dibenzo[b,d]furan-2-yl)nicotinonitrile (H-10)

Cream solid; M.P.: 274-276 ˚C; R_f: 0.48; *(n-*Hexane: Ethyl acetate 8:2); FT-IR (KBr, cm^{-1}): 3067, 2214, 1580, 1528, 1200; ^1H NMR (400 MHz, DMSO-d_6) δ_{ppm} 9.16 (d, *J* = 2.0 Hz, 1H), 8.56 (dd, *J* = 8.7, 1.9 Hz, 1H), 8.41 (s, 1H), 8.28 (d, *J* = 6.3 Hz, 1H), 8.14 – 8.11 (m, 2H), 8.10 (s, 1H), 7.89 (t, *J* = 7.4 Hz, 2H), 7.84 (dd, *J* = 8.1, 3.1 Hz, 1H), 7.77 (d, *J* = 8.2 Hz, 1H), 7.72 (d, *J* = 8.6 Hz, 2H), 7.62 (t, *J* = 7.9 Hz, 1H), 7.58 (t, *J* = 7.4 Hz, 1H), 7.47 (t, *J* = 7.5 Hz, 1H). ^{13}C NMR (101 MHz, DMSO-d_6) δ_{ppm} 160.1, 158.3, 157.0, 156.1, 153.1, 138.5, 136.5, 135.1, 132.7, 132.0, 131.6, 131.2, 130.8, 128.6, 128.2, 128.1, 127.5, 124.3, 123.5, 123.4, 122.0, 121.5, 121.0, 119.0, 117.4, 112.1, 111.9, 104.1.

2-(4-chlorophenyl)-6-(dibenzo[b,d]furan-2-yl)-4-(4-hydroxyphenyl)nicotinonitrile (H-11)

Cream solid; M.P.: >300 ˚C; R_f : 0.5; *(n-*Hexane: Ethyl acetate 7:3); FT-IR (KBr, cm^{-1}): 3391, 2225, 1578, 1518, 1199; ^1H NMR (400 MHz, DMSO-d_6) δ_{ppm} 10.09 (s, 1H), 9.13 (d, *J* = 2.0 Hz, 1H), 8.52 (dd, *J* = 8.7, 2.0 Hz, 1H), 8.31 – 8.27 (m, 2H), 8.07 (d, *J* = 8.6 Hz, 2H), 7.86 (d, *J* = 8.7

Hz, 1H), 7.78 – 7.74 (m, 3H), 7.70 (d, *J* = 8.6 Hz, 2H), 7.58 (t, *J* = 7.1 Hz, 1H), 7.47 (t, *J* = 7.5 Hz, 1H), 7.01 (d, *J* = 8.6 Hz, 2H). ^{13}C NMR (101 MHz, DMSO-d_6) δ_{ppm} 160.5, 159.3, 158.0, 156.8, 156.0, 154.7, 136.8, 134.9, 132.3, 131.2, 130.8, 130.7, 128.5, 128.1, 127.4, 126.8, 124.3, 123.5, 123.4, 121.6, 120.8, 118.6, 118.0, 115.6, 112.1, 111.9, 103.5. HR-MS m/z (%); Calc. for $C_{30}H_{17}ClN_2O_2$: 472.0979, found: 473.1057.

2-(4-Chlorophenyl)-6-(dibenzo[b,d]furan-2-yl)-4-(3-hydroxyphenyl)nicotinonitrile (H-12)

Cream solid; M.P.: 273-275°C; R_f: 0.5; (*n*-Hexane: Ethyl acetate 7:3); FT-IR (KBr, cm^{-1}): 3384, 3057, 2923, 2219, 1578, 1529, 1198; ^{1}H NMR (400 MHz, DMSO-d_6) δ_{ppm} 9.89 (s, 1H), 9.16 (d, *J* = 2.0 Hz, 1H), 8.54 (dd, *J* = 8.7, 2.0 Hz, 1H), 8.33 (s, 1H), 8.28 (d, *J* = 7.8 Hz, 1H), 8.08 (d, *J* = 8.6 Hz, 2H), 7.87 (d, *J* = 8.7 Hz, 1H), 7.76 (d, *J* = 8.1 Hz, 1H), 7.71 (d, *J* = 8.6 Hz, 2H), 7.58 (t, *J* = 7.1 Hz, 1H), 7.48 – 7.45 (m, 1H), 7.44 – 7.41 (m, 1H), 7.26 (d, *J* = 9.3 Hz, 1H), 7.22 (t, *J* = 2.1 Hz, 1H), 7.02 (dd, *J* = 7.7, 2.9 Hz, 1H). ^{13}C NMR (101 MHz, DMSO-d_6) δ_{ppm} 160.3, 158.1, 157.5, 156.9, 156.1, 154.9, 137.6, 136.7, 135.0, 132.1, 131.2, 129.9, 128.5, 128.1, 127.5, 124.3, 123.5, 123.4, 121.6, 120.9, 119.6, 118.9, 117.5, 116.8, 115.8, 115.7, 112.1, 111.9, 103.9. HR-MS m/z (%); Calc. for $C_{30}H_{17}ClN_2O_2$: 472.0979, found: 473.1058.

2-(4-Chlorophenyl)-6-(dibenzo[b,d]furan-2-yl)-4-(3-fluorophenyl)nicotinonitrile (H-13)

Cream solid; M.P.: 280-282 °C; R_f: 0.51; (*n*-Hexane: Ethyl acetate 8:2); FT-IR (KBr, cm^{-1}): 3080, 2219, 1581, 1524, 1201; ^{1}H NMR (400 MHz, DMSO-d_6) δ_{ppm} 9.16 (d, *J* = 2.1 Hz, 1H), 8.55 (dd, *J* = 8.7, 2.0 Hz, 1H), 8.38 (s, 1H), 8.28 (d, *J* = 6.6 Hz, 1H), 8.11 (d, *J* = 8.6 Hz, 2H), 7.97 (dd, *J* = 8.9, 5.4 Hz, 2H), 7.88 (d, *J* = 8.7 Hz, 1H), 7.77 (d, *J* = 8.1 Hz, 1H), 7.72 (d, *J* = 8.6 Hz, 2H), 7.59 (t, *J* = 7.1 Hz, 1H), 7.54 – 7.50 (m, 2H), 7.49 – 7.45 (m, 1H). ^{13}C NMR (101 MHz, DMSO-d_6) δ_{ppm} 160.2, 158.3, 156.9, 156.1, 153.7, 136.6, 135.1, 132.8, 132.1, 131.6, 131.5, 131.2, 128.6, 128.1, 127.5, 124.3, 123.5, 123.4, 121.5, 121.0, 119.1, 117.6, 115.9, 115.7, 112.2, 111.9, 104.1. HR-MS m/z (%); Calc. for $C_{30}H_{16}ClFN_2O$: 474.0935, found: 475.1014.

4-(4-Bromophenyl)-2-(4-chlorophenyl)-6-(dibenzo[b,d]furan-2-yl)nicotinonitrile (H-14)

Cream solid; M.P.: >300 °C; R_f: 0.48; (*n*-Hexane: Ethyl acetate 8:2); FT-IR (KBr, cm^{-1}): 3061, 2218, 1593, 1529, 1199; ^{1}H NMR (400 MHz, DMSO-d_6) δ_{ppm} 9.15 (d, *J* = 2.1 Hz, 1H), 8.55 (dd, *J* = 8.8, 1.9 Hz, 1H), 8.39 (s, 1H), 8.28 (d, *J* = 6.7 Hz, 1H), 8.10 (d, *J* = 8.6 Hz, 2H), 7.90 – 7.83

(m, 5H), 7.77 (d, *J* = 8.2 Hz, 1H), 7.72 (d, *J* = 8.6 Hz, 2H), 7.59 (t, *J* = 7.1 Hz, 1H), 7.47 (t, *J* = 7.5 Hz, 1H). ^{13}C NMR (101 MHz, DMSO-d_6) δ_{ppm} 160.2, 158.3, 157.0, 156.1, 153.6, 136.6, 135.5, 135.1, 131.8, 131.2, 128.6, 128.2, 127.5, 124.3, 123.8, 123.5, 123.4, 121.5, 121.0, 119.0, 117.5, 112.2, 111.9, 111.6, 104.0. HR-MS m/z (%); Calc. for $C_{30}H_{16}BrClN_2O$: 534.0135, found: 535.0215.

2-(4-chlorophenyl)-6-(dibenzo[b,d]furan-2-yl)-4-(4-(dimethylamino)phenyl)nicotinonitrile (H-15)

Yellow solid; M.P.: 262-264 ˚C; R_f: 0.3; (*n*-Hexane: Ethyl acetate 8:2); FT-IR (KBr, cm^{-1}): 3071, 2888, 2215, 1611, 1579, 1527, 1199; ^{1}H NMR (400 MHz, DMSO-d_6) δ_{ppm} 9.13 (d, *J* = 2.0 Hz, 1H), 8.52 (dd, *J* = 8.7, 1.9 Hz, 1H), 8.30 (d, *J* = 6.2 Hz, 1H), 8.28 (s, 1H), 8.08 (d, *J* = 8.6 Hz, 2H), 7.87 (d, *J* = 8.7 Hz, 1H), 7.80 (d, *J* = 9.0 Hz, 2H), 7.77 (d, *J* = 8.1 Hz, 1H), 7.70 (d, *J* = 8.6 Hz, 2H), 7.59 (t, *J* = 7.0 Hz, 1H), 7.47 (t, *J* = 7.5 Hz, 1H), 6.93 (d, *J* = 9.1 Hz, 2H), 3.05 (s, 6H). ^{13}C NMR (101 MHz, DMSO-d_6) δ_{ppm} 157.9, 156.8, 156.1, 154.8, 151.4, 137.0, 134.8, 132.5, 131.2, 130.2, 128.4, 128.1, 127.4, 124.3, 123.5, 122.8, 121.6, 118.4, 118.1, 112.1, 111.8, 107.2, 39.8. HR-MS m/z (%); Calc. for $C_{32}H_{22}ClN_3O$: 499.1451, found: 500.1533.

4,4'-(1,3-phenylene)bis(2-(4-chlorophenyl)-6-(dibenzo[b,d]furan-2-yl)nicotinonitrile) (H-16)

Cream solid; M.P.: >300 ˚C; R_f: 0.65; (*n*-Hexane: Ethyl acetate 7:3); FT-IR (KBr, cm^{-1}): 3390, 3063, 2219, 2193, 1623, 1578, 1527, 1198; ^{1}H NMR (400 MHz, DMSO-d_6) δ_{ppm} 9.27 – 9.07 (m, 2H), 8.37 – 8.32 (m, 2H), 8.11 – 8.09 (m, 2H), 7.90 – 7.87 (m, 2H), 7.75 – 7.70 (m, 6H), 7.59 – 7.55 (m, 6H), 7.47 – 7.42 (m, 8H). ^{13}C NMR (101 MHz, DMSO-d_6) δ_{ppm} 160.4, 158.2, 156.9, 147.4, 141.9, 136.7, 132.2, 131.2, 130.2, 128.6, 124.4, 123.7, 121.6, 117.9, 115.7, 111.9, 104.3.

2-(4-Chlorophenyl)-6-(dibenzo[b,d]furan-2-yl)-4-(2,4-dichlorophenyl)nicotinonitrile (H-17)

Cream solid; M.P.: 262-264 ˚C; R_f: 0.42; (*n*-Hexane: Ethyl acetate 8:2); FT-IR (KBr, cm^{-1}): 3086, 2214, 1580, 1473, 1200; ^{1}H NMR (400 MHz, DMSO-d_6) δ_{ppm} 9.16 (s, 1H), 8.56 (dd, *J* = 8.8, 1.9 Hz, 1H), 8.43 (s, 1H), 8.26 (d, *J* = 7.5 Hz, 1H), 8.11 (d, *J* = 8.6 Hz, 2H), 7.99 (d, *J* = 2.1 Hz, 1H), 7.90 (d, *J* = 8.9 Hz, 1H), 7.82 – 7.77 (m, 2H), 7.77 – 7.72 (m, 3H), 7.59 (t, *J* = 7.8 Hz, 1H), 7.48 (t, *J* = 7.4 Hz, 1H). ^{13}C NMR (101 MHz, DMSO-d_6) δ_{ppm} 159.4, 158.5, 157.1, 151.9, 136.1, 135.4, 132.6, 131.8, 131.0, 129.3, 128.8, 128.2, 127.9, 127.5, 124.4, 123.6, 123.3, 121.5, 121.0, 116.6, 112.3, 111.9, 105.2. HR-MS m/z (%); Calc. for $C_{30}H_{15}Cl_3N_2O$: 524.0250, found: 525.0325.

2-(4-Chlorophenyl)-6-(dibenzo[b,d]furan-2-yl)-4-(naphthalen-1-yl)nicotinonitrile (H-18)

Cream solid; M.P.: 220-222 ˚C; R_f: 0.5; *(n*-Hexane: Ethyl acetate 8:2); FT-IR (KBr, cm^{-1}): 3057, 2220, 1578, 1527, 1198; ^1H NMR (400 MHz, DMSO-d_6) δ_{ppm} 9.18 (d, J = 2.1 Hz, 1H), 8.60 (dd, J = 8.9, 2.0 Hz, 1H), 8.46 (s, 1H), 8.24 (d, J = 6.3 Hz, 1H), 8.18 (d, J = 7.5 Hz, 1H), 8.16 – 8.11 (m, 3H), 7.88 (d, J = 8.9 Hz, 1H), 7.77 (d, J = 2.6 Hz, 2H), 7.76 – 7.74 (m, 2H), 7.72 (s, 1H), 7.68 – 7.64 (m, 1H), 7.64 – 7.58 (m, 2H), 7.58 – 7.55 (m, 1H), 7.44 (t, J = 7.4 Hz, 1H). ^{13}C NMR (101 MHz, DMSO-d_6) δ_{ppm} 159.7, 158.0, 157.0, 156.1, 154.4, 136.5, 135.1, 134.3, 133.1, 132.0, 131.2, 130.3, 129.8, 128.6, 128.1, 127.4, 126.6, 125.4, 125.4, 125.0, 124.4, 123.5, 123.4, 121.6, 121.0, 120.3, 117.0, 112.2, 111.9, 106.0. HR-MS m/z (%); Calc. for $C_{34}H_{19}ClN_2O$: 506.1186, found: 507.1217.

4,4'-(1,4-phenylene)bis(2-(4-chlorophenyl)-6-(dibenzo[b,d]furan-2-yl)nicotinonitrile) (H-19)

Cream solid; M.P.: >300 ˚C; R_f: 0.65; *(n*-Hexane: Ethyl acetate 7:3); FT-IR (KBr, cm^{-1}): 3359, 3053, 2219, 2190, 1580, 1491, 1198; ^1H NMR (400 MHz, DMSO-d_6) δ_{ppm} 9.17 (s, 2H), 8.59 – 8.54 (m, 2H), 8.40 – 8.38 (m, 2H), 8.30 – 8.28 (m, 2H), 8.12 – 8.09 (m, 4H), 7.89 – 7.86 (m, 4H), 7.72 – 7.71 (m, 4H), 7.58 – 7.57 (m, 4H), 7.49 – 7.48 (m, 2H), 7.47 – 7.46 (m, 2H). ^{13}C NMR (101 MHz, DMSO-d_6) δ_{ppm} 160.3, 156.2, 153.2, 145.7, 142.6, 131.2, 128.5, 123.3, 121.6, 111.8.

2-(4-chlorophenyl)-6-(dibenzo[b,d]furan-2-yl)-4-(1H-indol-3-yl)nicotinonitrile (H-20)

Cream solid; M.P.: >300 ˚C; R_f: 0.3; *(n*-Hexane: Ethyl acetate 8:2); FT-IR (KBr, cm^{-1}): 3327, 3061, 2220, 1582, 1522, 1195; ^1H NMR (400 MHz, DMSO-d_6) δ_{ppm} 11.99 (d, J = 3.0 Hz, 1H), 9.10 (d, J = 2.0 Hz, 1H), 8.50 (dd, J = 8.7, 2.0 Hz, 1H), 8.41 (s, 1H), 8.31 (d, J = 6.3 Hz, 1H), 8.17 (d, J = 2.8 Hz, 1H), 8.12 (d, J = 8.6 Hz, 2H), 7.95 (d, J = 7.5 Hz, 1H), 7.89 (d, J = 8.7 Hz, 1H), 7.77 (d, J = 8.2 Hz, 1H), 7.72 (d, J = 8.5 Hz, 2H), 7.59 (d, J = 8.5 Hz, 2H), 7.46 (t, J = 7.6 Hz, 1H), 7.29 (t, J = 6.7 Hz, 1H), 7.25 (t, J = 7.2 Hz, 1H). ^{13}C NMR (101 MHz, DMSO-d_6) δ_{ppm} 160.9, 158.0, 156.8, 156.1, 149.2, 137.0, 136.5, 134.9, 132.7, 131.2, 128.5, 128.1, 127.4, 124.9, 124.3, 123.4, 122.4, 121.7, 120.7, 119.5, 118.6, 118.3, 112.4, 112.1, 111.9, 111.1, 103.2. HR-MS m/z (%); Calc. for $C_{32}H_{18}ClN_3O$: 495.1138, found: 496.1216.

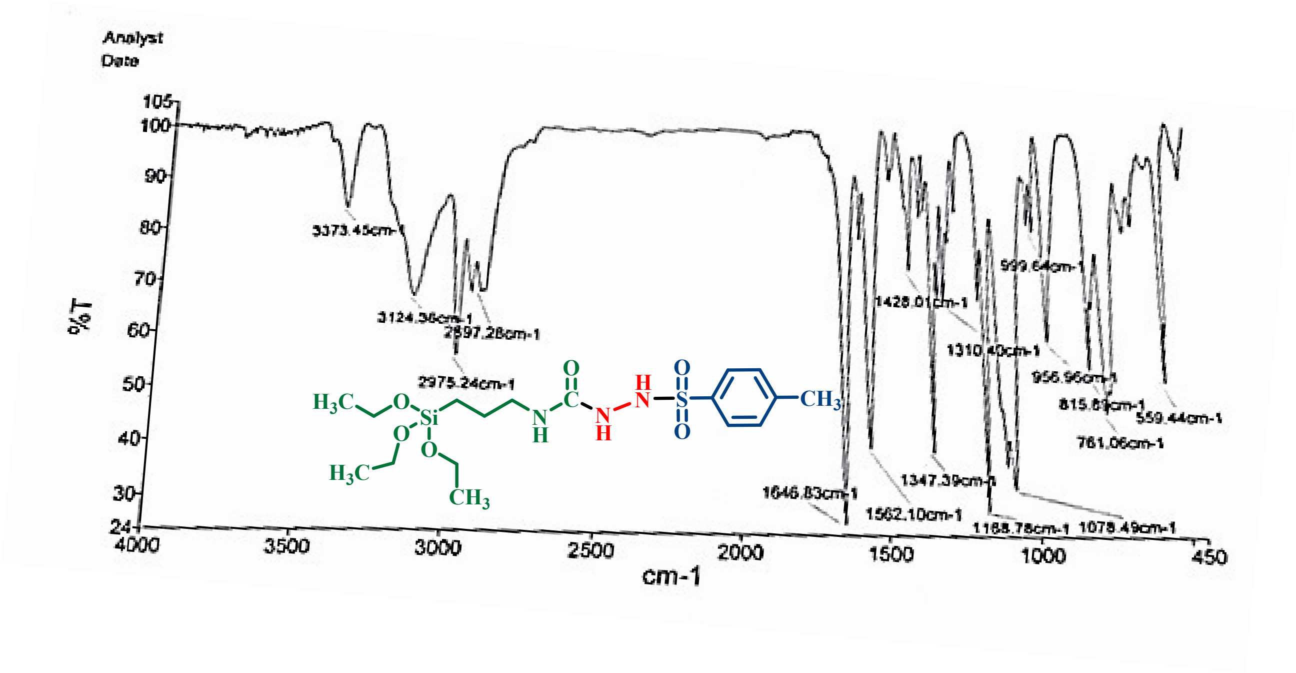

FT-IR spectrum of 2-tosyl-*N*-(3-(triethoxysilyl)propyl)hydrazine-1-carboxamide

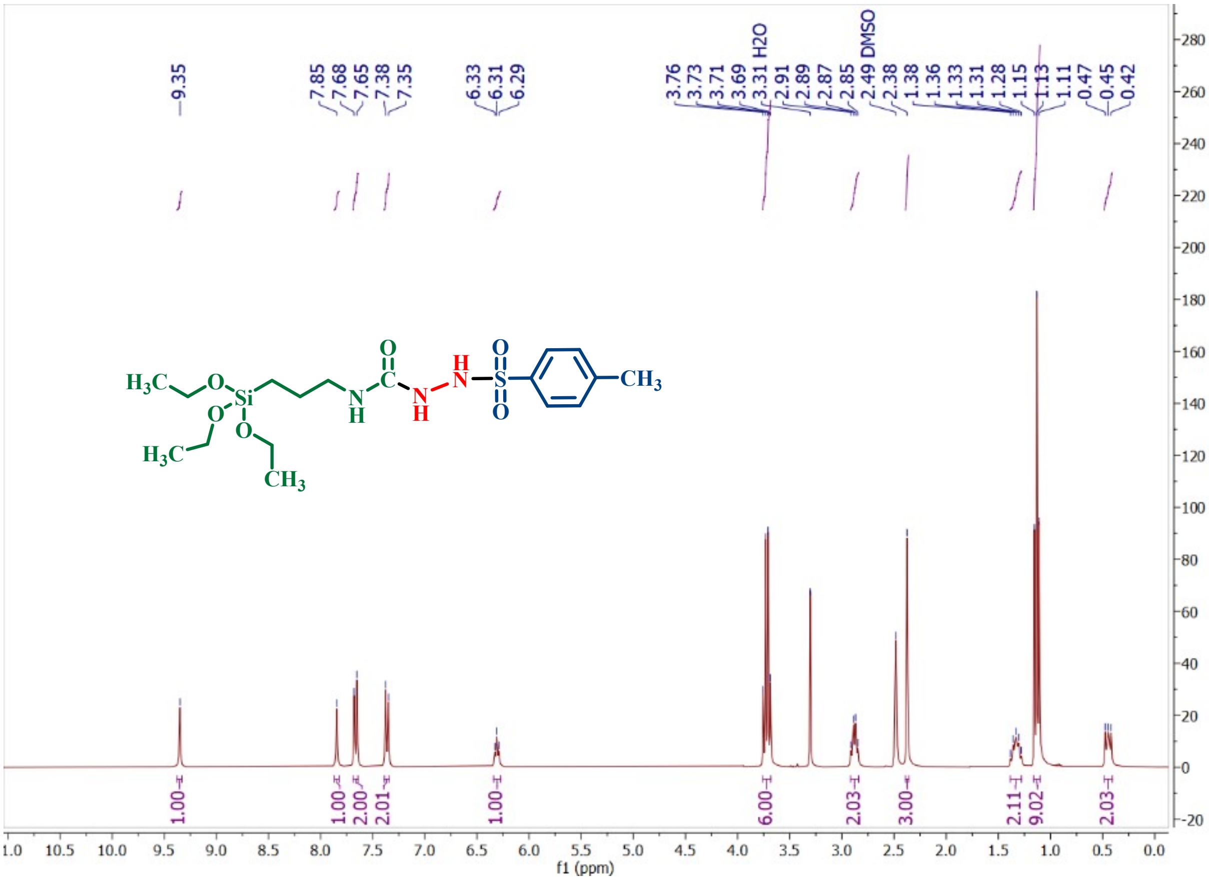

^{1}H-NMR spectrum of 2-tosyl-*N*-(3-(triethoxysilyl)propyl)hydrazine-1-carboxamide

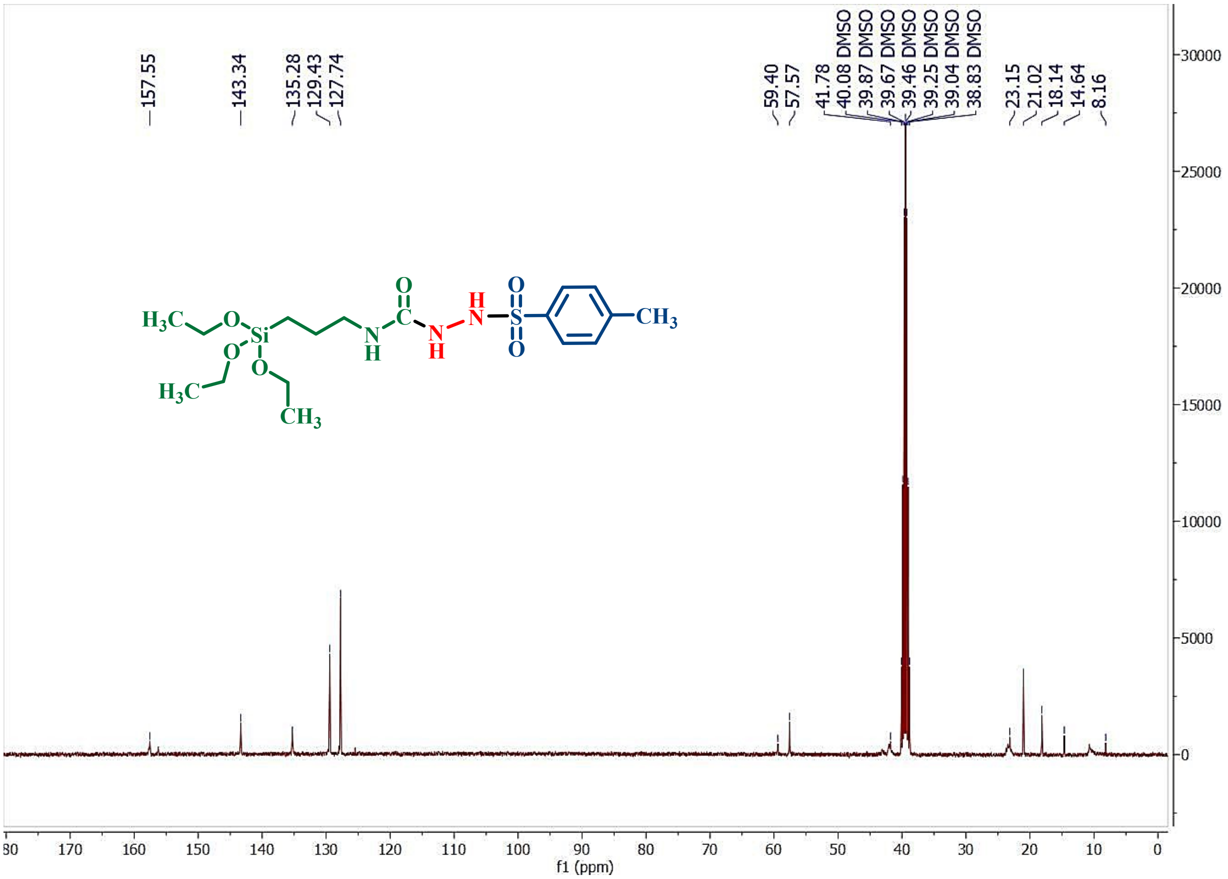

^{13}C-NMR spectrum of 2-tosyl-*N*-(3-(triethoxysilyl)propyl)hydrazine-1-carboxamide

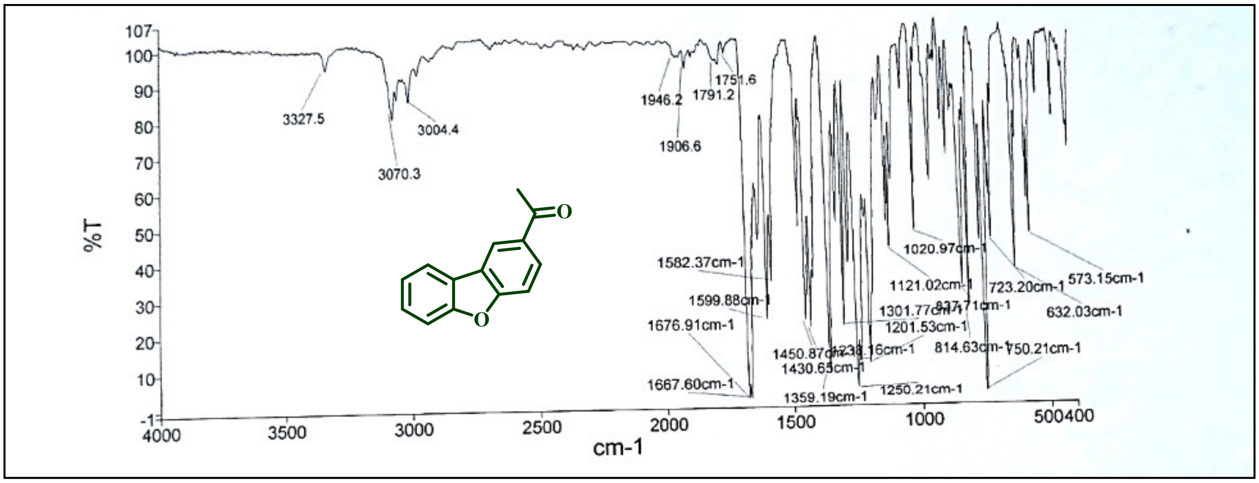

FT-IR spectrum of 1-(dibenzo[*b*,*d*]furan-2-yl)ethan-1-one

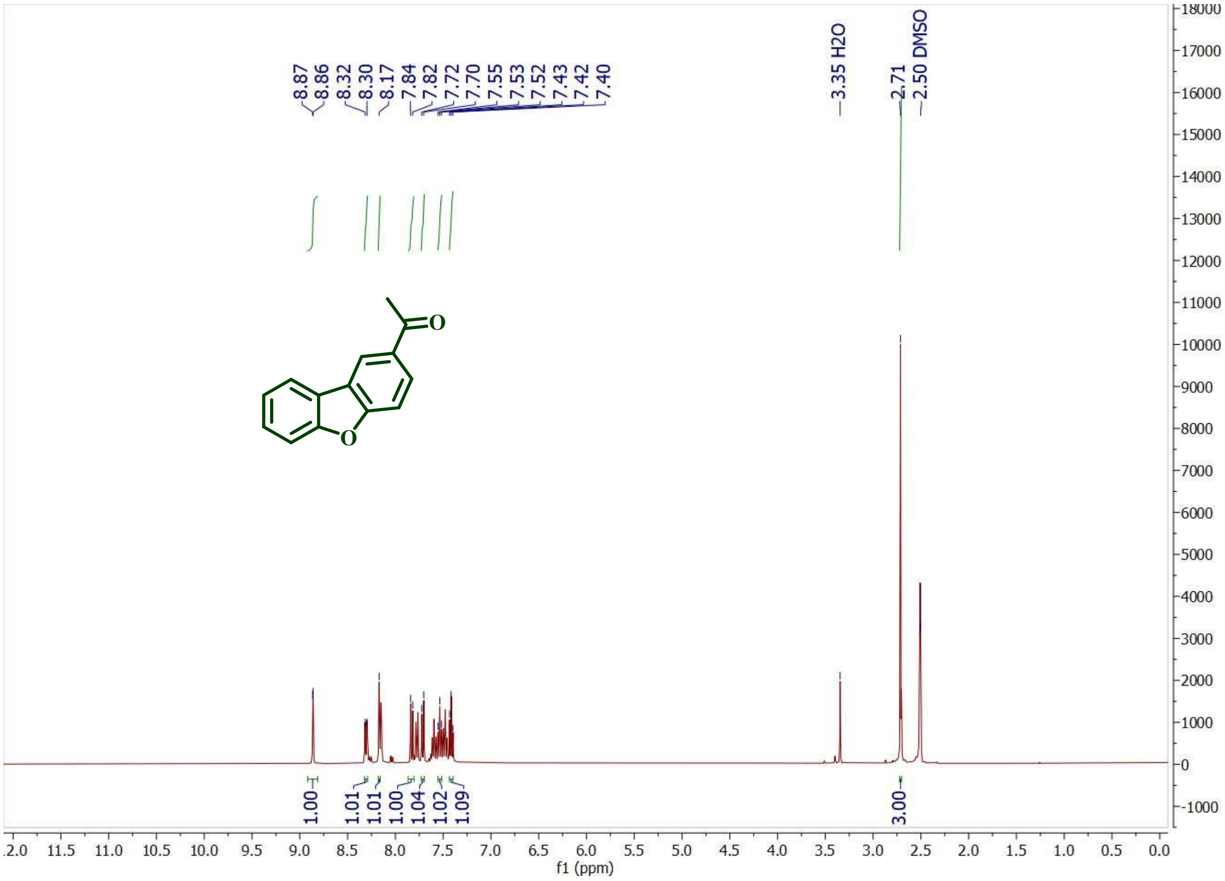

^{1}H-NMR spectrum of 1-(dibenzo[*b*,*d*]furan-2-yl)ethan-1-one

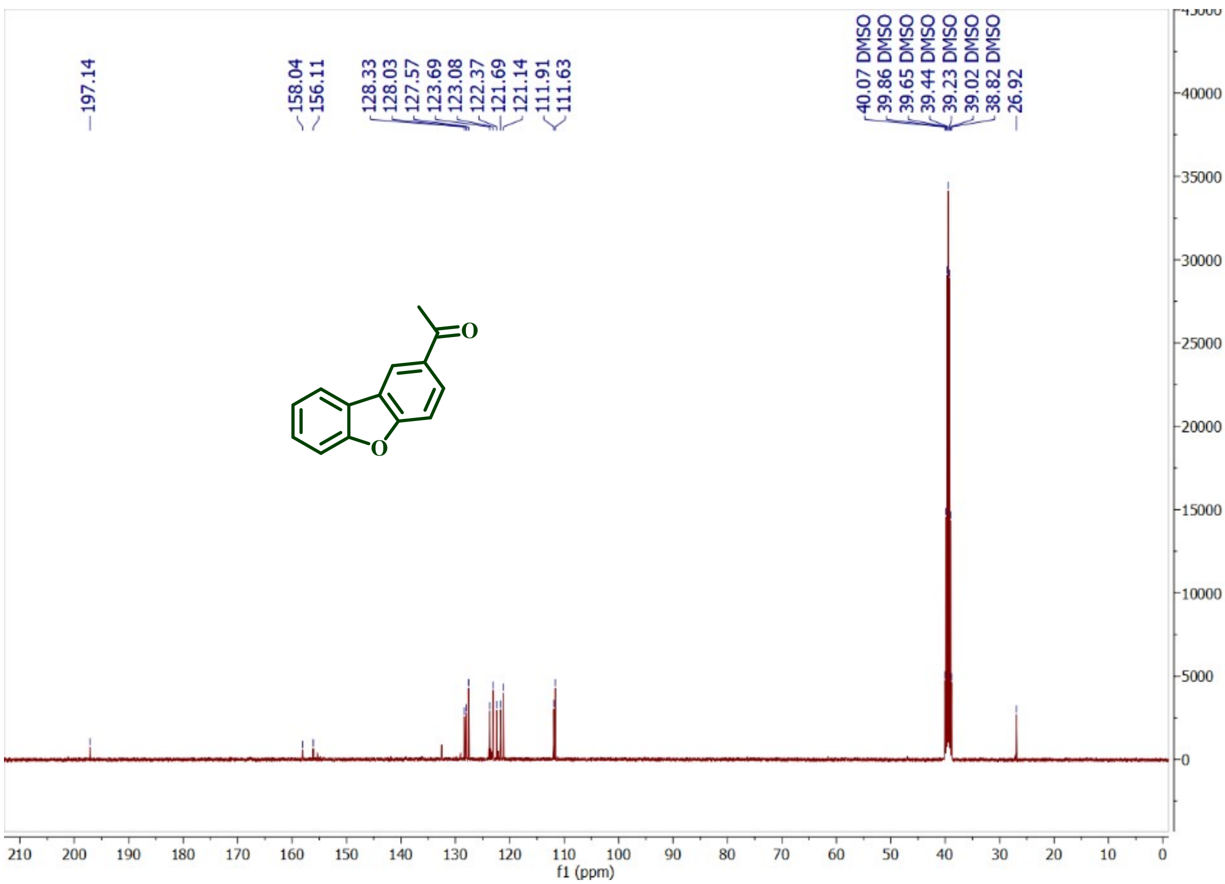

^{13}C-NMR spectrum of 1-(dibenzo[*b*,*d*]furan-2-yl)ethan-1-one

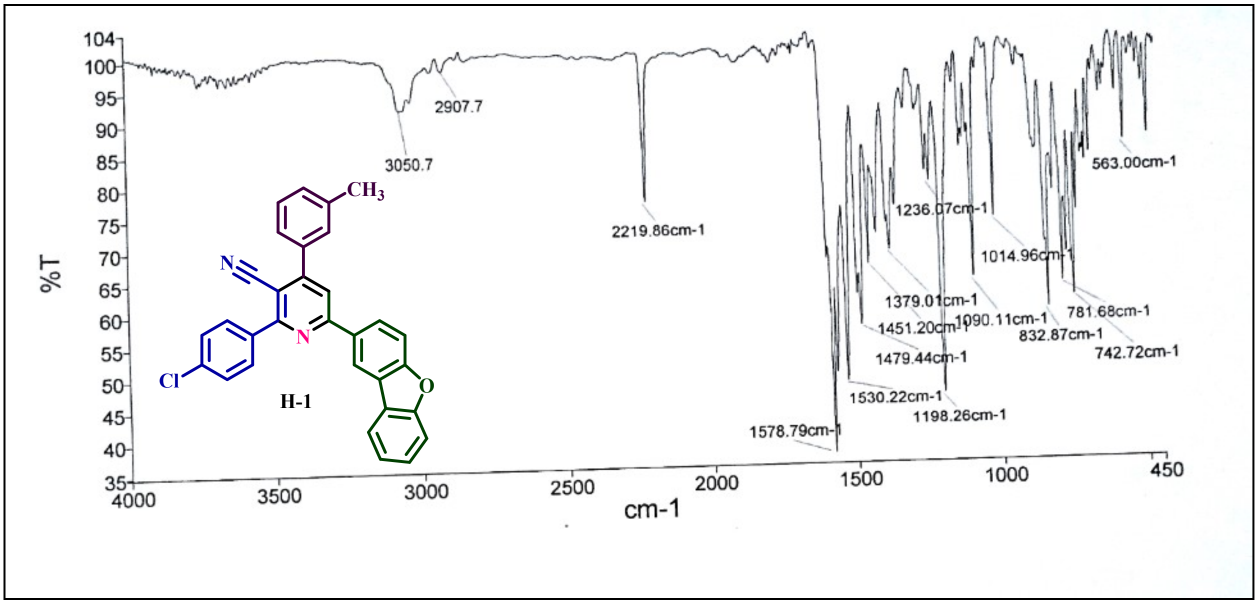

FT-IR spectrum of 2-(4-chlorophenyl)-6-(dibenzo[*b*,*d*]furan-2-yl)-4-(m-tolyl)nicotinonitrile (H-1)

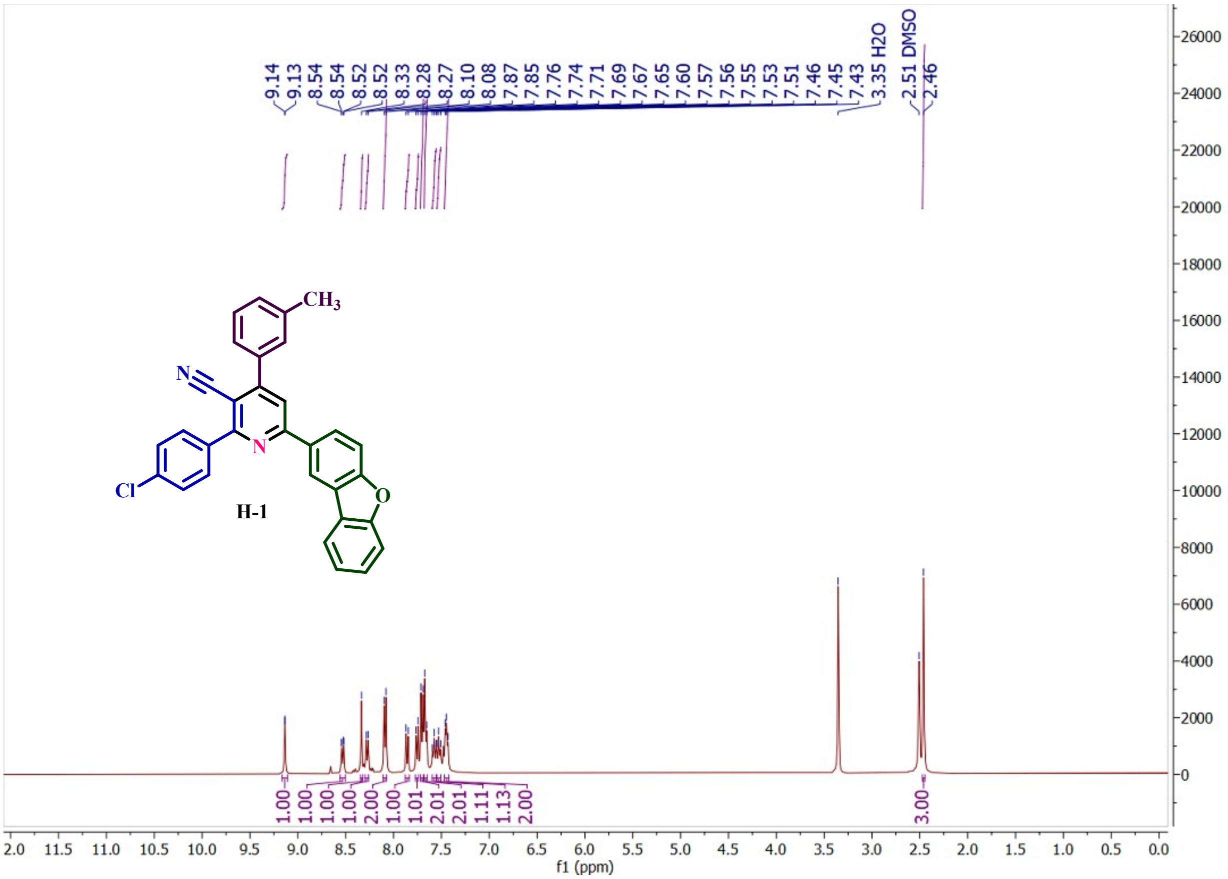

^{1}H-NMR spectrum of 2-(4-chlorophenyl)-6-(dibenzo[*b*,*d*]furan-2-yl)-4-(m-tolyl)nicotinonitrile (H-1)

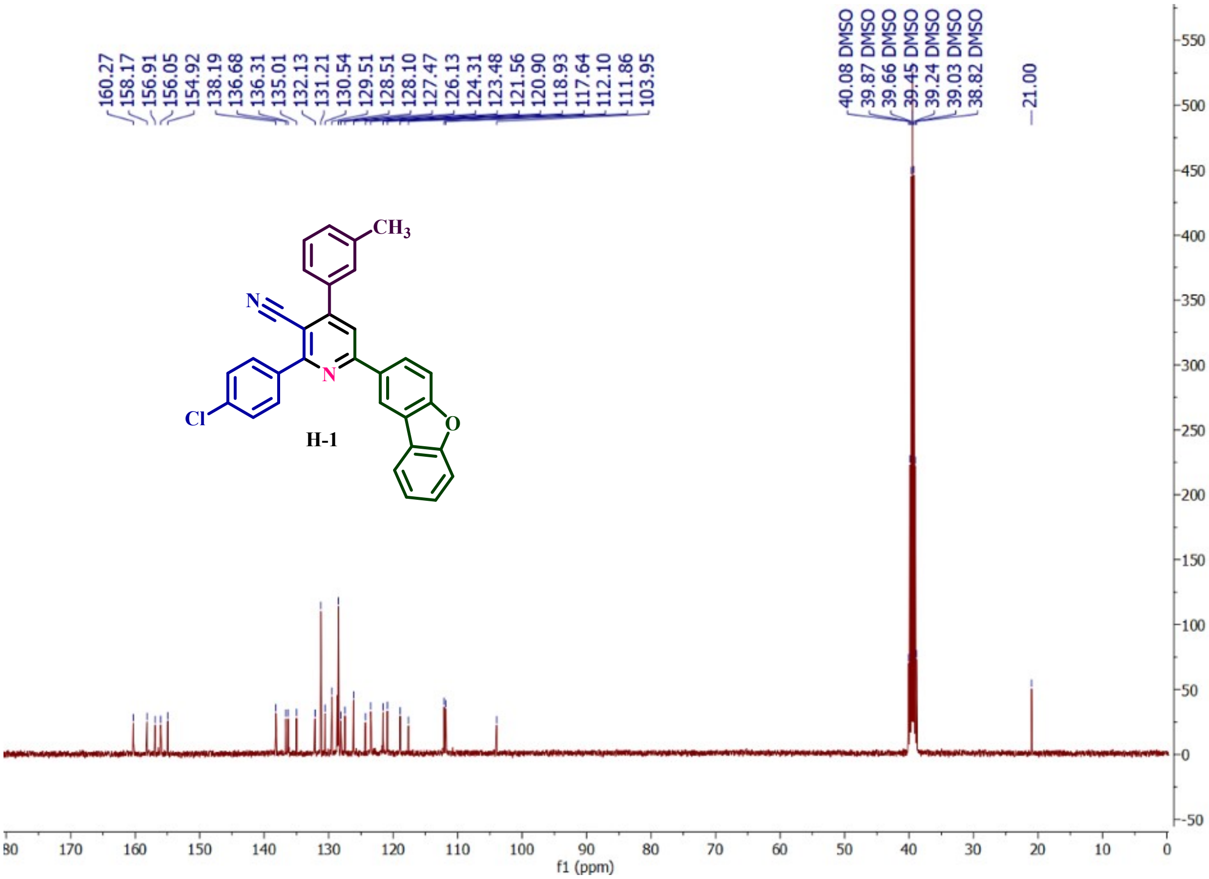

^{13}C-NMR spectrum of 2-(4-chlorophenyl)-6-(dibenzo[*b*,*d*]furan-2-yl)-4-(m-tolyl)nicotinonitrile (H-1)

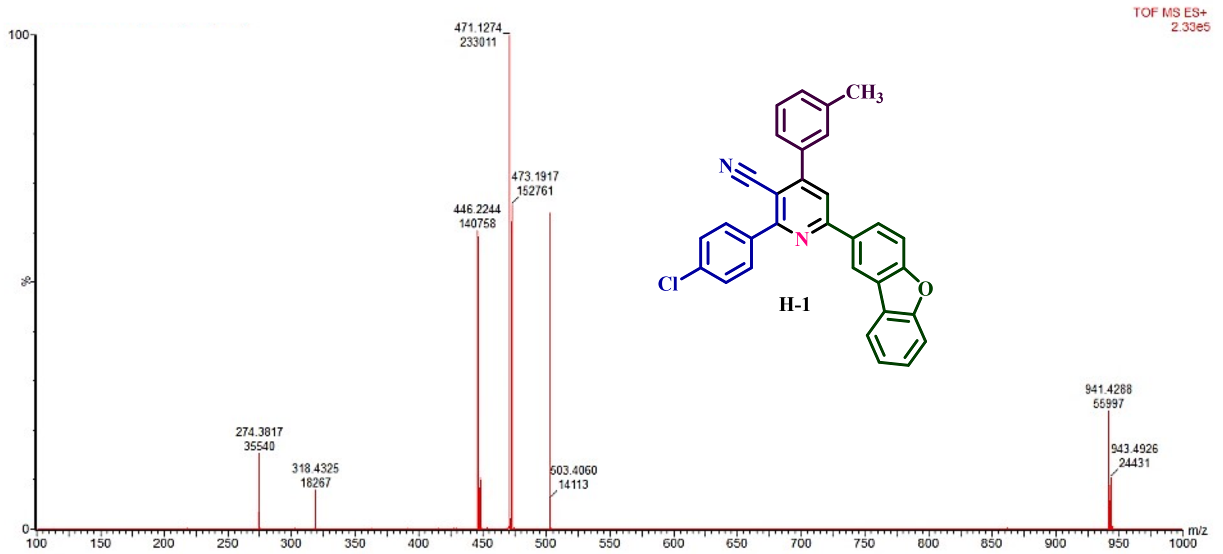

HR-Mass spectrum of 2-(4-chlorophenyl)-6-(dibenzo[*b*,*d*]furan-2-yl)-4-(m-tolyl)nicotinonitrile (H-1)

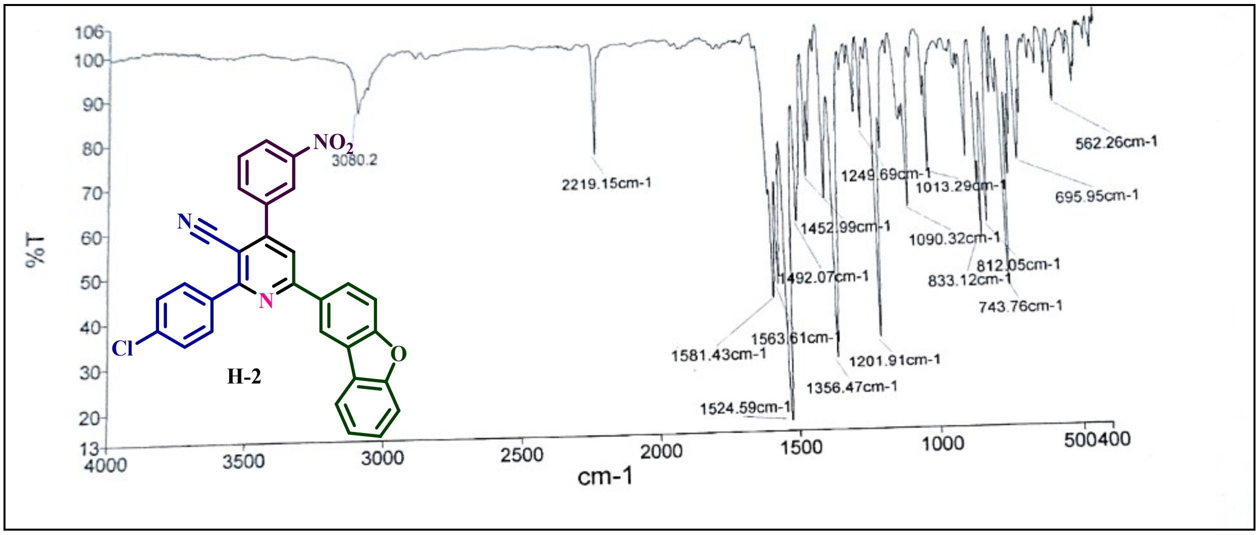

FT-IR spectrum of 2-(4-chlorophenyl)-6-(dibenzo[*b*,*d*]furan-2-yl)-4-(3-nitrophenyl)nicotinonitrile (H-2)

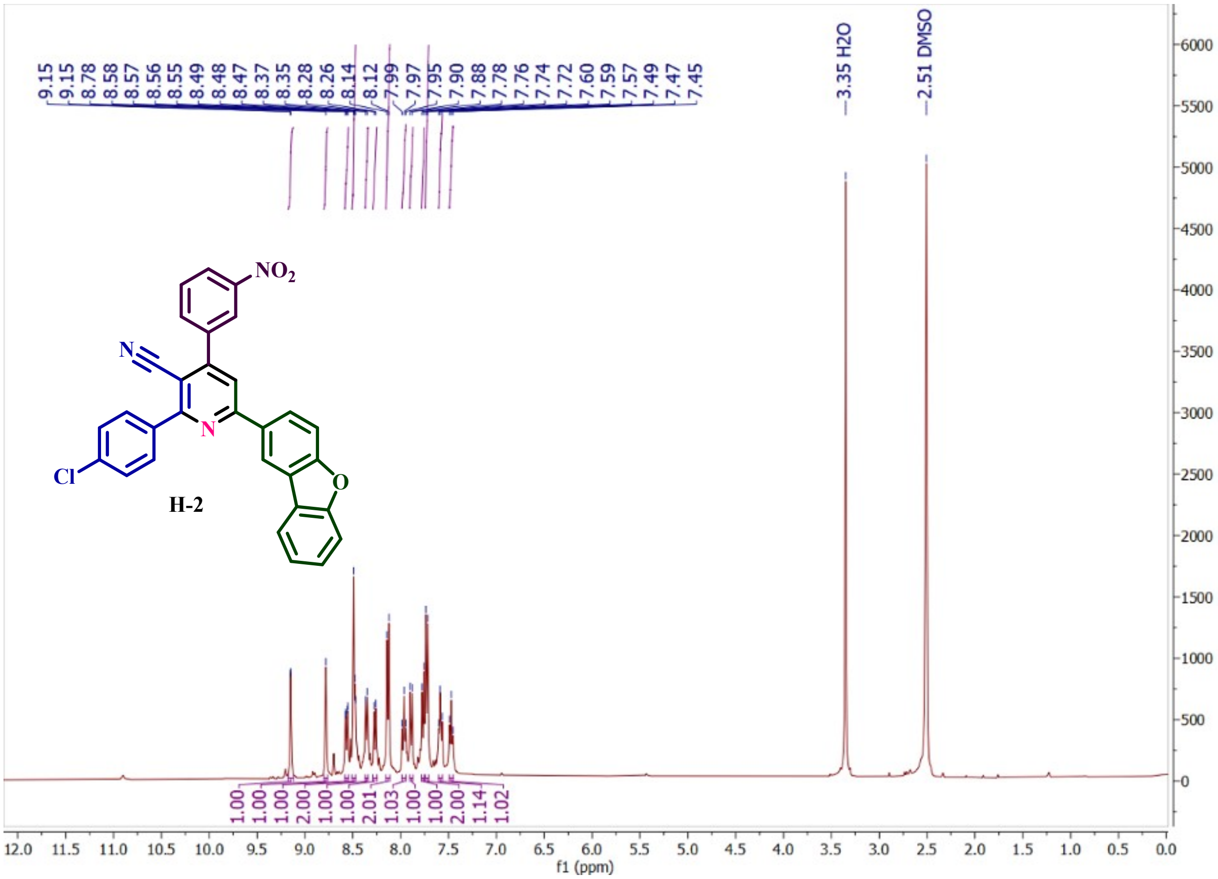

^{1}H-NMR spectrum of 2-(4-chlorophenyl)-6-(dibenzo[*b*,*d*]furan-2-yl)-4-(3-nitrophenyl)nicotinonitrile (H-2)

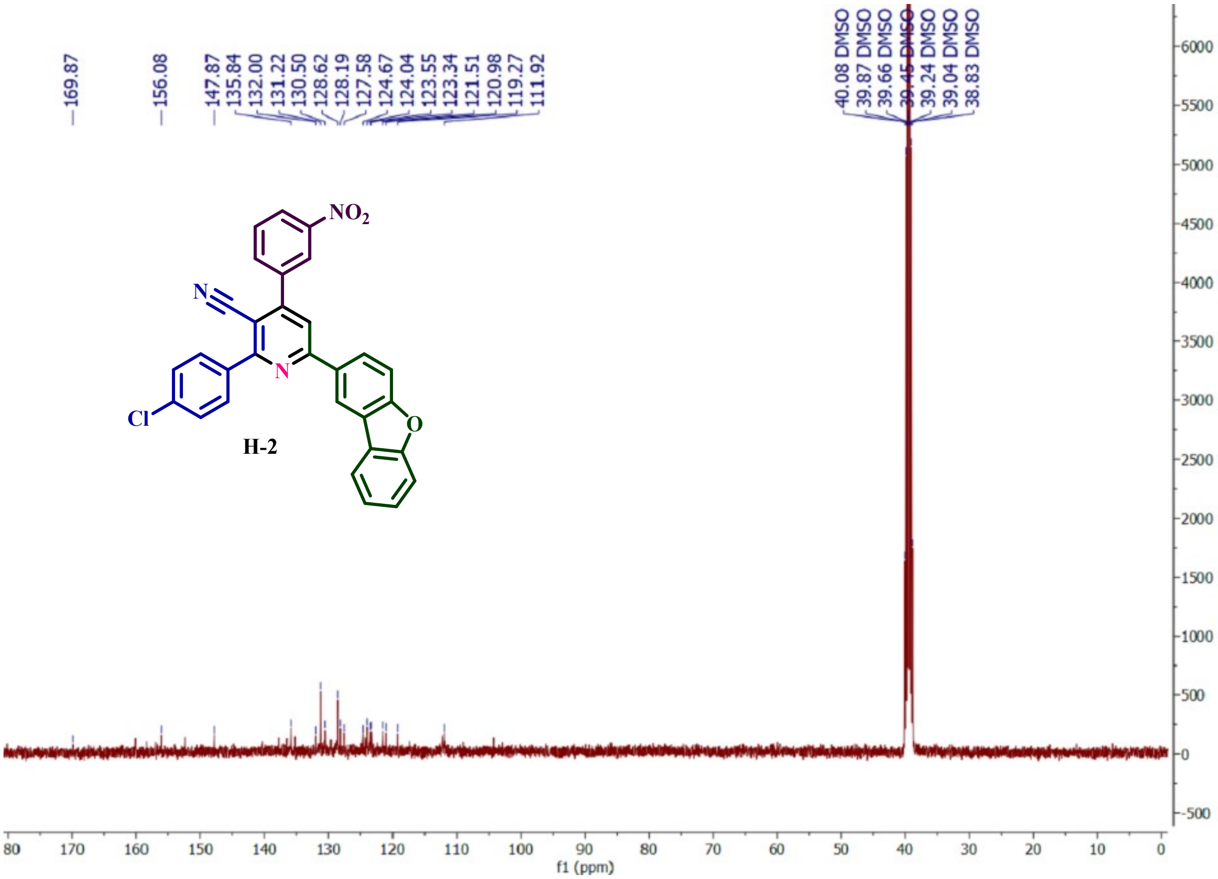

^{13}C-NMR spectrum of 2-(4-chlorophenyl)-6-(dibenzo[*b*,*d*]furan-2-yl)-4-(3-nitrophenyl)nicotinonitrile (H-2)

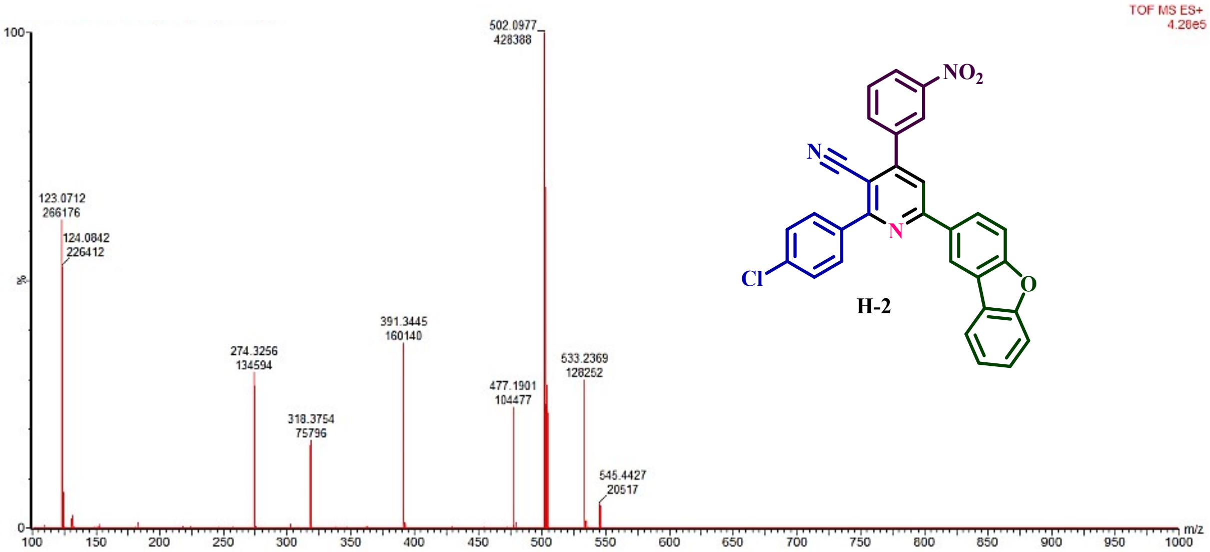

HR-Mass spectrum of 2-(4-chlorophenyl)-6-(dibenzo[*b*,*d*]furan-2-yl)-4-(3-nitrophenyl)nicotinonitrile (H-2)

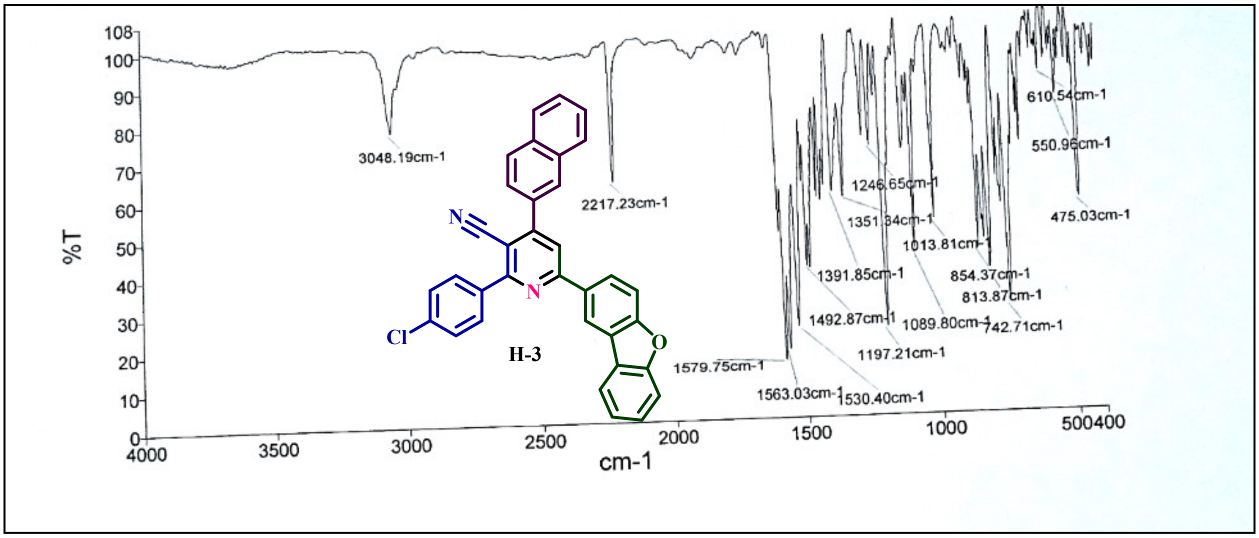

FT-IR spectrum of 2-(4-chlorophenyl)-6-(dibenzo[*b*,*d*]furan-2-yl)-4-(naphthalen-2-yl)nicotinonitrile (H-3)

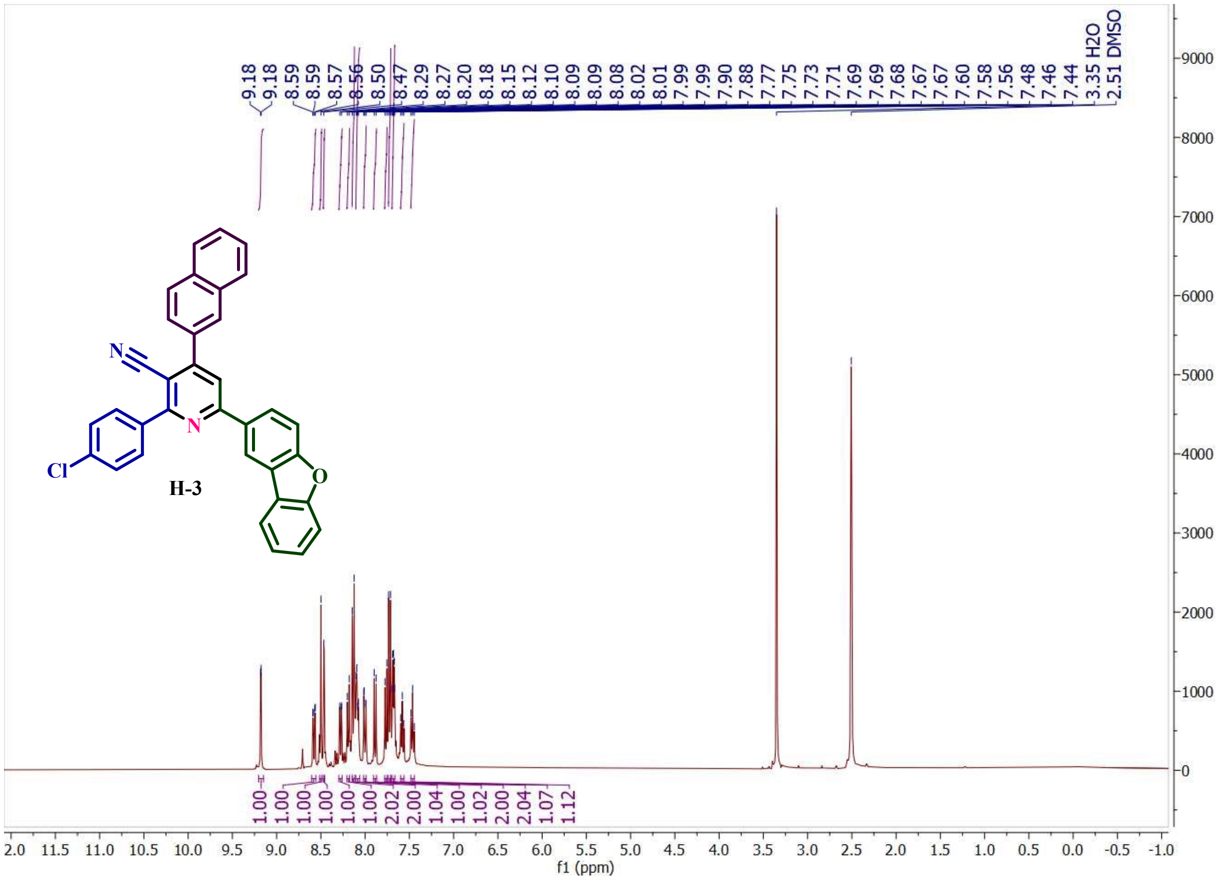

^{1}H-NMR spectrum of 2-(4-chlorophenyl)-6-(dibenzo[*b*,*d*]furan-2-yl)-4-(naphthalen-2-yl)nicotinonitrile (H-3)

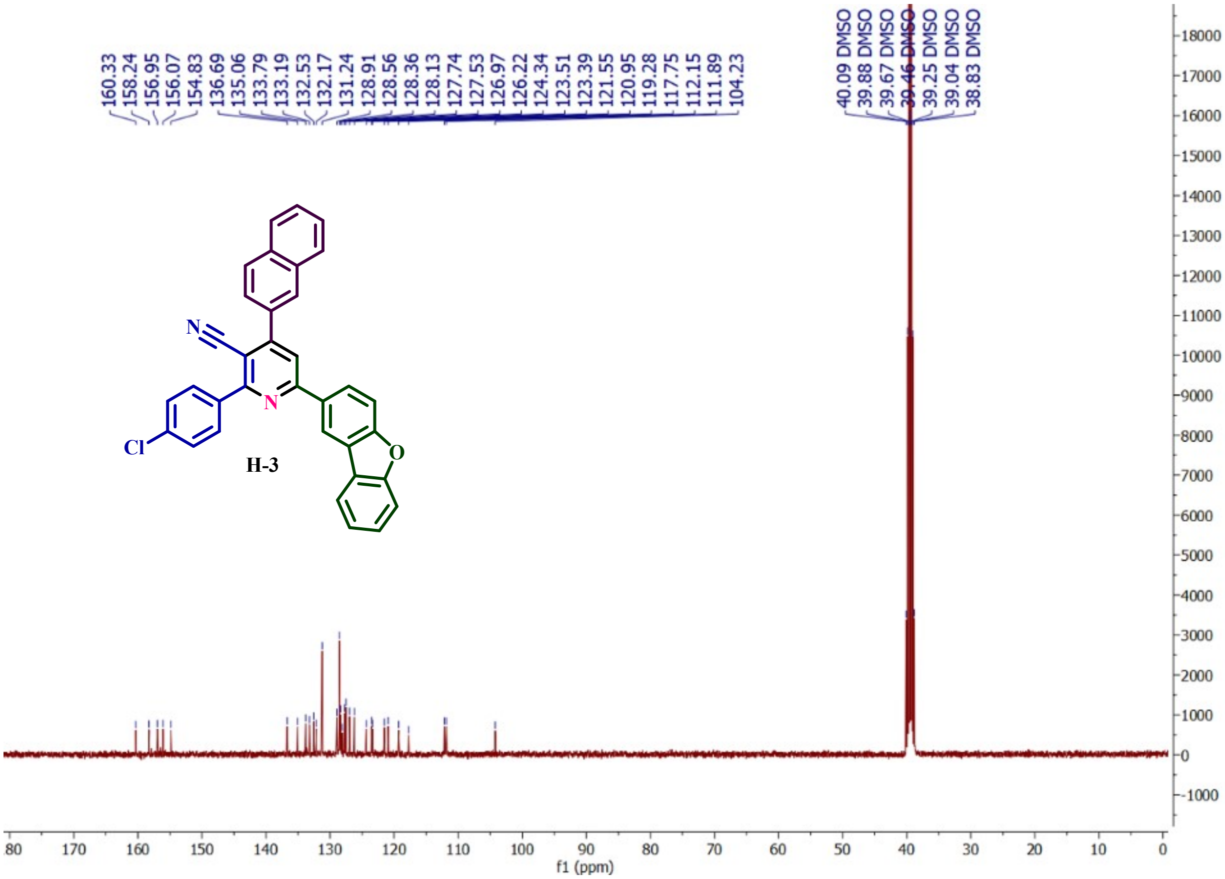

^{13}C-NMR spectrum of 2-(4-chlorophenyl)-6-(dibenzo[*b*,*d*]furan-2-yl)-4-(naphthalen-2-yl)nicotinonitrile (H-3)

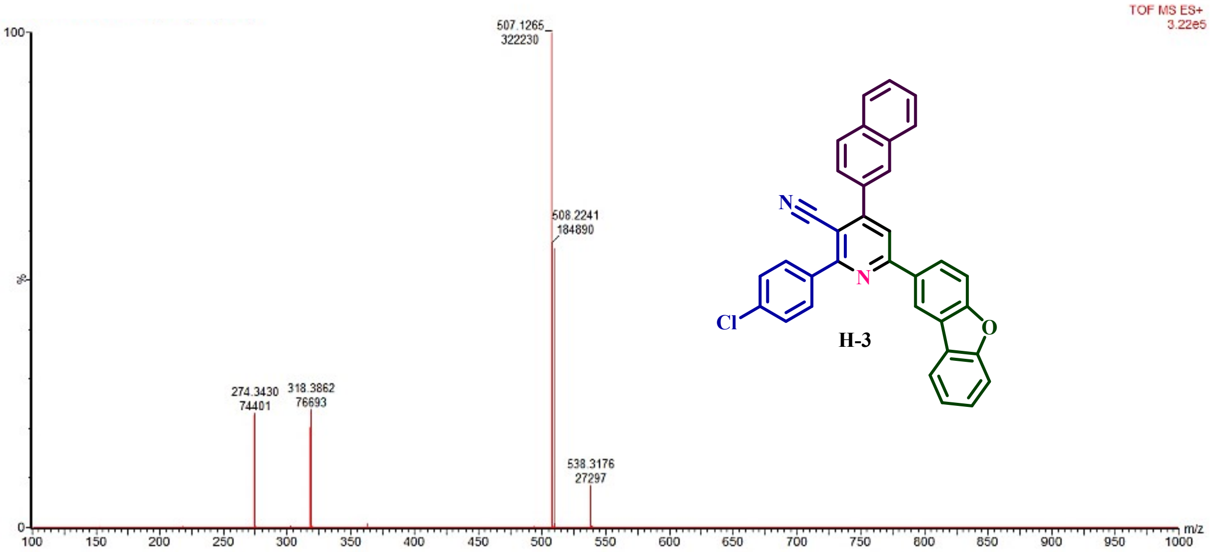

HR-Mass spectrum of 2-(4-chlorophenyl)-6-(dibenzo[*b*,*d*]furan-2-yl)-4-(naphthalen-2-yl)nicotinonitrile (H-3)

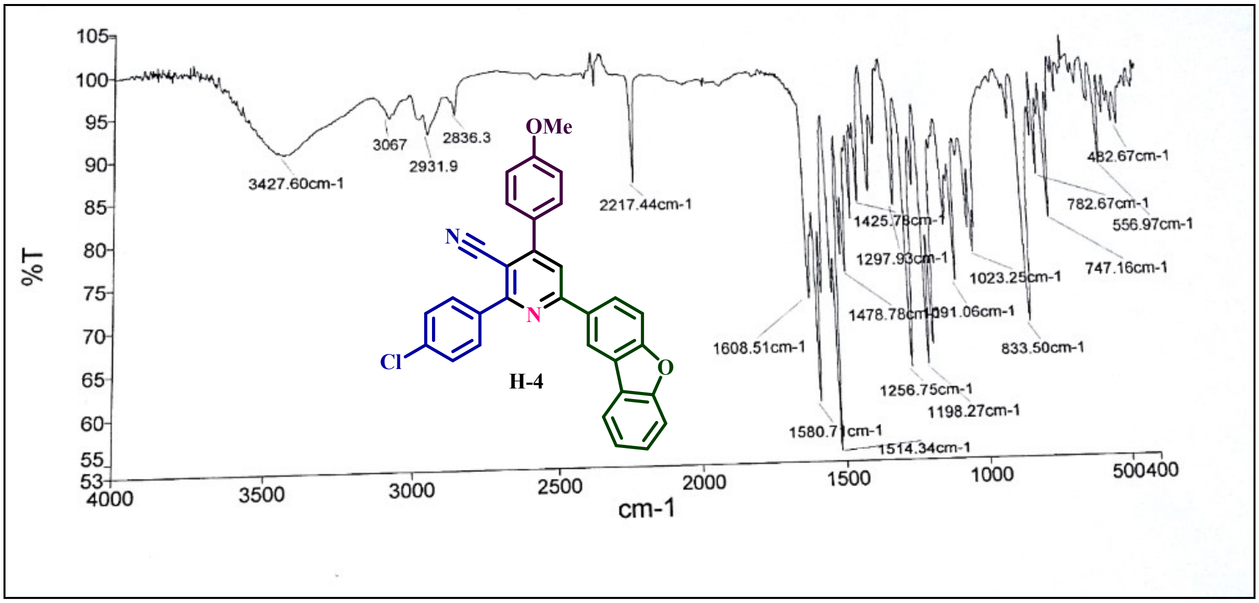

FT-IR spectrum of 2-(4-chlorophenyl)-6-(dibenzo[*b*,*d*]furan-2-yl)-4-(4-methoxyphenyl)nicotinonitrile (H-4)

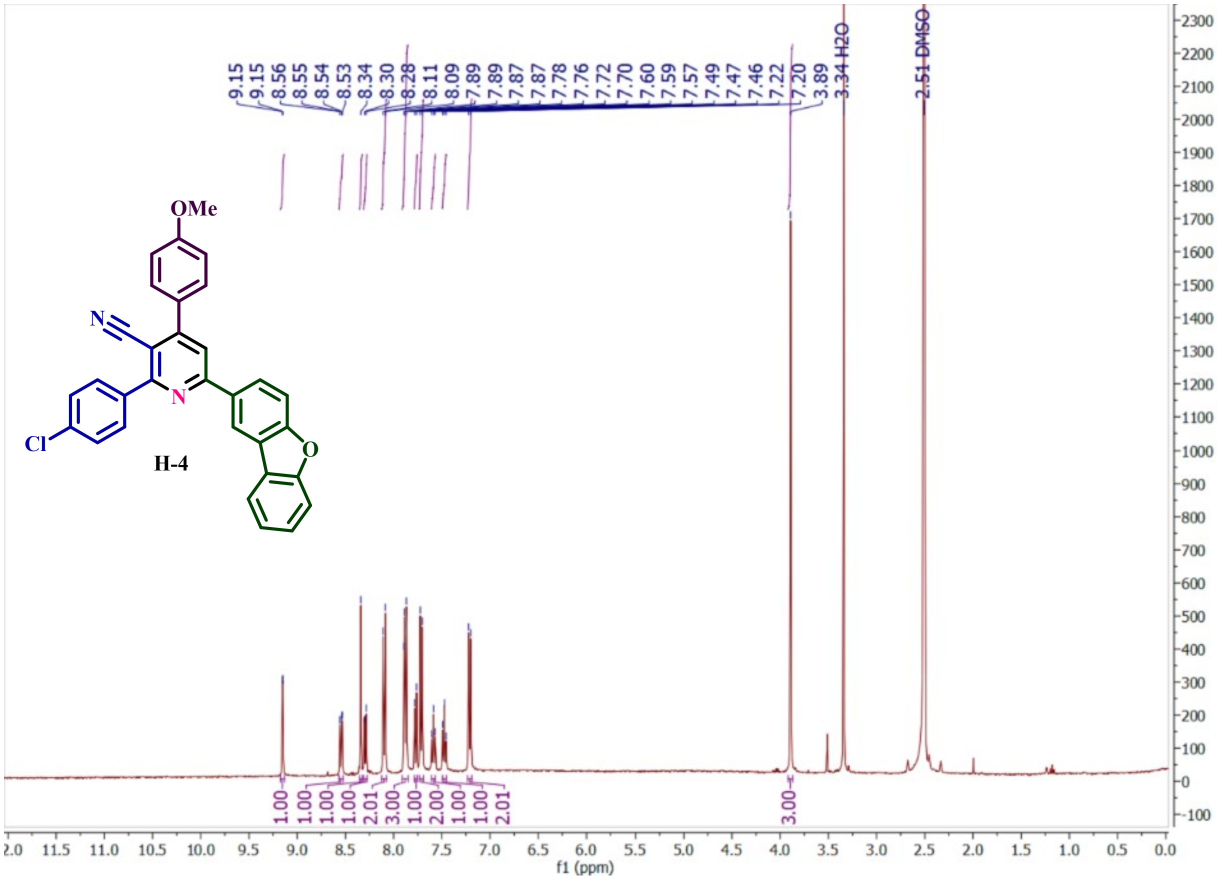

^{1}H-NMR spectrum of 2-(4-chlorophenyl)-6-(dibenzo[*b*,*d*]furan-2-yl)-4-(4-methoxyphenyl)nicotinonitrile (H-4)

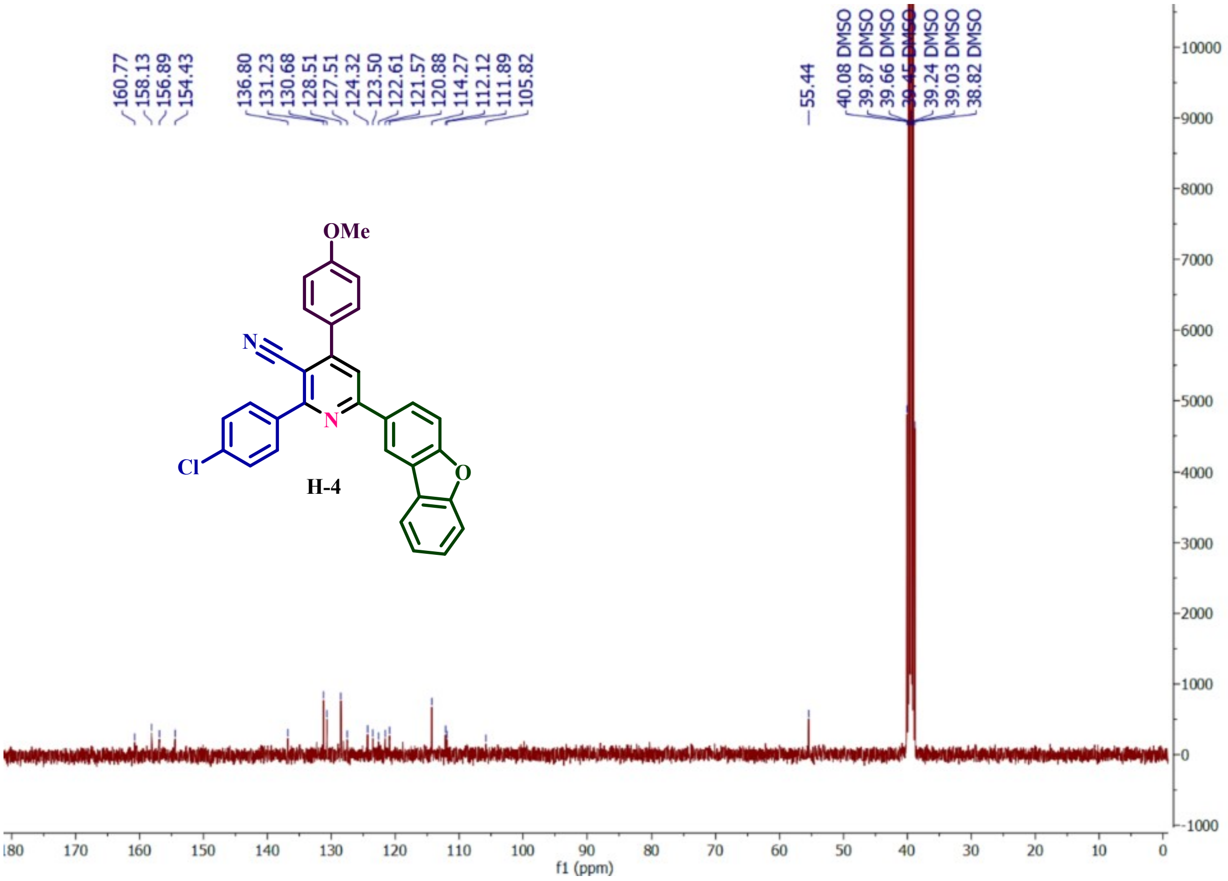

^{13}C-NMR spectrum of 2-(4-chlorophenyl)-6-(dibenzo[*b*,*d*]furan-2-yl)-4-(4-methoxyphenyl)nicotinonitrile (H-4)

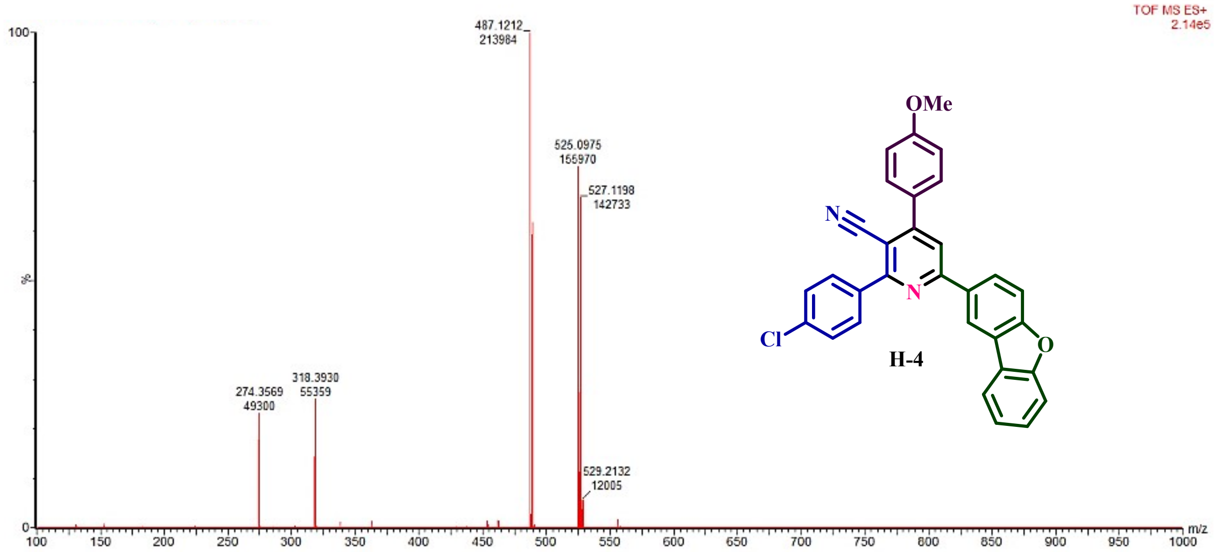

HR-Mass spectrum of 2-(4-chlorophenyl)-6-(dibenzo[*b*,*d*]furan-2-yl)-4-(4-methoxyphenyl)nicotinonitrile (H-4)

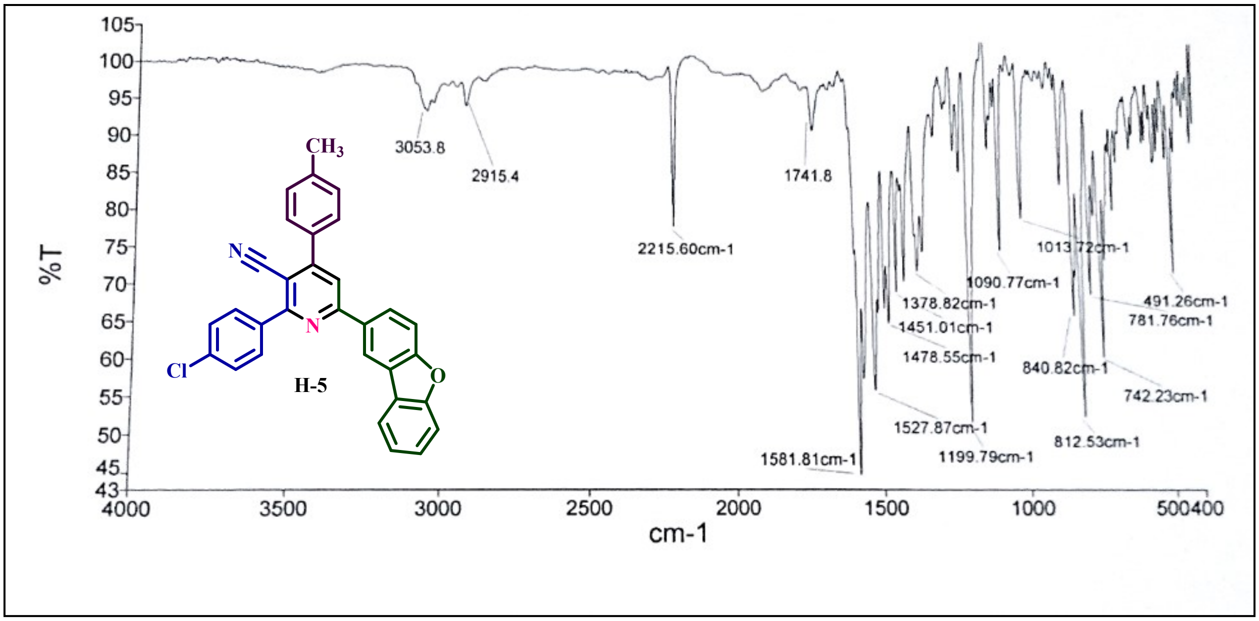

FT-IR spectrum of 2-(4-chlorophenyl)-6-(dibenzo[*b*,*d*]furan-2-yl)-4-(p-tolyl)nicotinonitrile (H-5)

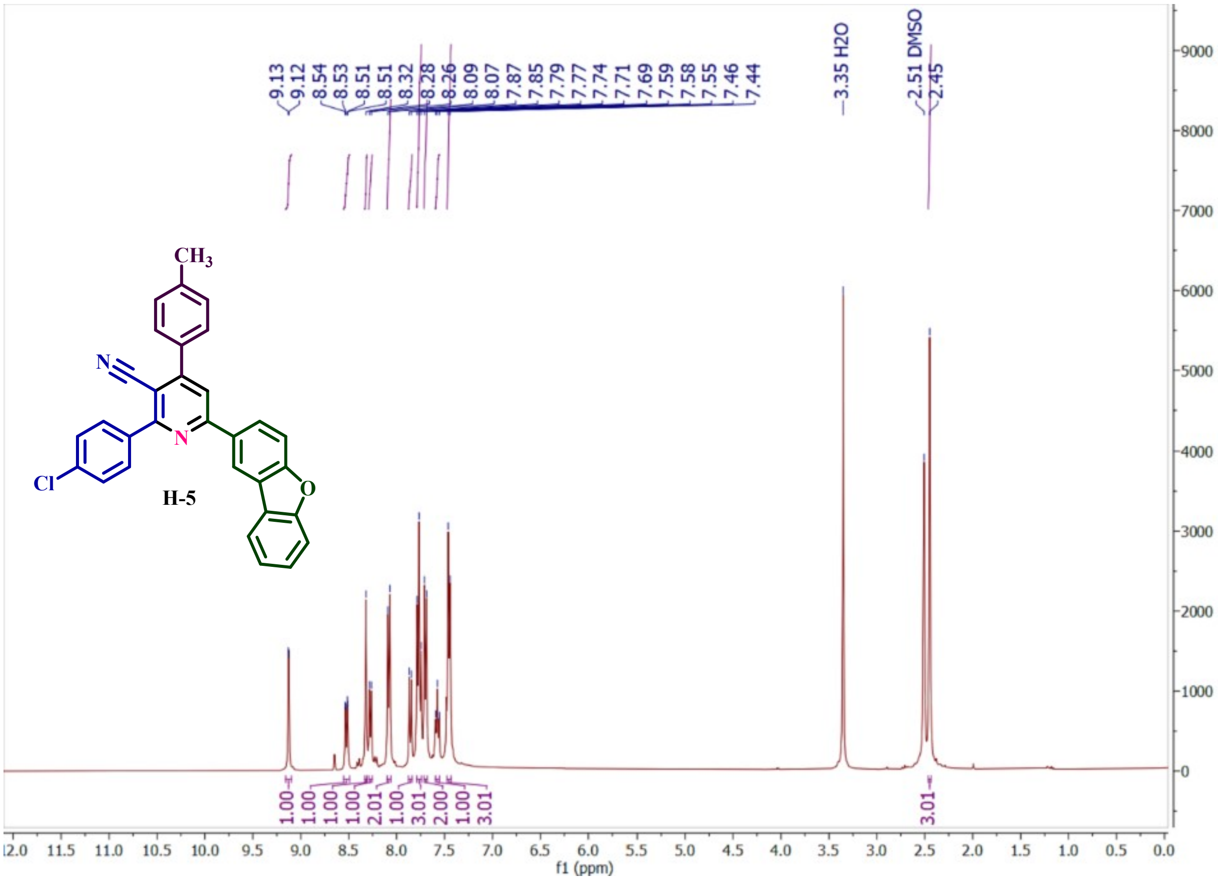

^{1}H-NMR spectrum of 2-(4-chlorophenyl)-6-(dibenzo[*b*,*d*]furan-2-yl)-4-(p-tolyl)nicotinonitrile (H-5)

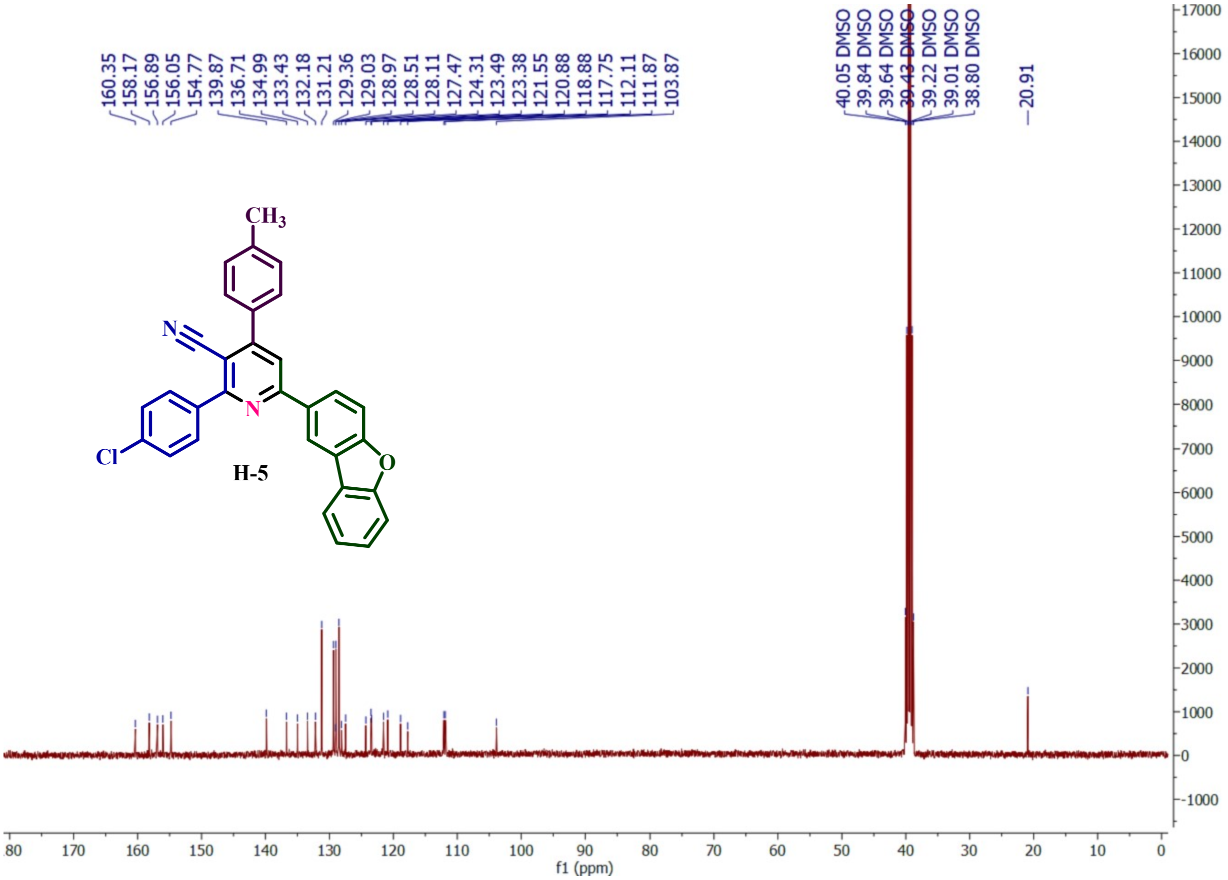

^{13}C-NMR spectrum of 2-(4-chlorophenyl)-6-(dibenzo[*b*,*d*]furan-2-yl)-4-(p-tolyl)nicotinonitrile (H-5)

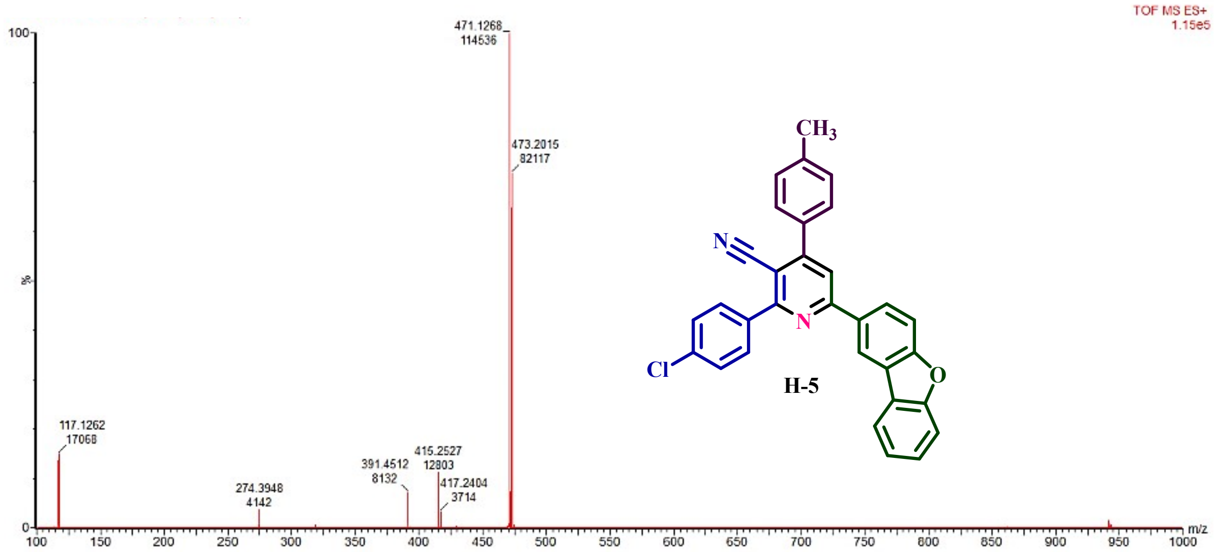

HR-Mass spectrum of 2-(4-chlorophenyl)-6-(dibenzo[*b*,*d*]furan-2-yl)-4-(p-tolyl)nicotinonitrile (H-5)

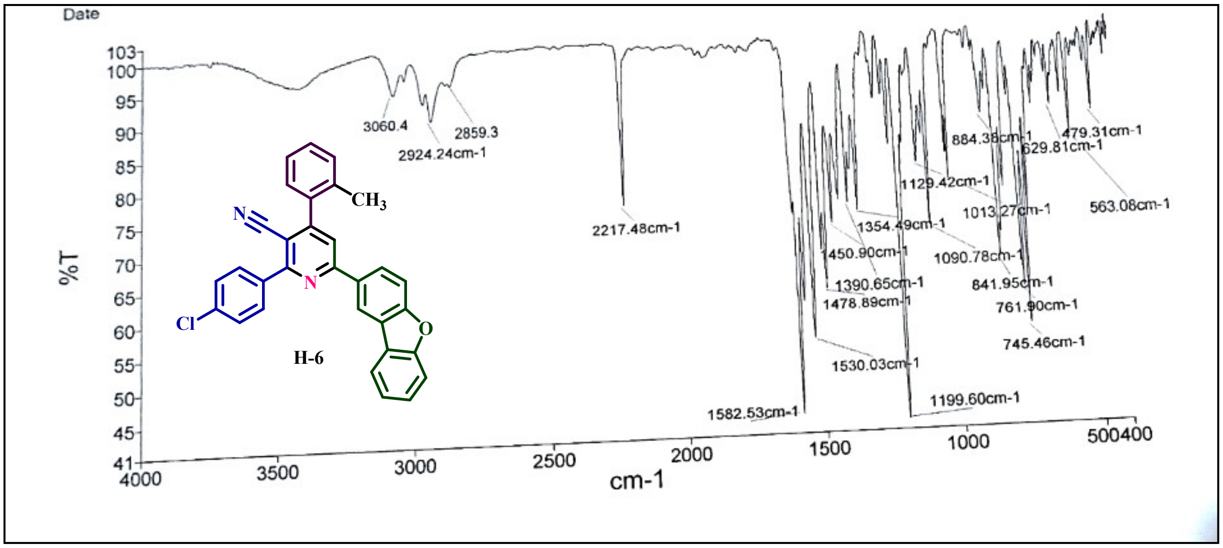

FT-IR spectrum of 2-(4-chlorophenyl)-6-(dibenzo[*b*,*d*]furan-2-yl)-4-(o-tolyl)nicotinonitrile (H-6)

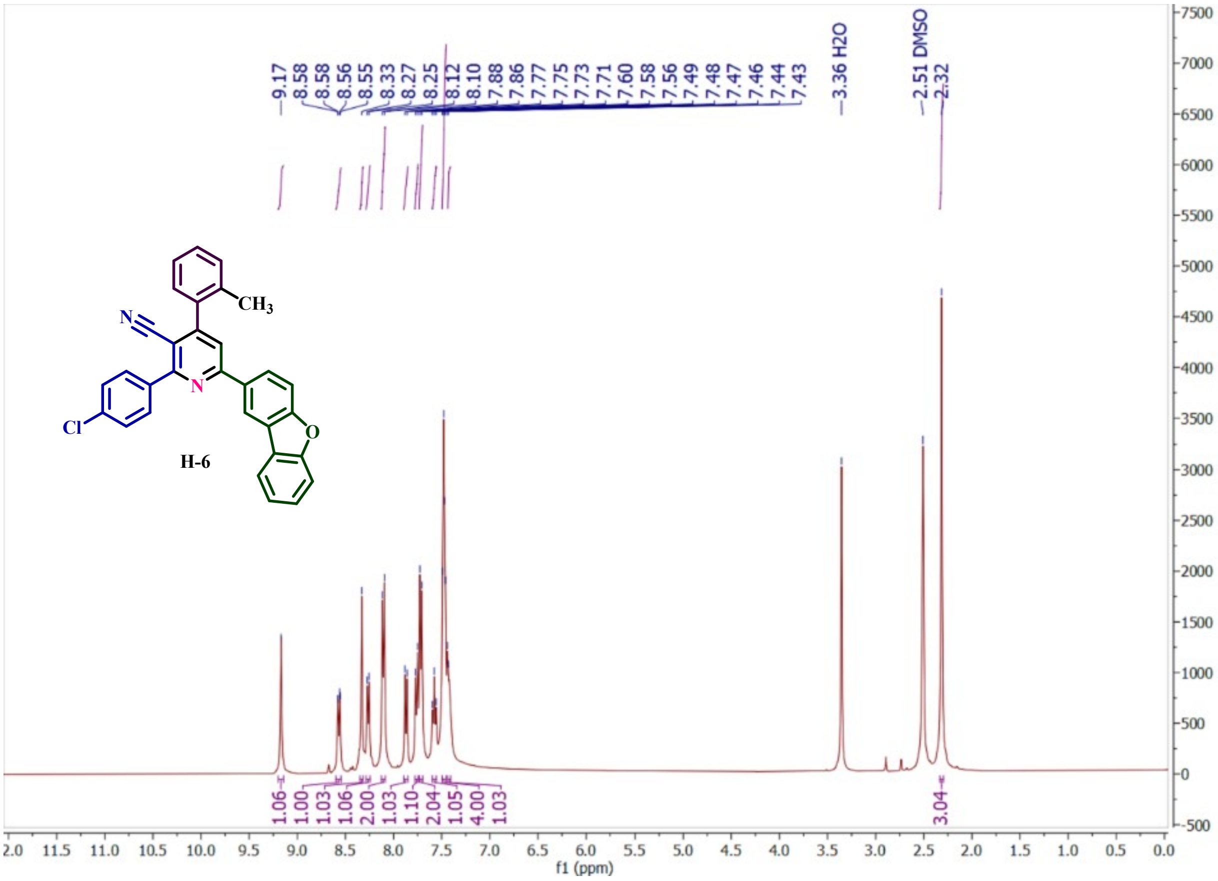

^{1}H-NMR spectrum of 2-(4-chlorophenyl)-6-(dibenzo[*b*,*d*]furan-2-yl)-4-(o-tolyl)nicotinonitrile (H-6)

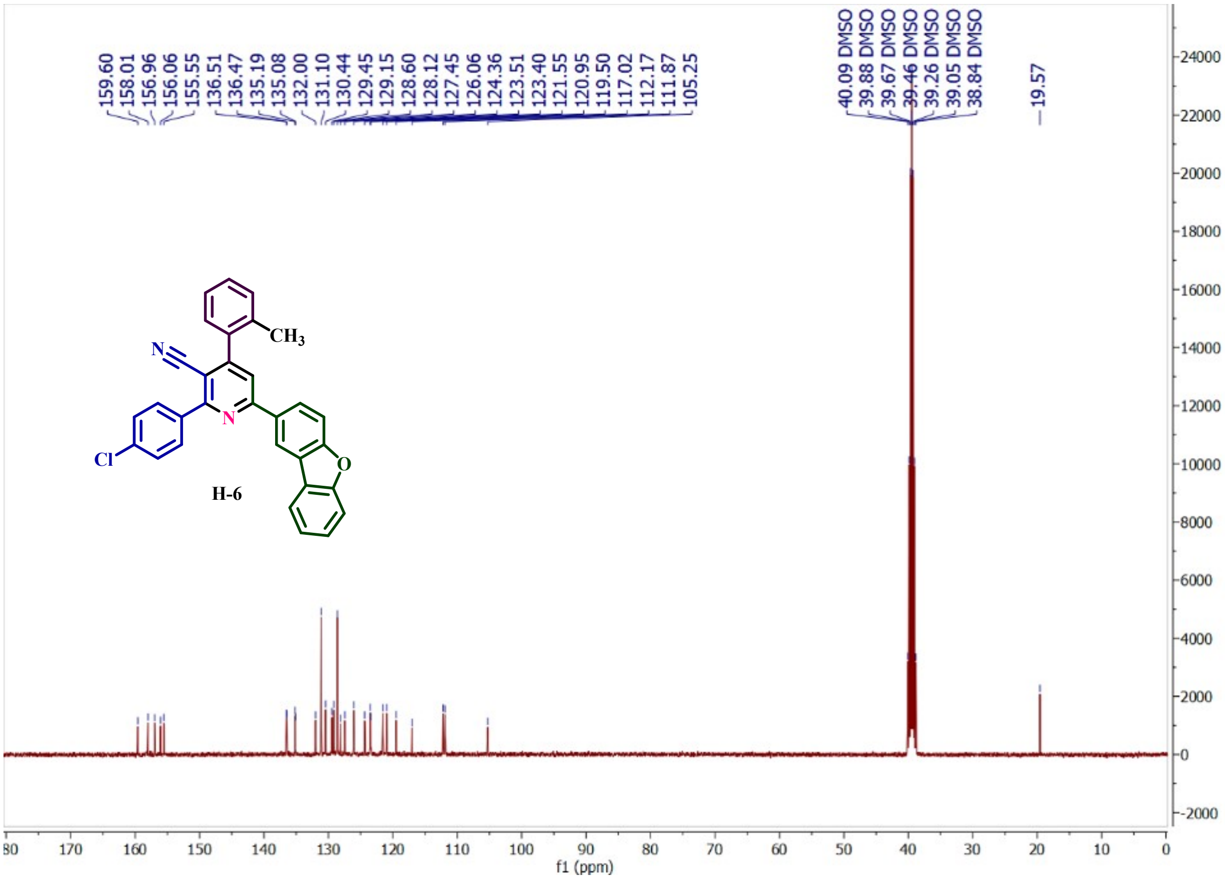

^{13}C-NMR spectrum of 2-(4-chlorophenyl)-6-(dibenzo[*b*,*d*]furan-2-yl)-4-(o-tolyl)nicotinonitrile (H-6)

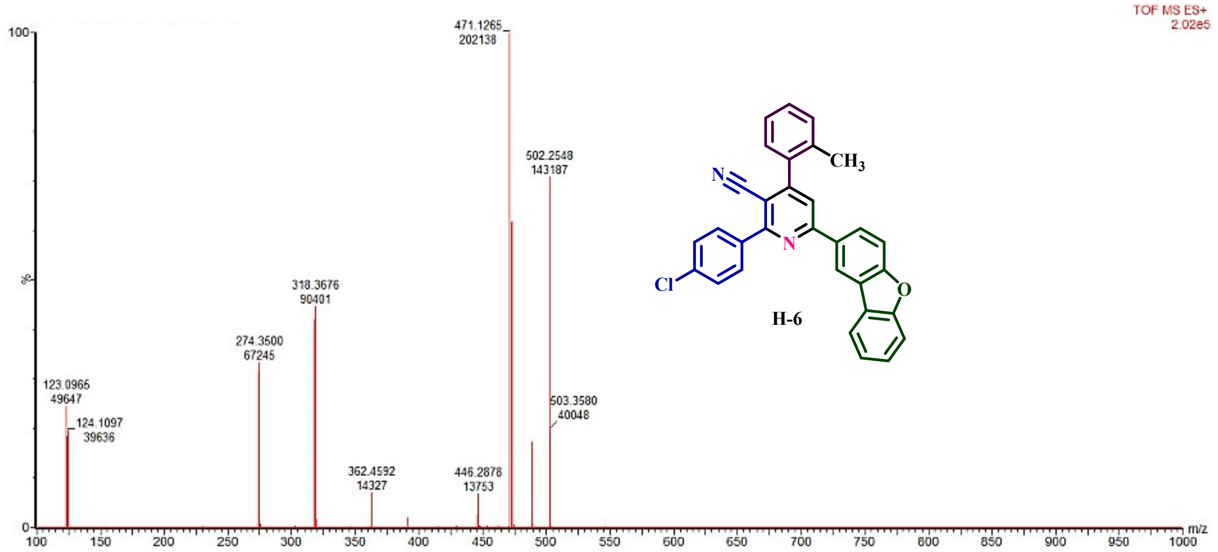

HR-Mass spectrum of 2-(4-chlorophenyl)-6-(dibenzo[*b*,*d*]furan-2-yl)-4-(o-tolyl)nicotinonitrile (H-6)

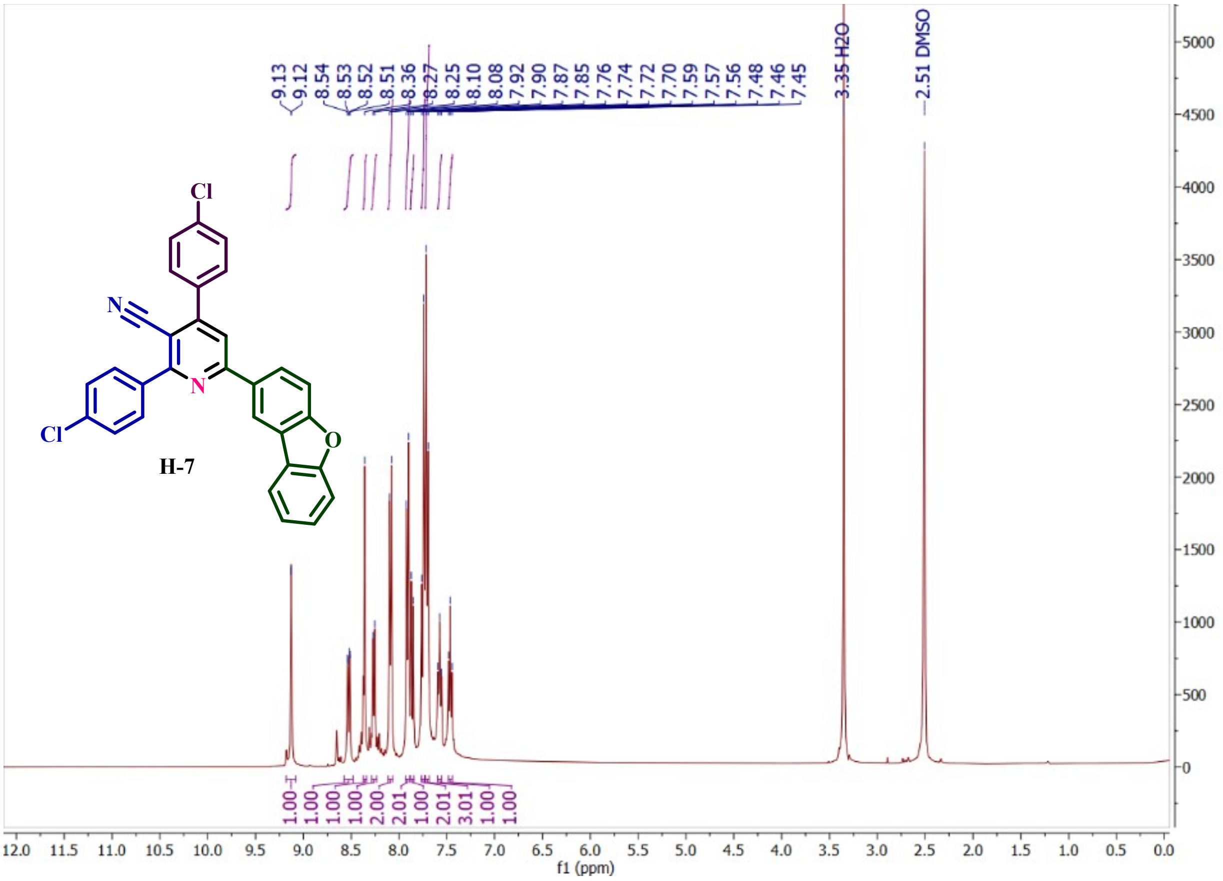

^{1}H-NMR spectrum of 2,4-bis(4-chlorophenyl)-6-(dibenzo[*b*,*d*]furan-2-yl)nicotinonitrile (H-7)

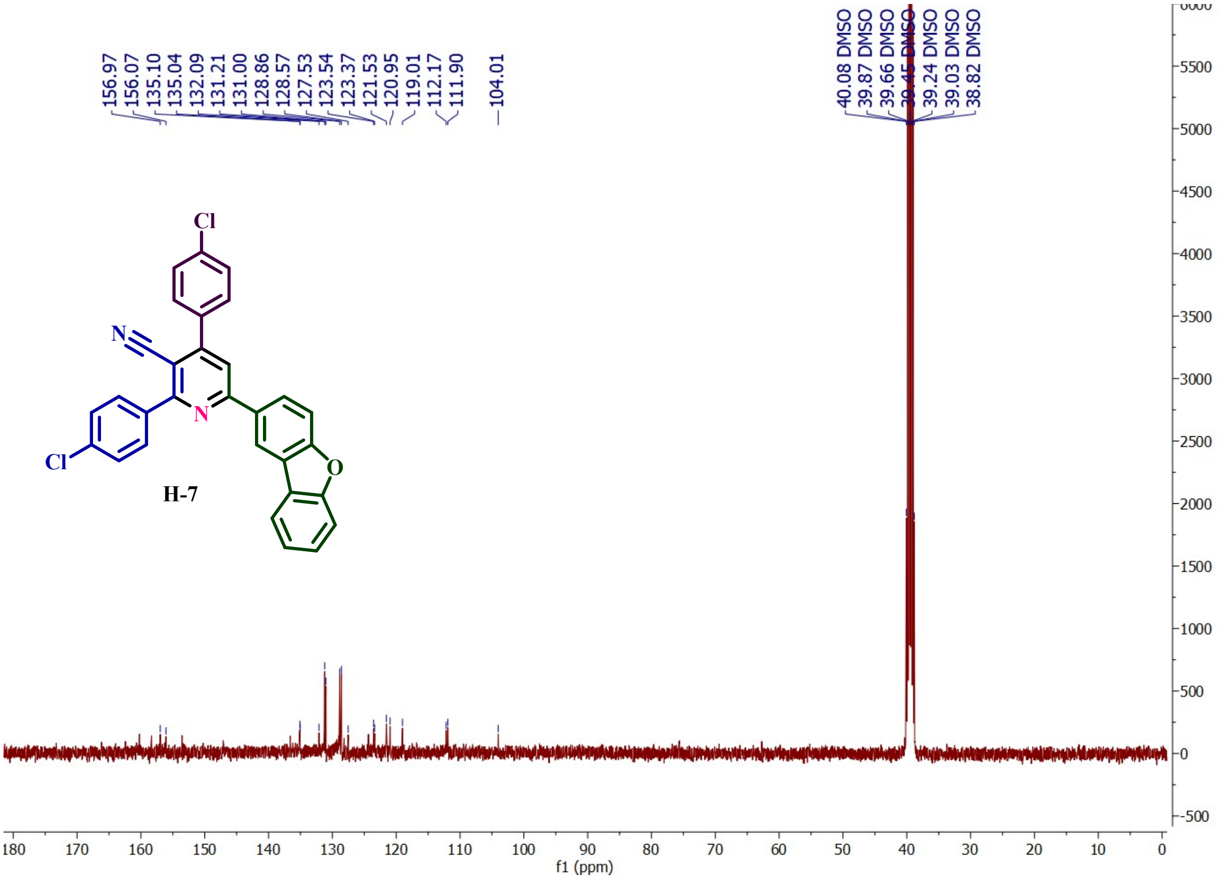

^{13}C-NMR spectrum of 2,4-bis(4-chlorophenyl)-6-(dibenzo[*b*,*d*]furan-2-yl)nicotinonitrile (H-7)

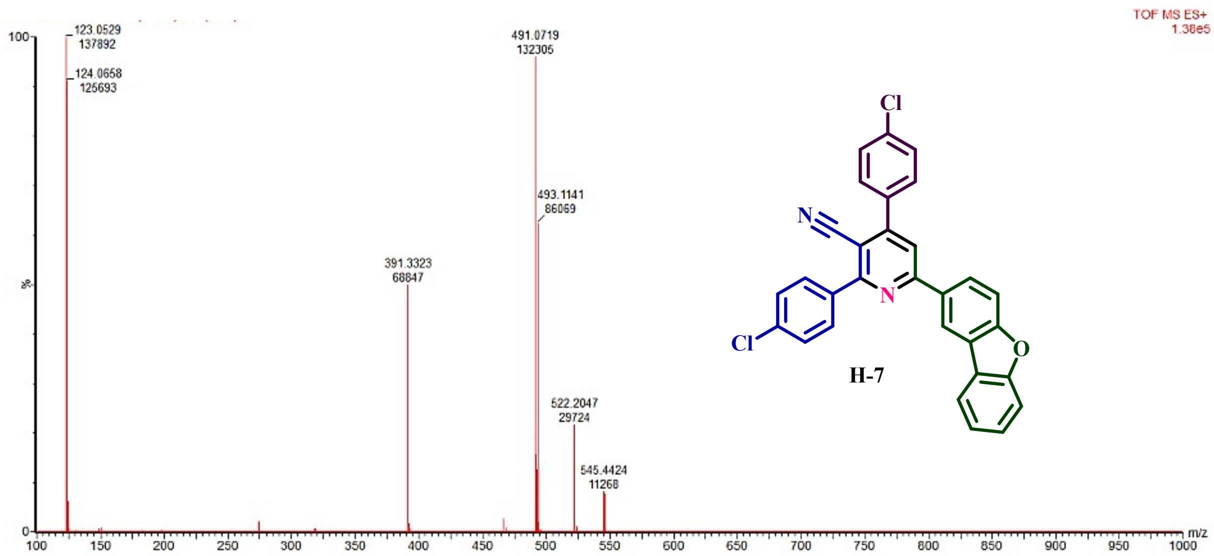

HR-Mass spectrum of 2,4-bis(4-chlorophenyl)-6-(dibenzo[*b*,*d*]furan-2-yl)nicotinonitrile (H-7)

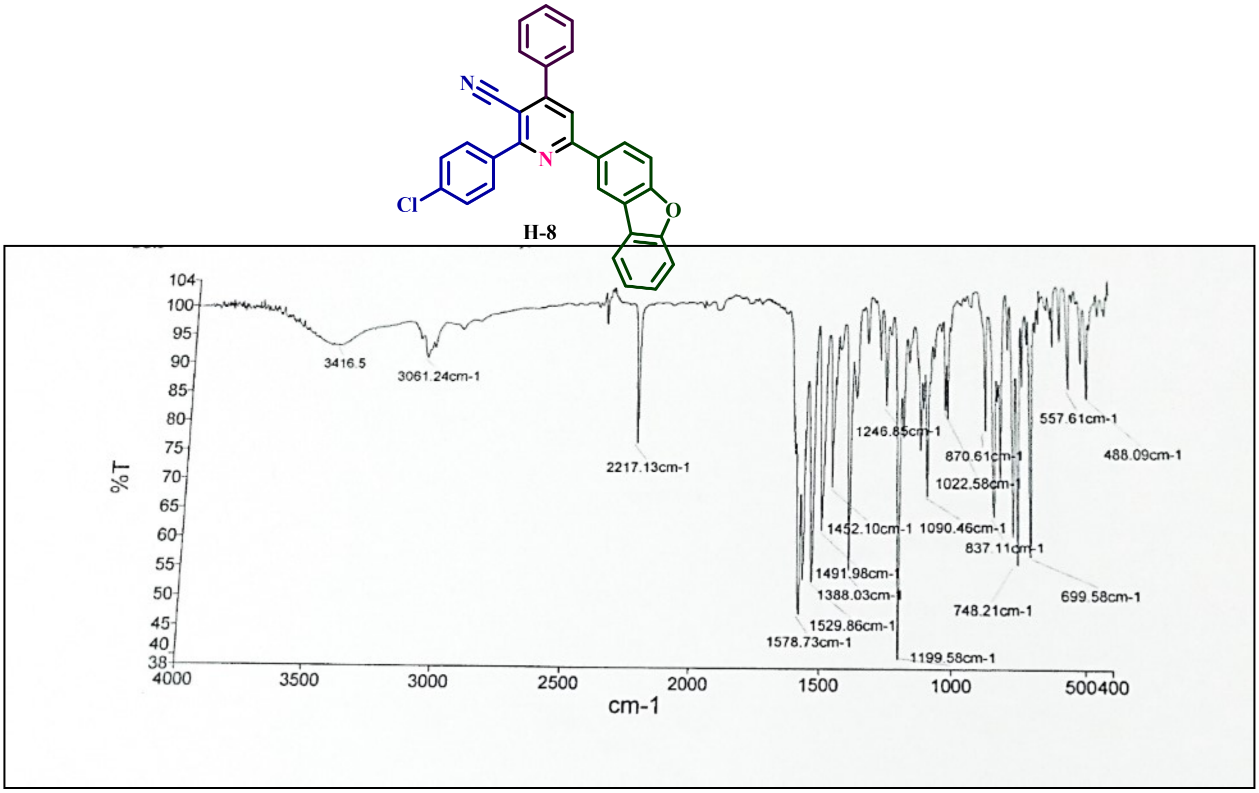

FT-IR spectrum of 2-(4-chlorophenyl)-6-(dibenzo[*b*,*d*]furan-2-yl)-4-phenylnicotinonitrile (H-8)

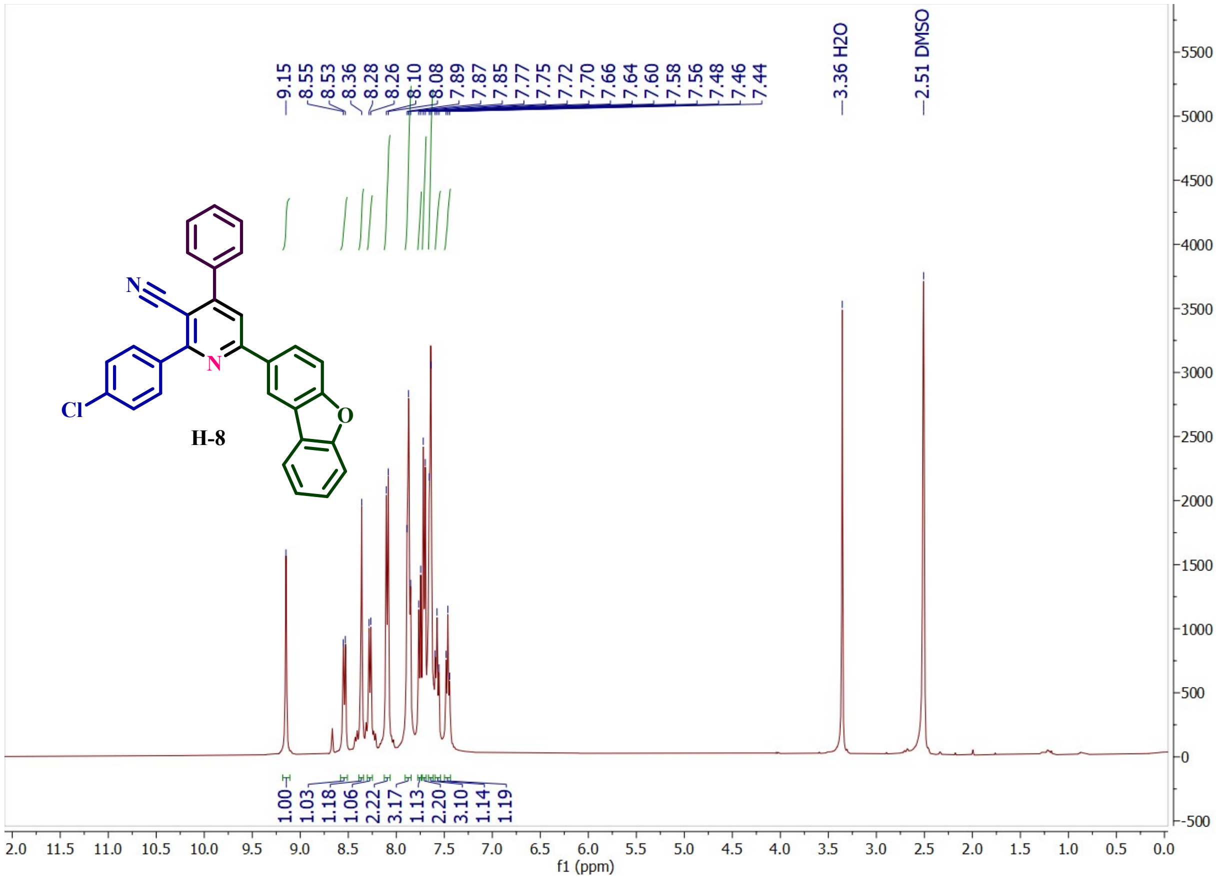

^{1}H-NMR spectrum of 2-(4-chlorophenyl)-6-(dibenzo[*b*,*d*]furan-2-yl)-4-phenylnicotinonitrile (H-8)

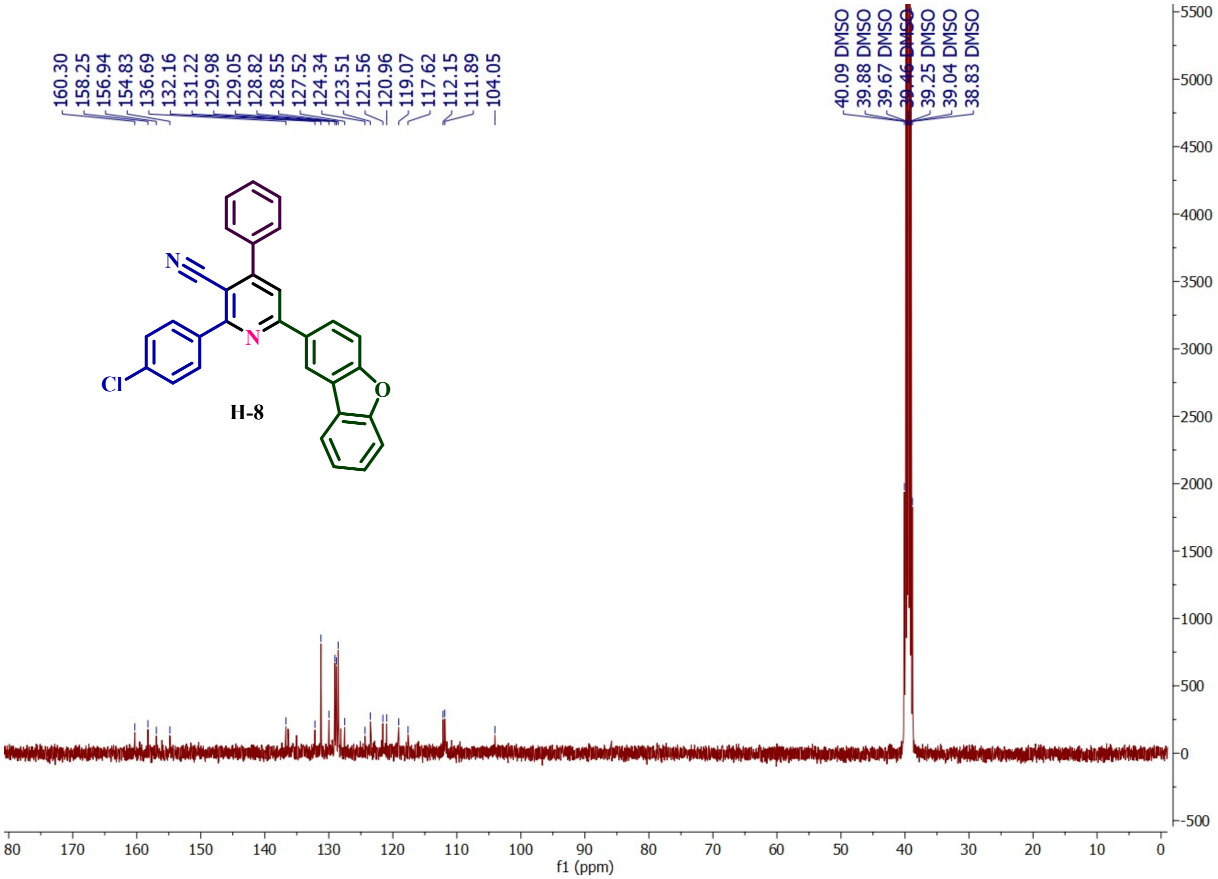

^{13}C-NMR spectrum of 2-(4-chlorophenyl)-6-(dibenzo[*b*,*d*]furan-2-yl)-4-phenylnicotinonitrile (H-8)

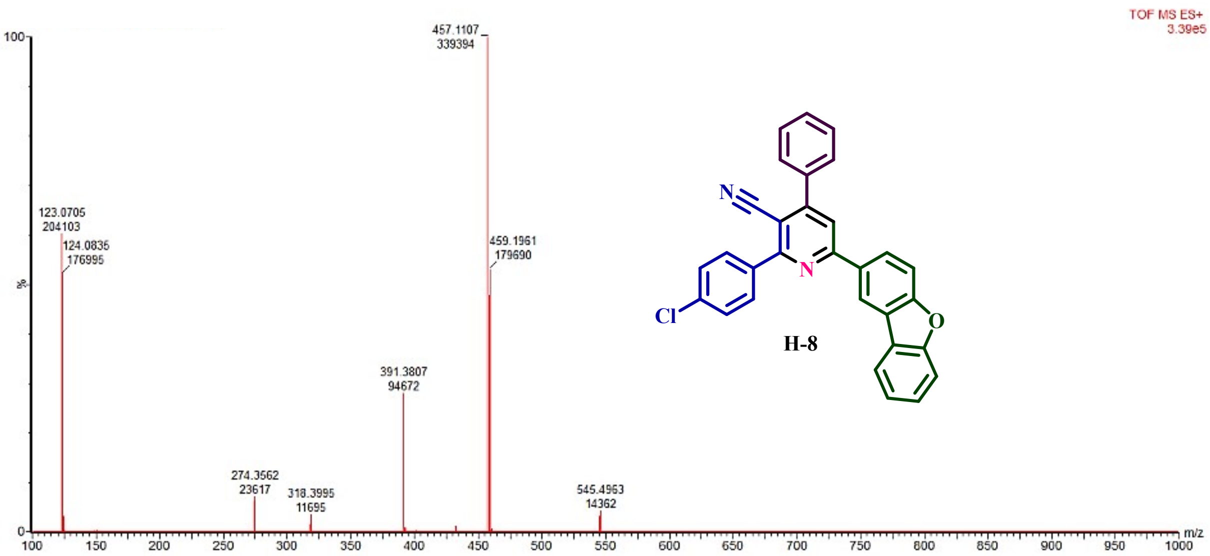

HR-Mass spectrum of 2-(4-chlorophenyl)-6-(dibenzo[*b*,*d*]furan-2-yl)-4-phenylnicotinonitrile (H-8)

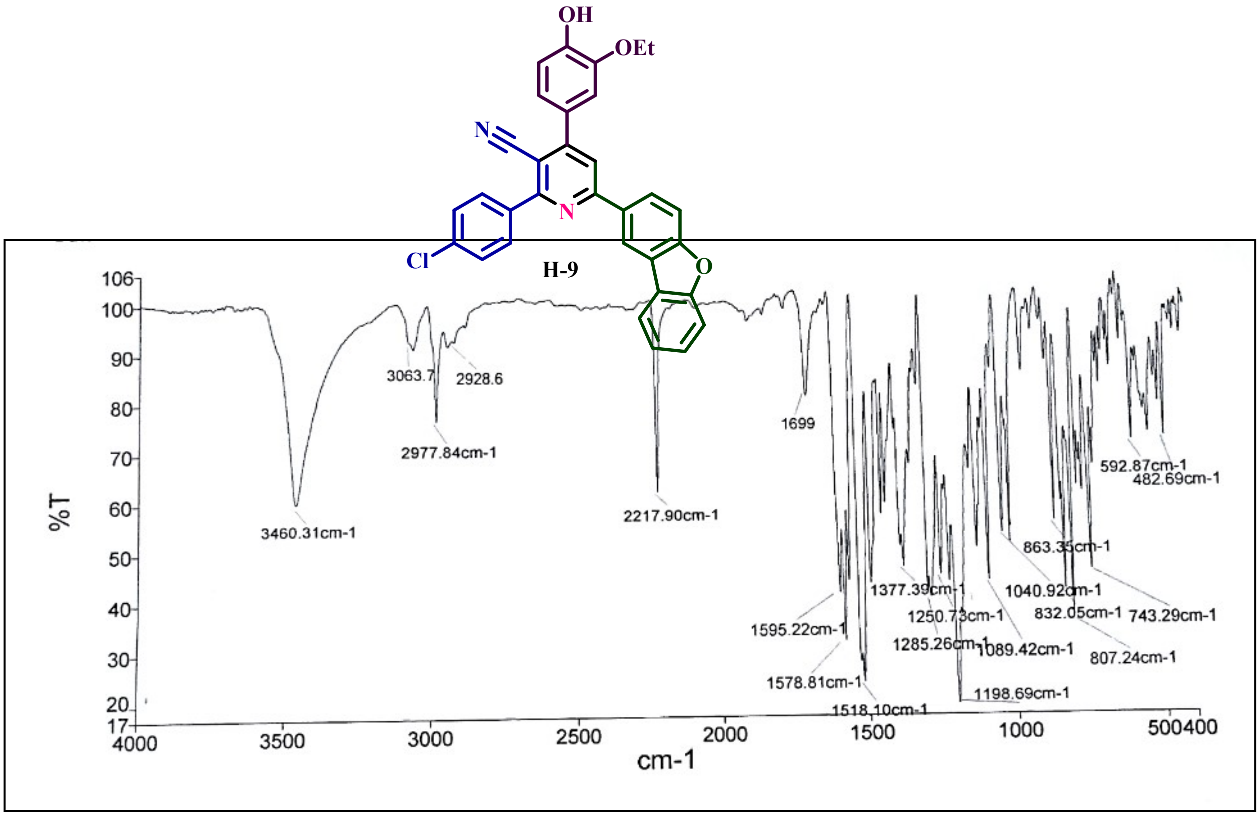

FT-IR spectrum of 2-(4-chlorophenyl)-6-(dibenzo[*b*,*d*]furan-2-yl)-4-(4-hydroxy-3-ethoxyphenyl)nicotinonitrile (H-9)

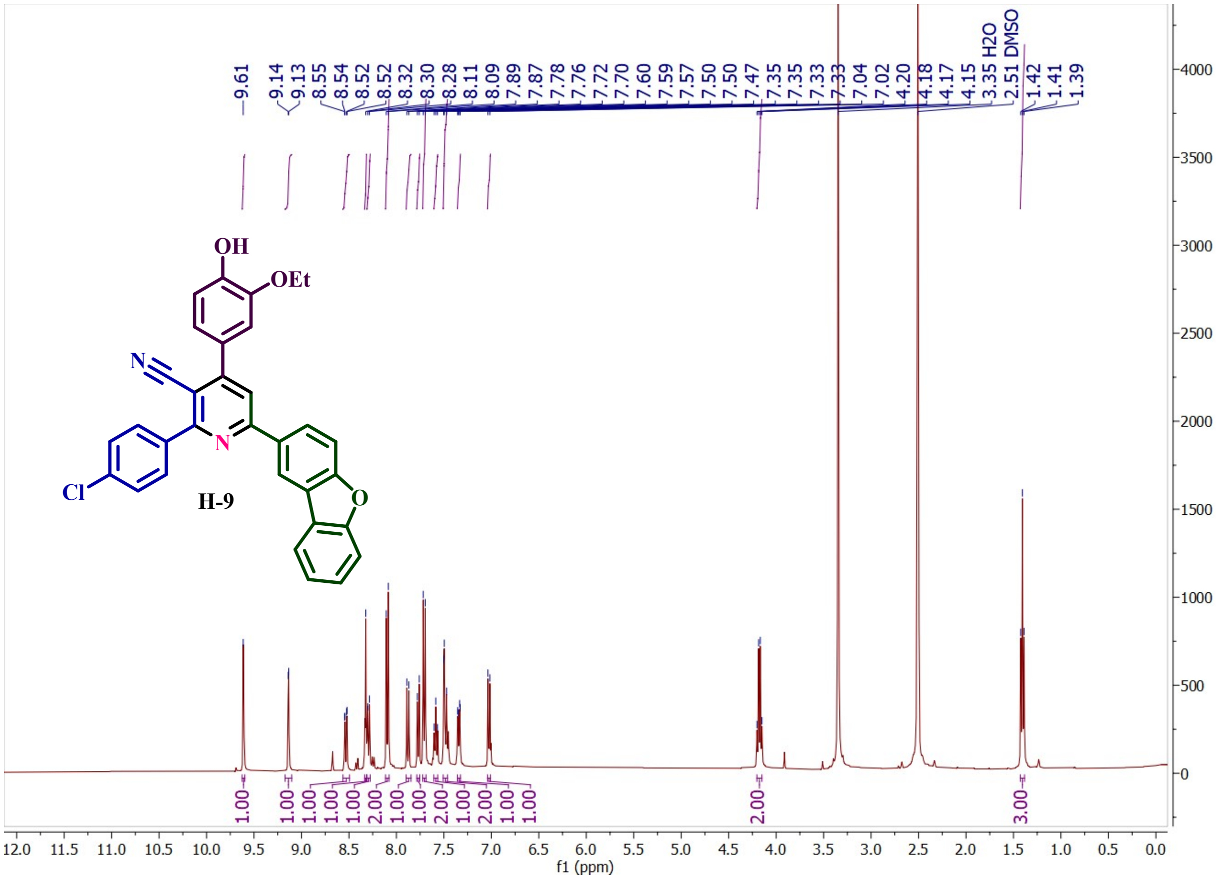

^{1}H-NMR spectrum of 2-(4-chlorophenyl)-6-(dibenzo[*b*,*d*]furan-2-yl)-4-(4-hydroxy-3-ethoxyphenyl)nicotinonitrile (H-9)

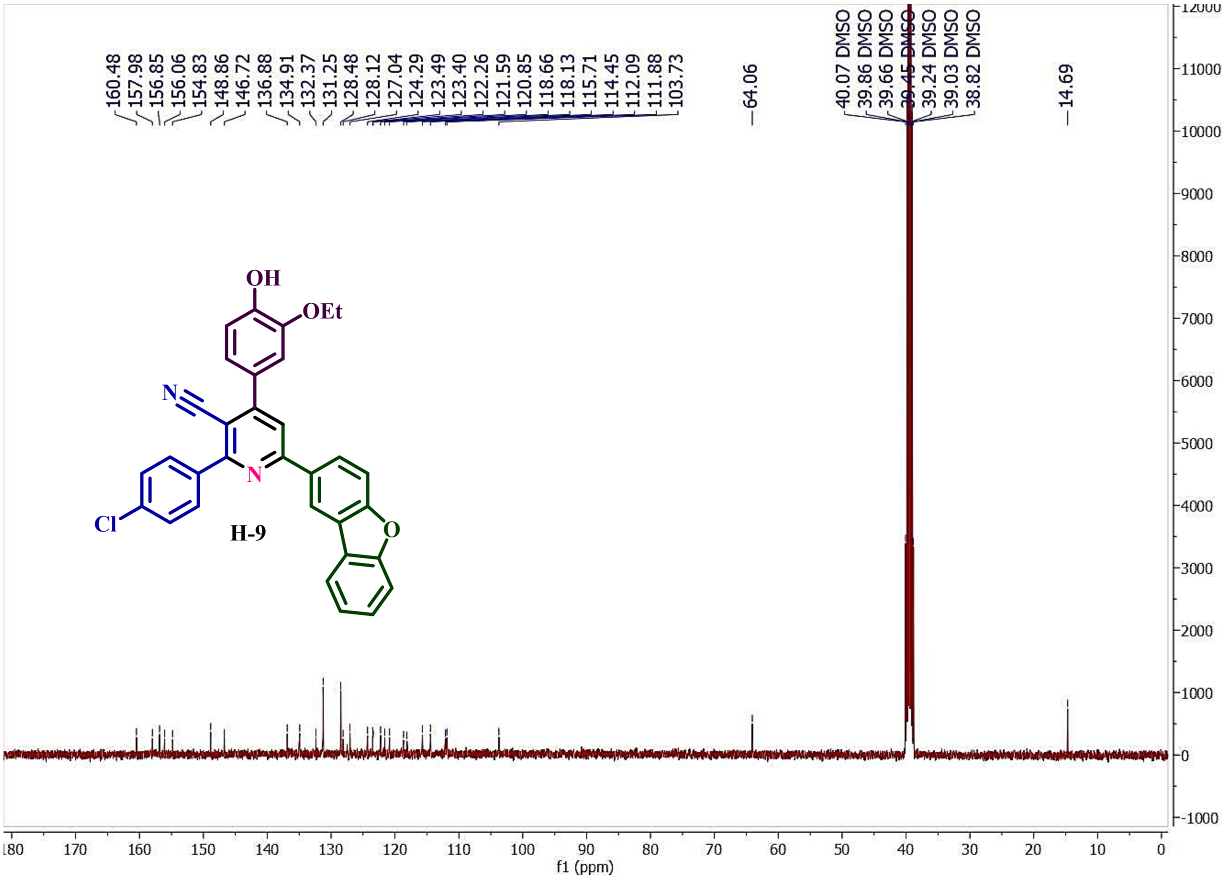

^{13}C-NMR spectrum of 2-(4-chlorophenyl)-6-(dibenzo[*b*,*d*]furan-2-yl)-4-(4-hydroxy-3-ethoxyphenyl)nicotinonitrile (H-9)

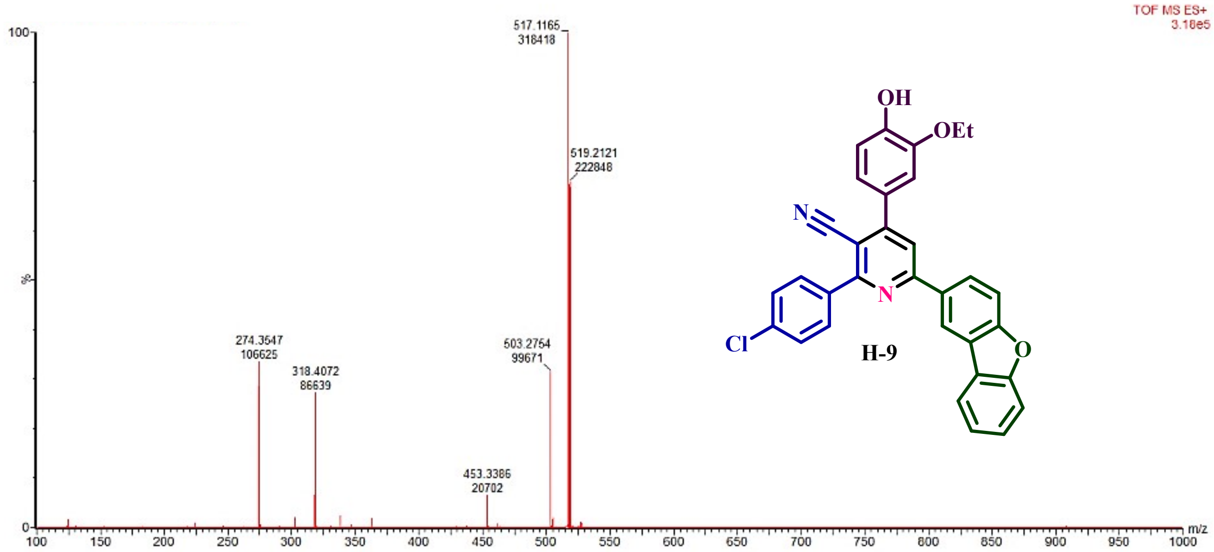

HR-Mass spectrum of 2-(4-chlorophenyl)-6-(dibenzo[*b*,*d*]furan-2-yl)-4-(4-hydroxy-3-ethoxyphenyl)nicotinonitrile (H-9)

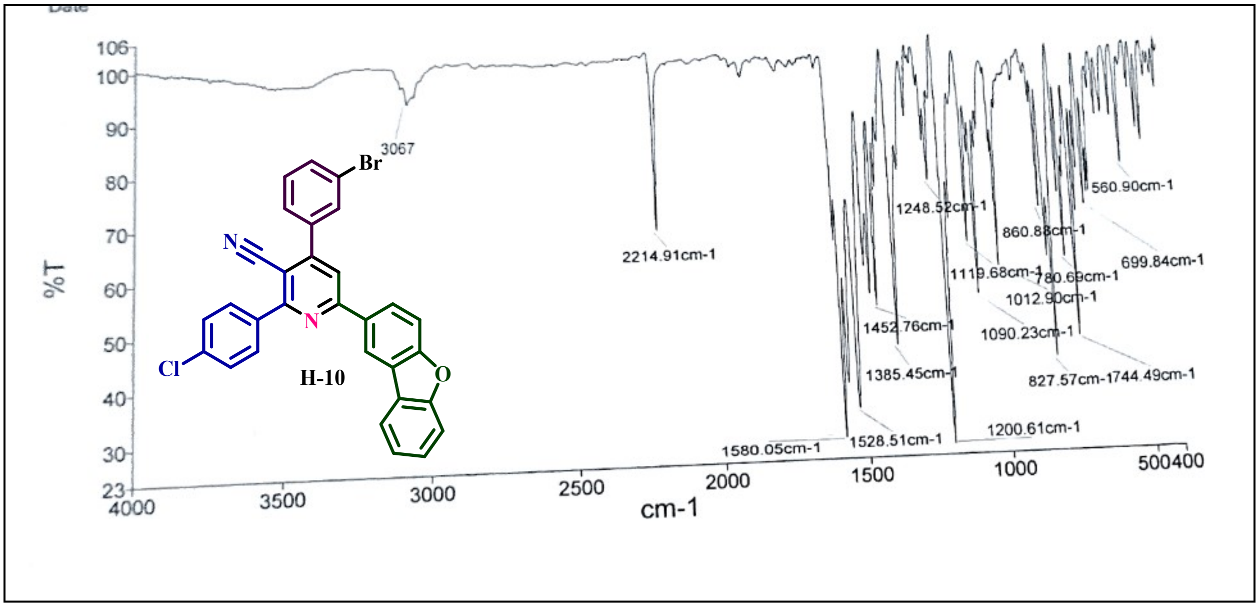

FT-IR spectrum of 4-(3-bromophenyl)-2-(4-chlorophenyl)-6-(dibenzo[*b*,*d*]furan-2-yl)nicotinonitrile (H-10)

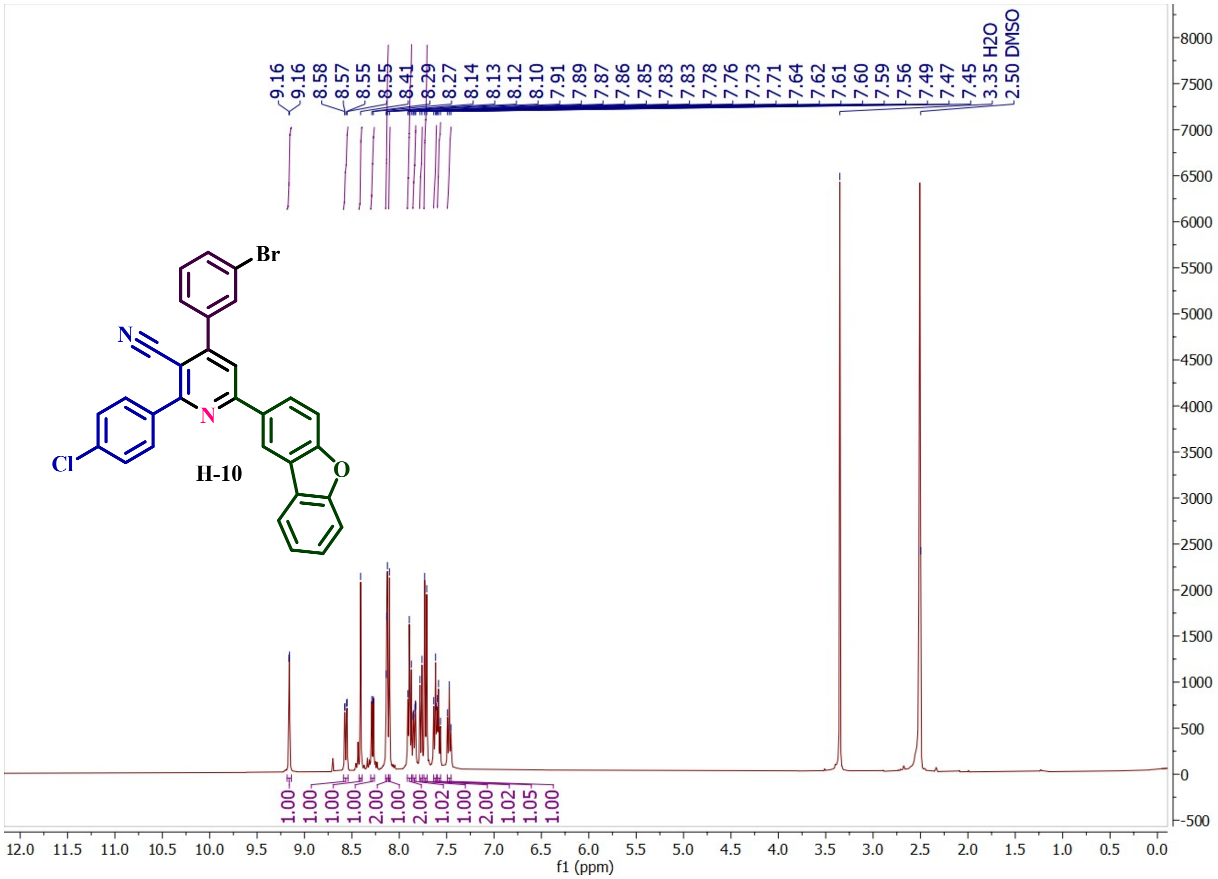

^{1}H-NMR spectrum of 4-(3-bromophenyl)-2-(4-chlorophenyl)-6-(dibenzo[*b*,*d*]furan-2-yl)nicotinonitrile (H-10)

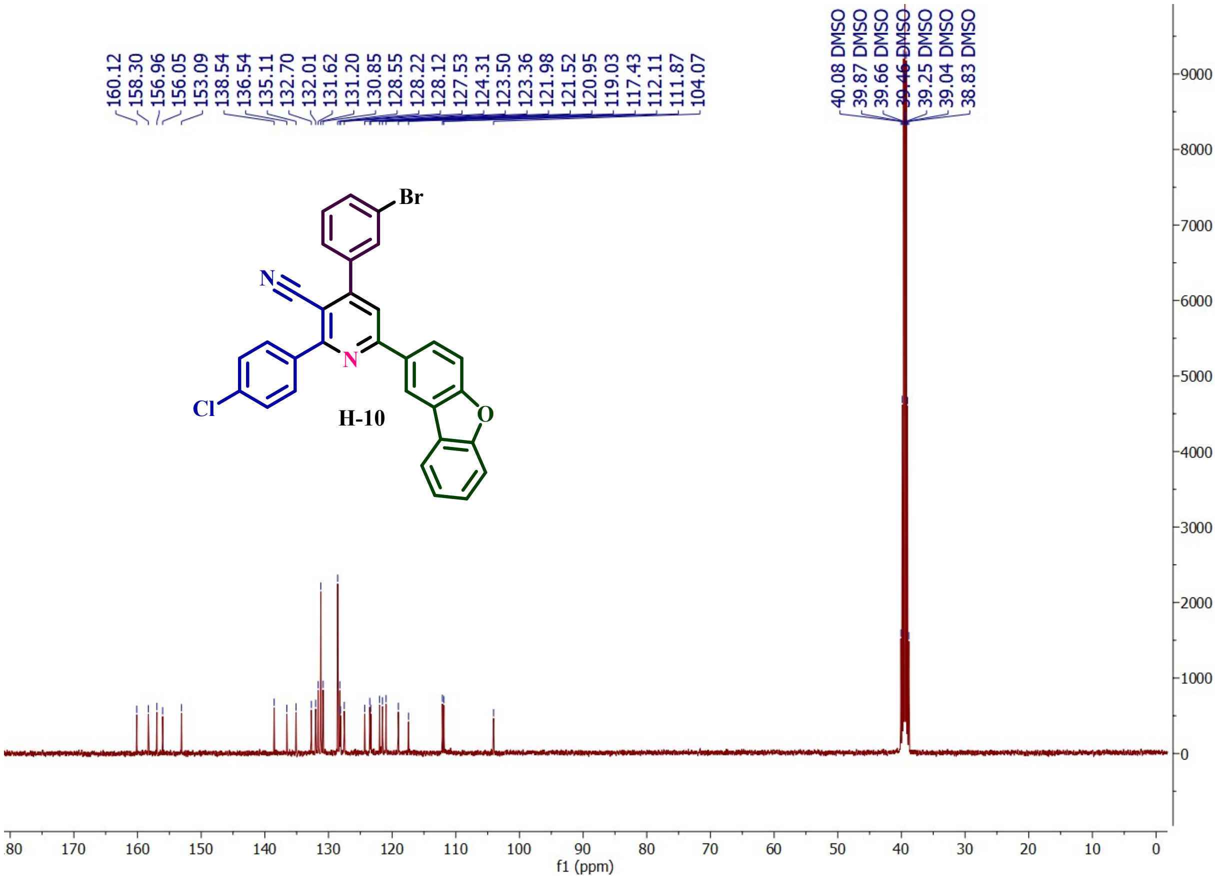

^{13}C-NMR spectrum of 4-(3-bromophenyl)-2-(4-chlorophenyl)-6-(dibenzo[*b*,*d*]furan-2-yl)nicotinonitrile (H-10)

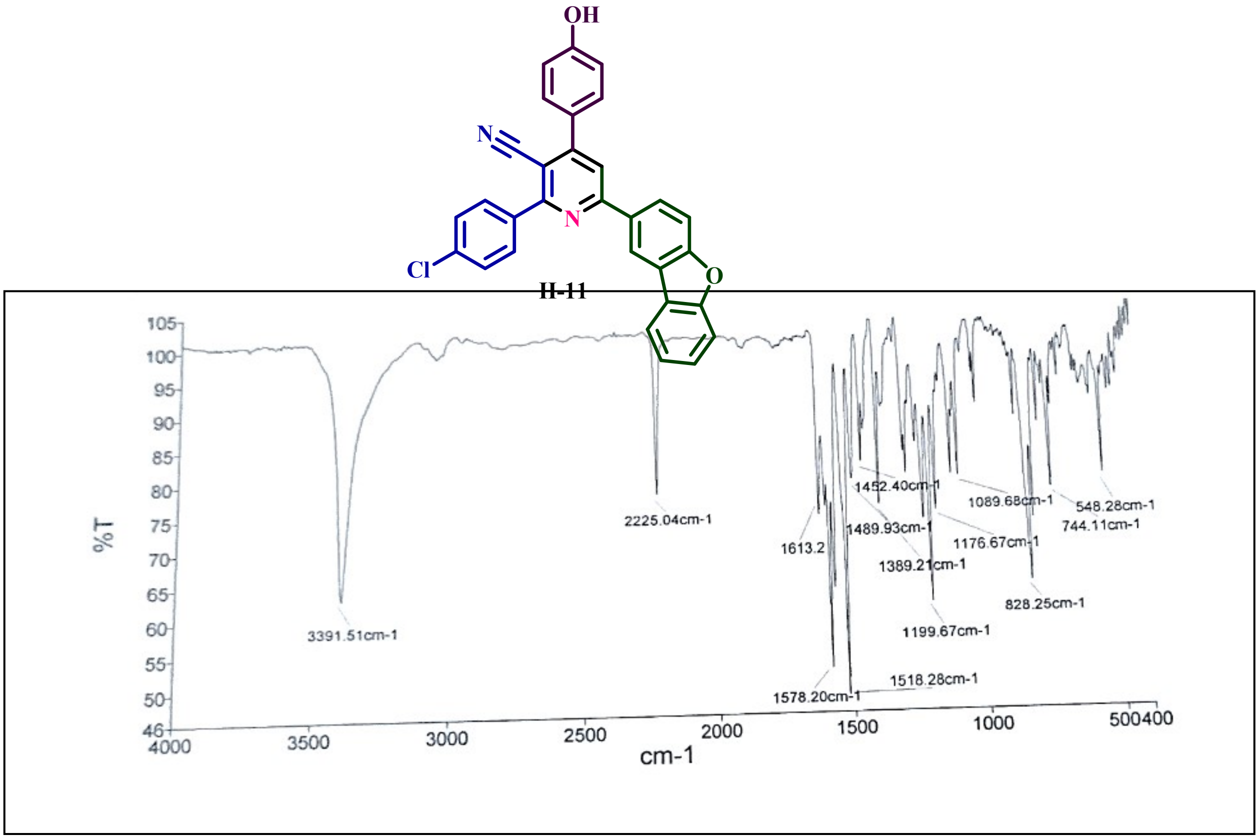

FT-IR spectrum of 2-(4-chlorophenyl)-6-(dibenzo[*b*,*d*]furan-2-yl)-4-(4-hydroxyphenyl)nicotinonitrile (H-11)

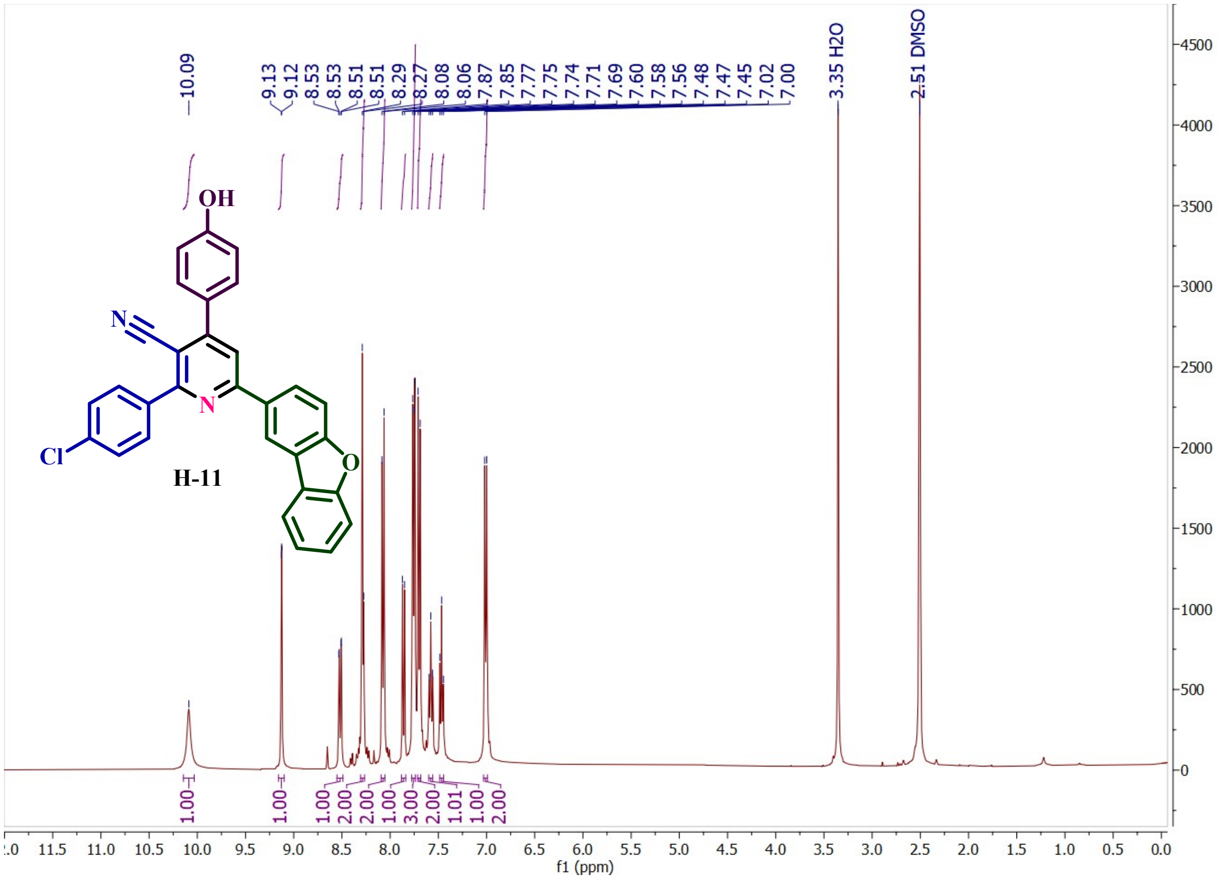

^{1}H-NMR spectrum of 2-(4-chlorophenyl)-6-(dibenzo[*b*,*d*]furan-2-yl)-4-(4-hydroxyphenyl)nicotinonitrile (H-11)

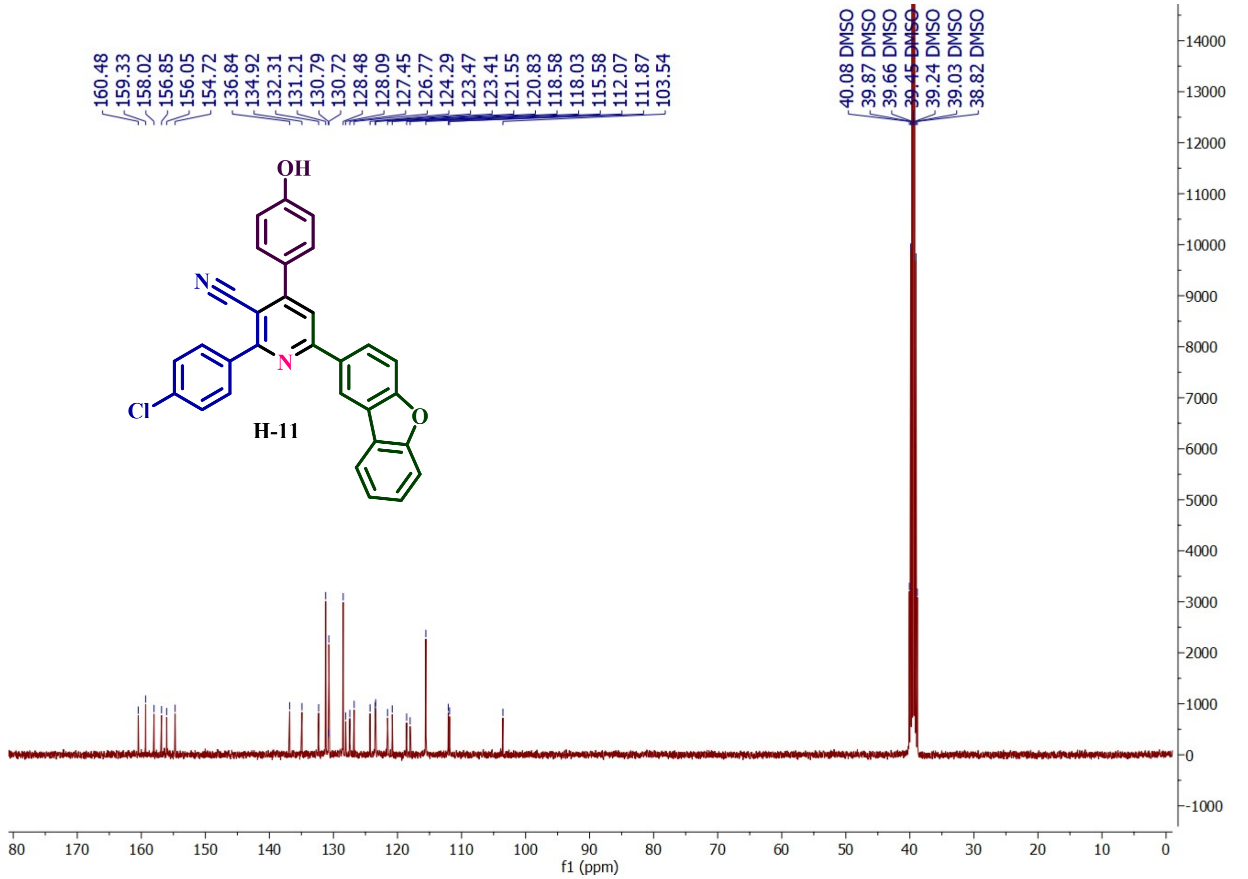

^{13}C-NMR spectrum of 2-(4-chlorophenyl)-6-(dibenzo[*b*,*d*]furan-2-yl)-4-(4-hydroxyphenyl)nicotinonitrile (H-11)

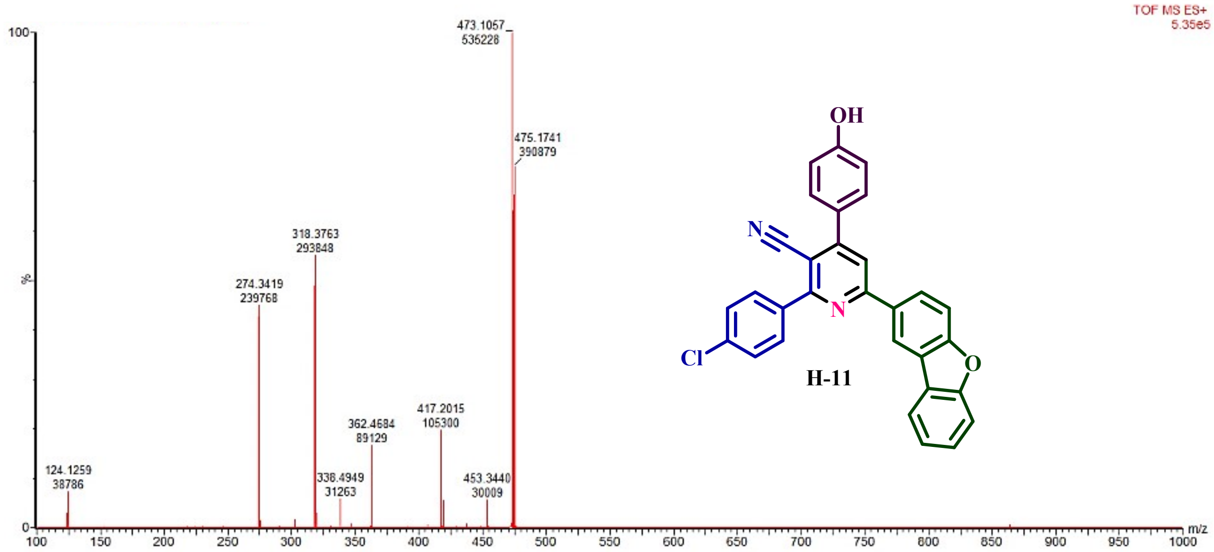

HR-Mass spectrum of 2-(4-chlorophenyl)-6-(dibenzo[*b*,*d*]furan-2-yl)-4-(4-hydroxyphenyl)nicotinonitrile (H-11)

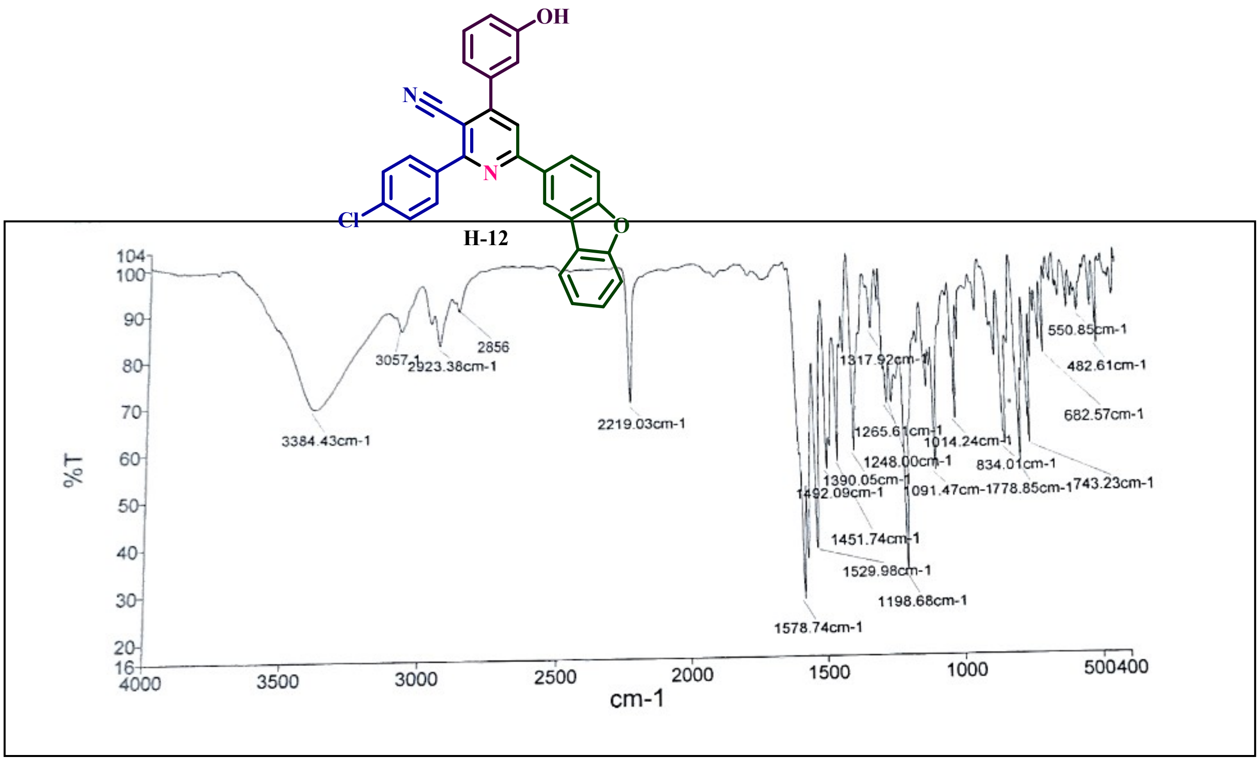

FT-IR spectrum of 2-(4-chlorophenyl)-6-(dibenzo[*b*,*d*]furan-2-yl)-4-(3-hydroxyphenyl)nicotinonitrile (H-12)

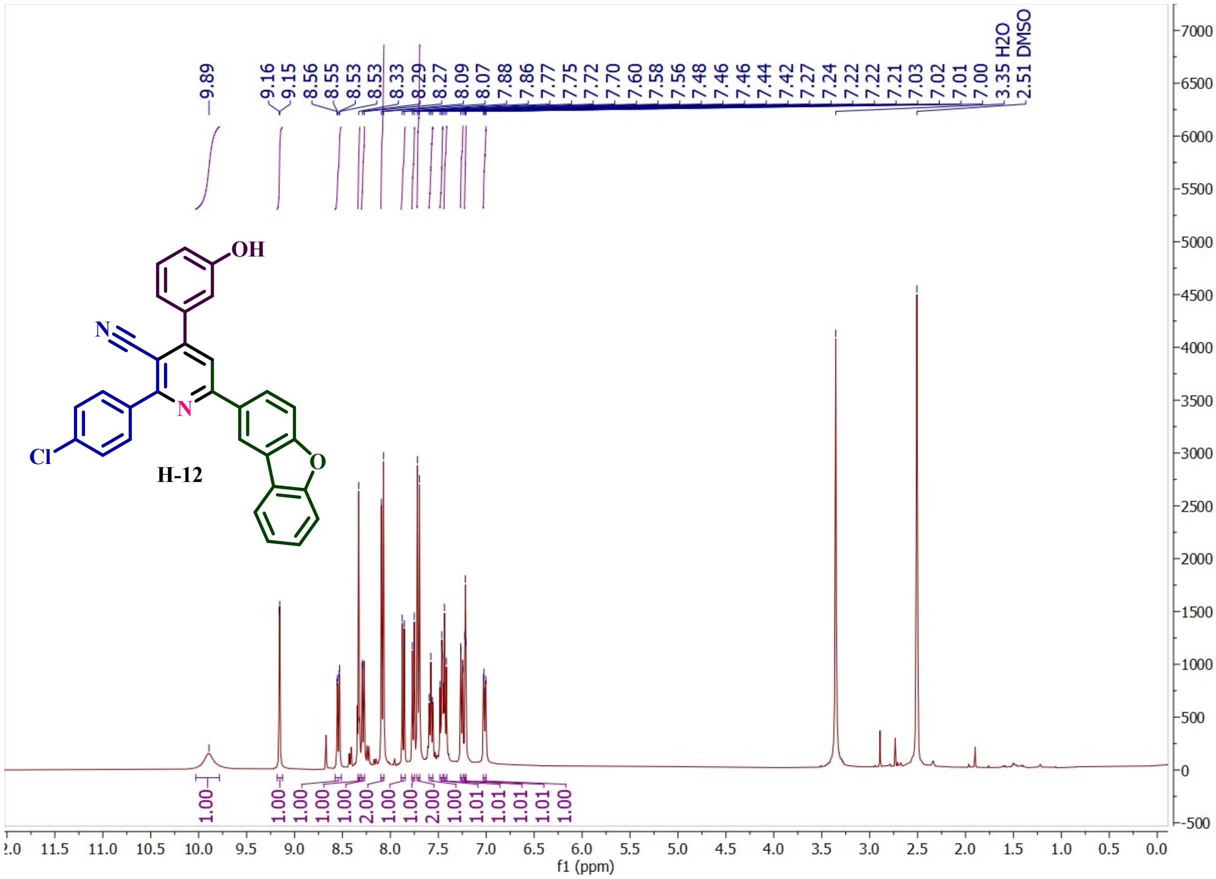

^{1}H-NMR spectrum of 2-(4-chlorophenyl)-6-(dibenzo[*b*,*d*]furan-2-yl)-4-(3-hydroxyphenyl)nicotinonitrile (H-12)

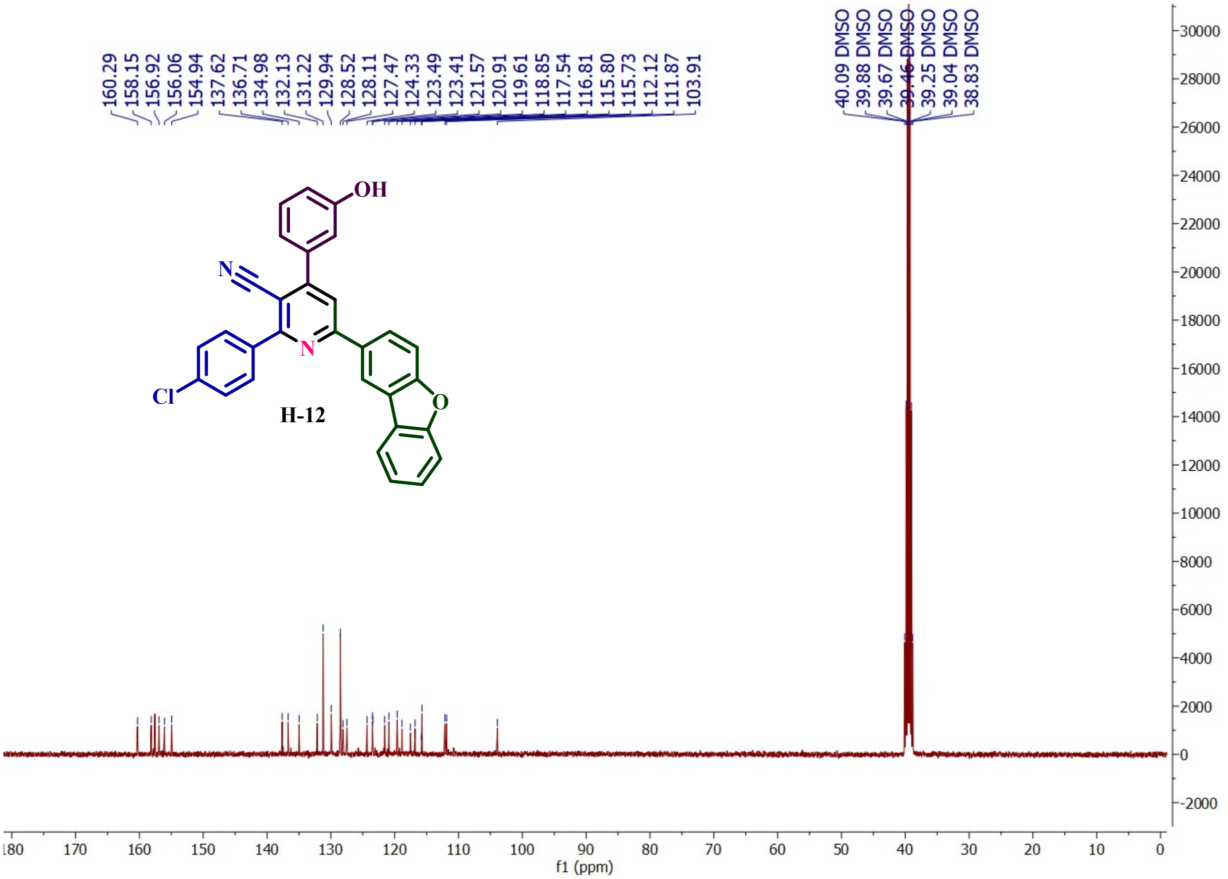

^{13}C-NMR spectrum of 2-(4-chlorophenyl)-6-(dibenzo[*b*,*d*]furan-2-yl)-4-(3-hydroxyphenyl)nicotinonitrile (H-12)

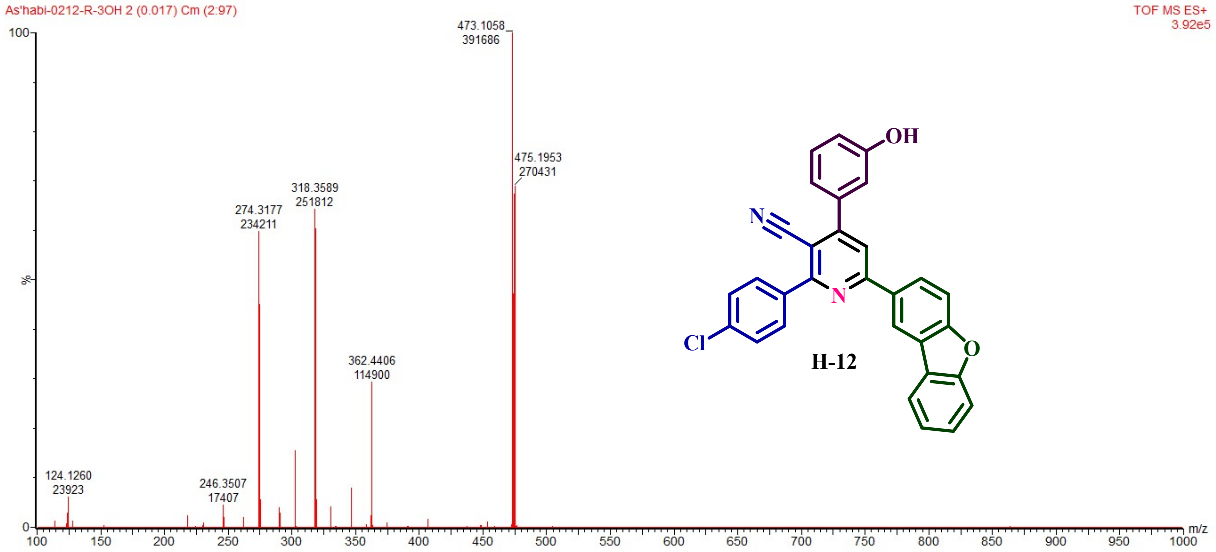

HR-Mass spectrum of 2-(4-chlorophenyl)-6-(dibenzo[*b*,*d*]furan-2-yl)-4-(3-hydroxyphenyl)nicotinonitrile (H-12)

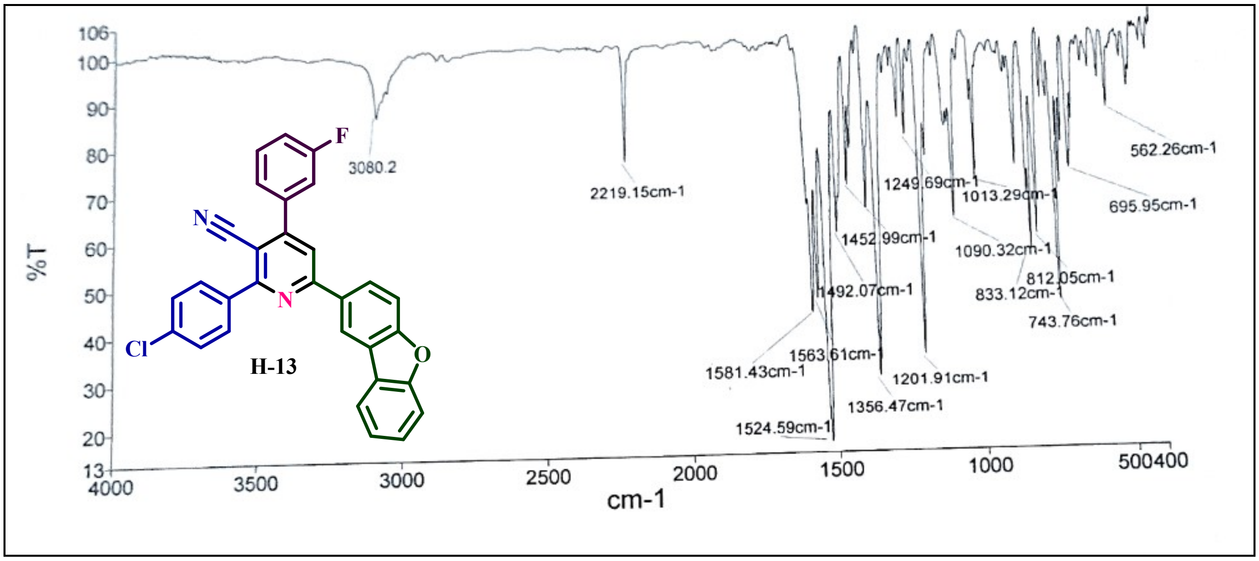

FT-IR spectrum of 2-(4-chlorophenyl)-6-(dibenzo[*b*,*d*]furan-2-yl)-4-(3-fluorophenyl)nicotinonitrile (H-13)

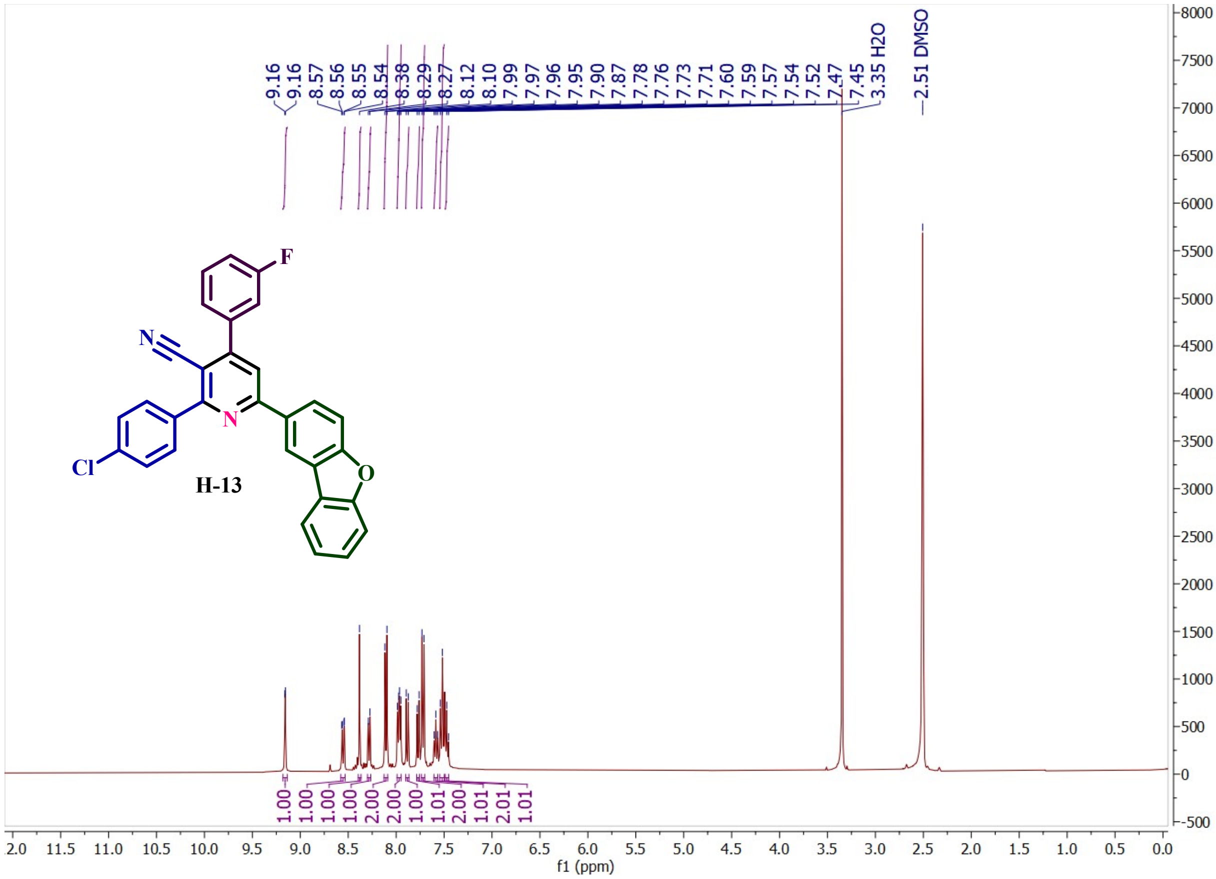

^{1}H-NMR spectrum of 2-(4-chlorophenyl)-6-(dibenzo[*b*,*d*]furan-2-yl)-4-(3-fluorophenyl)nicotinonitrile (H-13)

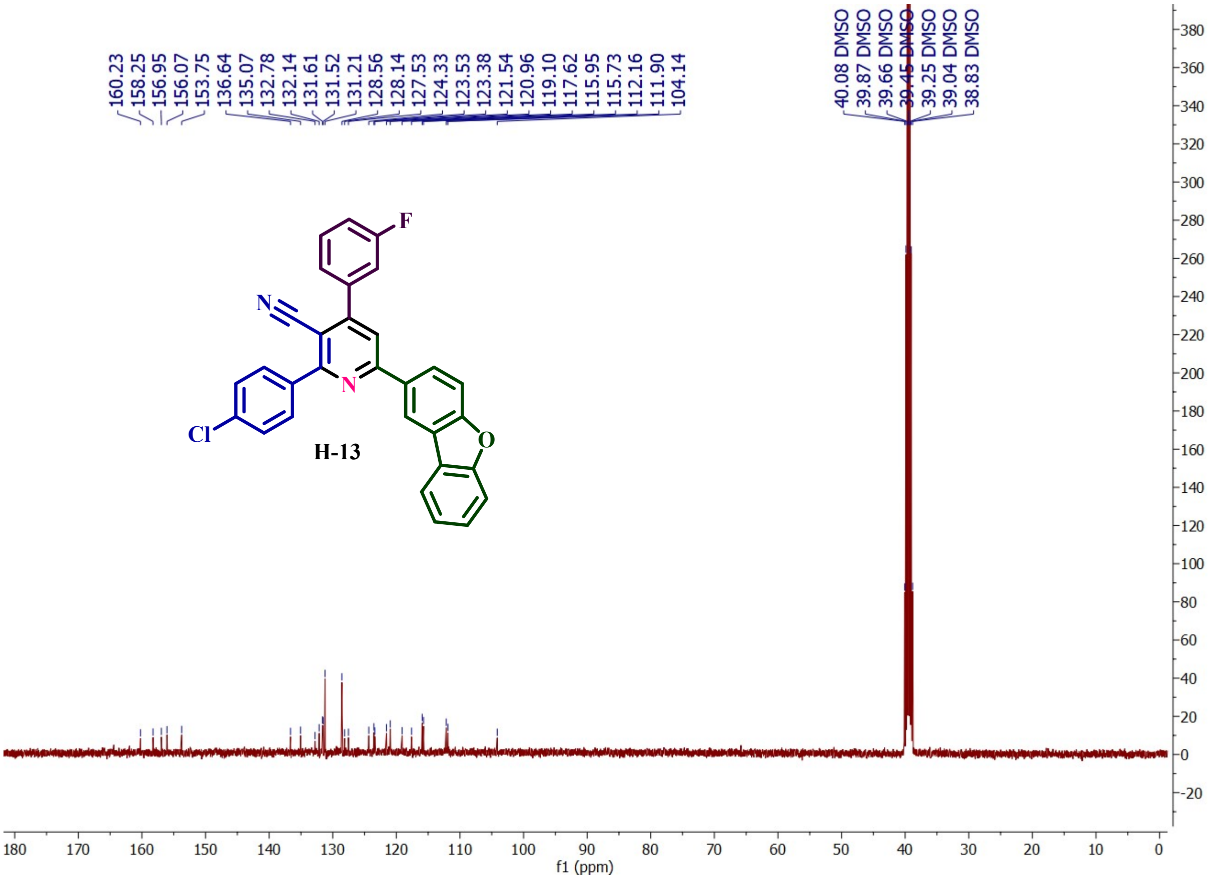

^{13}C-NMR spectrum of 2-(4-chlorophenyl)-6-(dibenzo[*b*,*d*]furan-2-yl)-4-(3-fluorophenyl)nicotinonitrile (H-13)

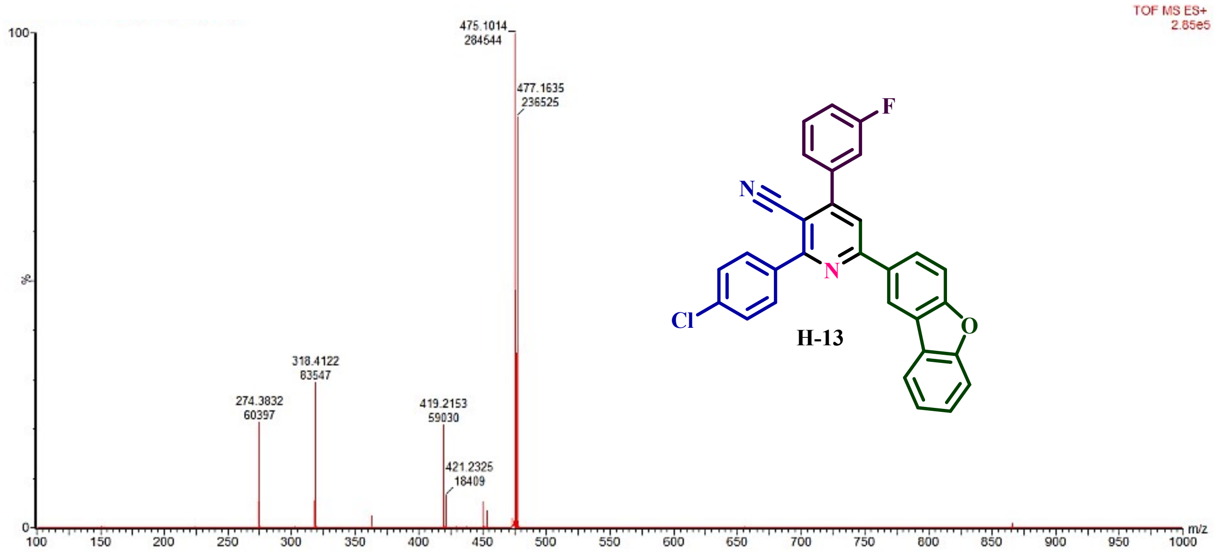

HR-Mass spectrum of 2-(4-chlorophenyl)-6-(dibenzo[*b*,*d*]furan-2-yl)-4-(3-fluorophenyl)nicotinonitrile (H-13)

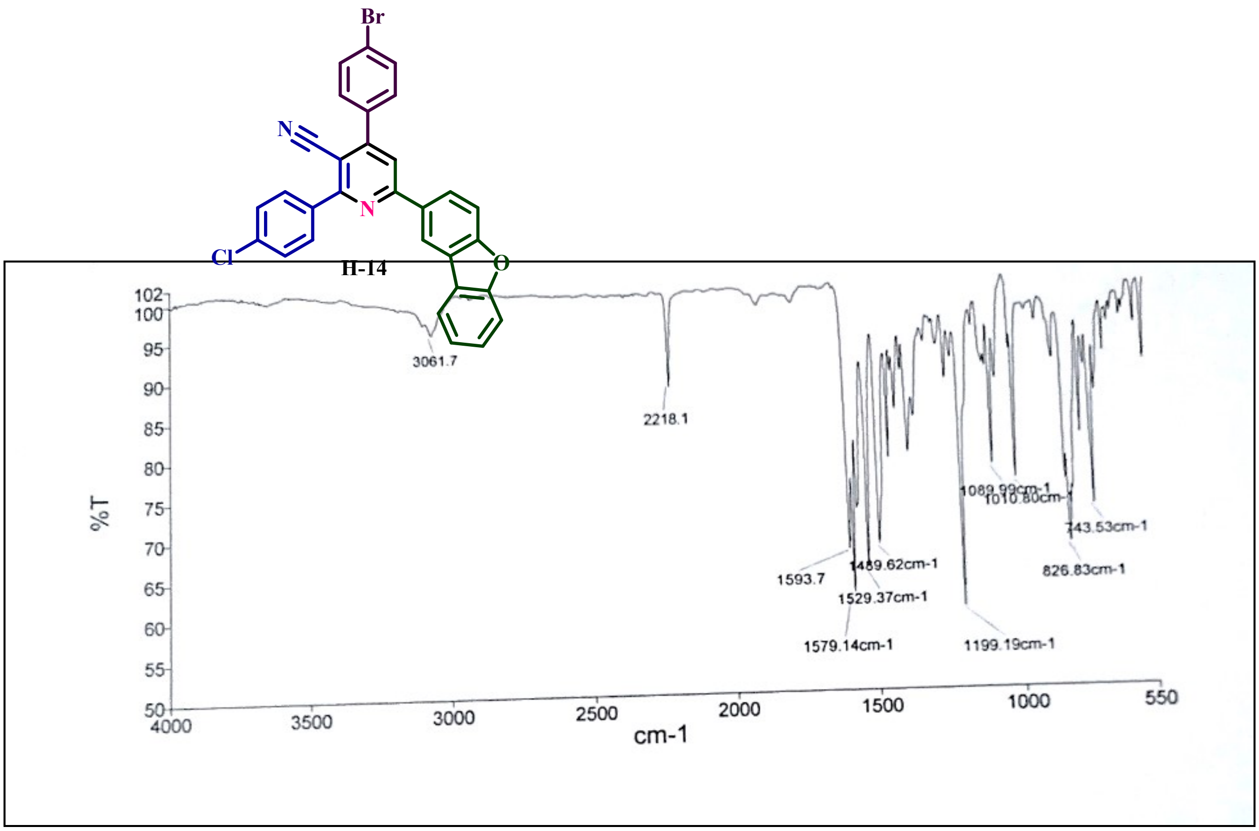

FT-IR spectrum of 4-(4-bromophenyl)-2-(4-chlorophenyl)-6-(dibenzo[*b*,*d*]furan-2-yl)nicotinonitrile (H-14)

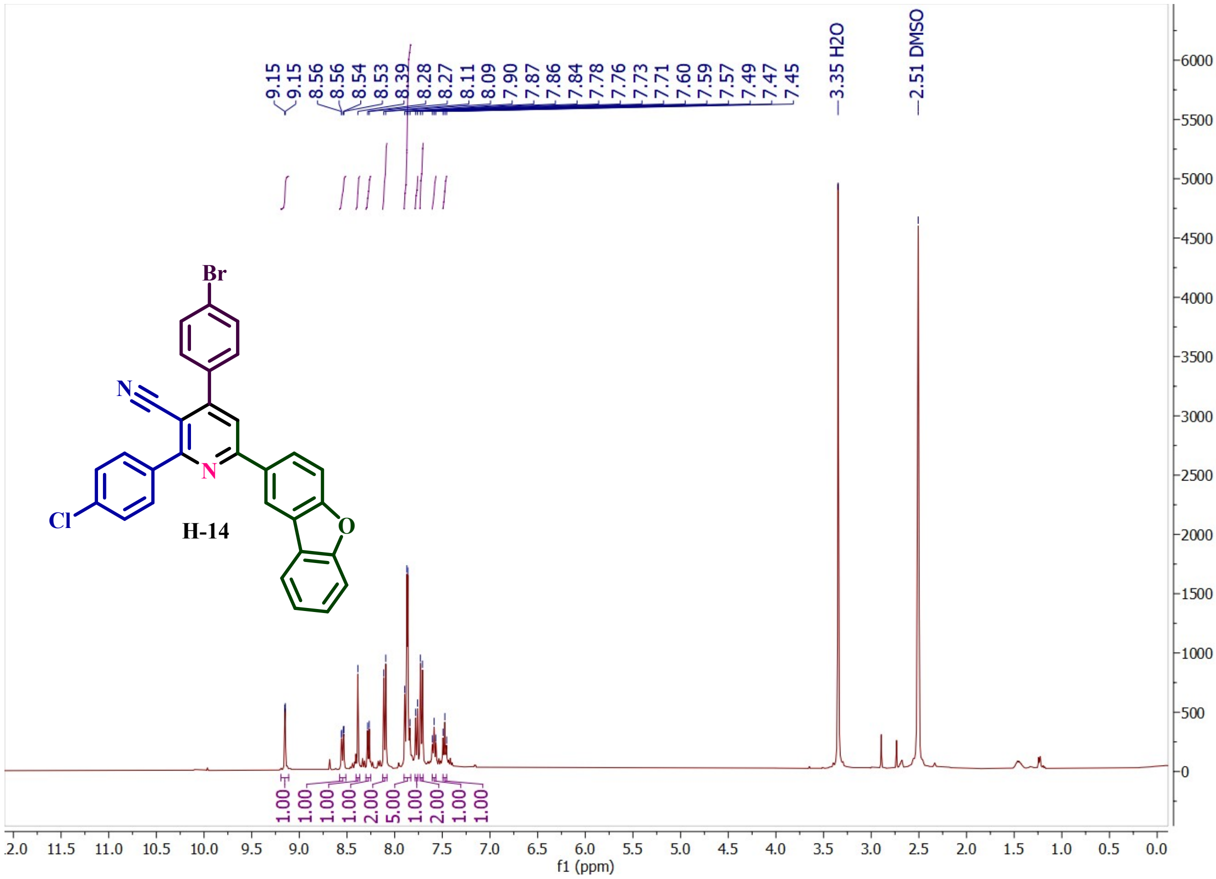

^{1}H-NMR spectrum of 4-(4-bromophenyl)-2-(4-chlorophenyl)-6-(dibenzo[*b*,*d*]furan-2-yl)nicotinonitrile (H-14)

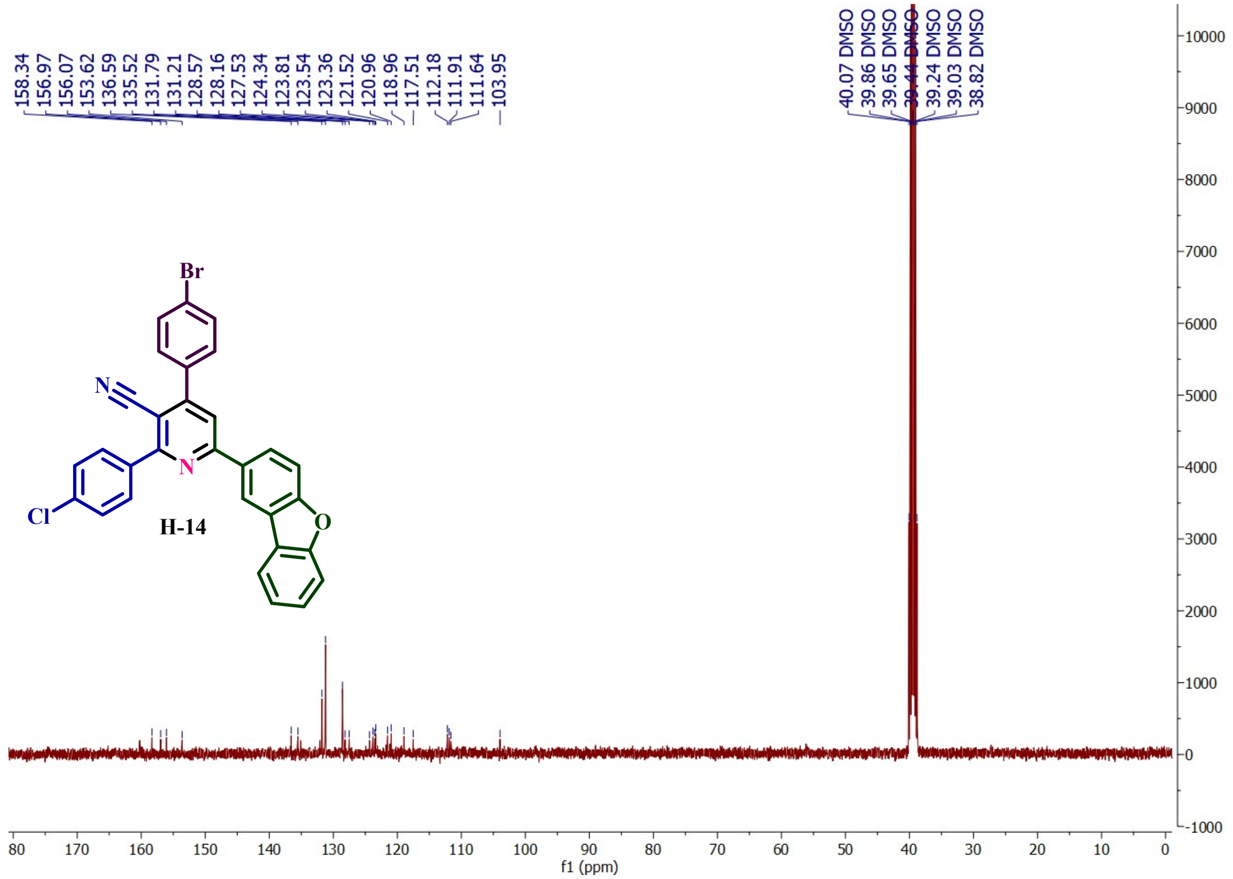

^{13}C-NMR spectrum of 4-(4-bromophenyl)-2-(4-chlorophenyl)-6-(dibenzo[*b*,*d*]furan-2-yl)nicotinonitrile (H-14)

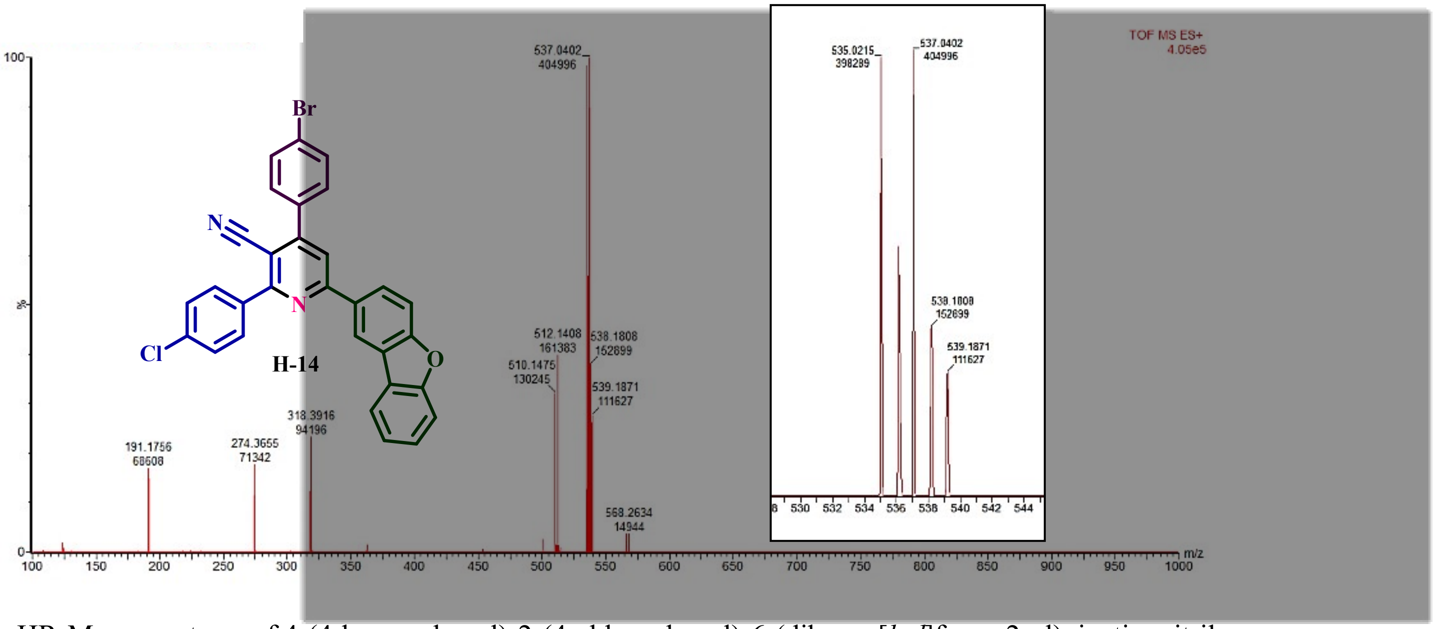

HR-Mass spectrum of 4-(4-bromophenyl)-2-(4-chlorophenyl)-6-(dibenzo[*b*,*d*]furan-2-yl)nicotinonitrile (H-14)

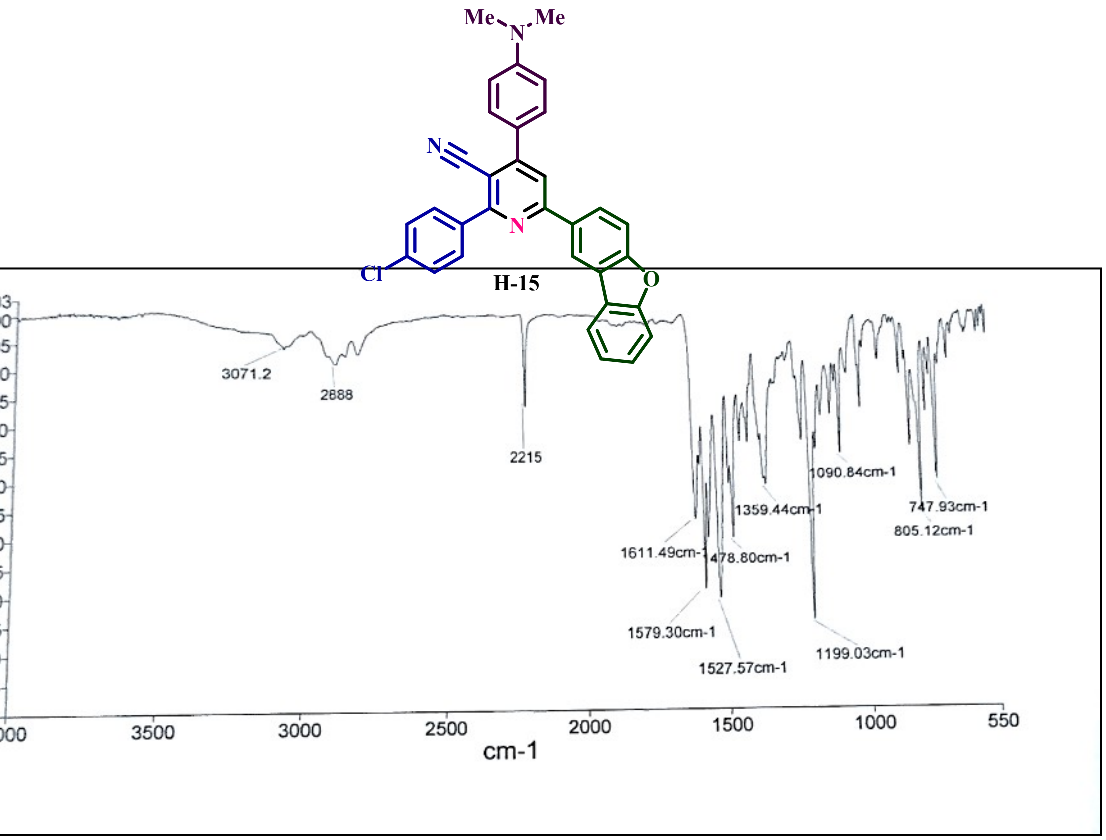

FT-IR spectrum of 2-(4-chlorophenyl)-6-(dibenzo[*b*,*d*]furan-2-yl)-4-(4-(dimethylamino)phenyl)nicotinonitrile (H-15)

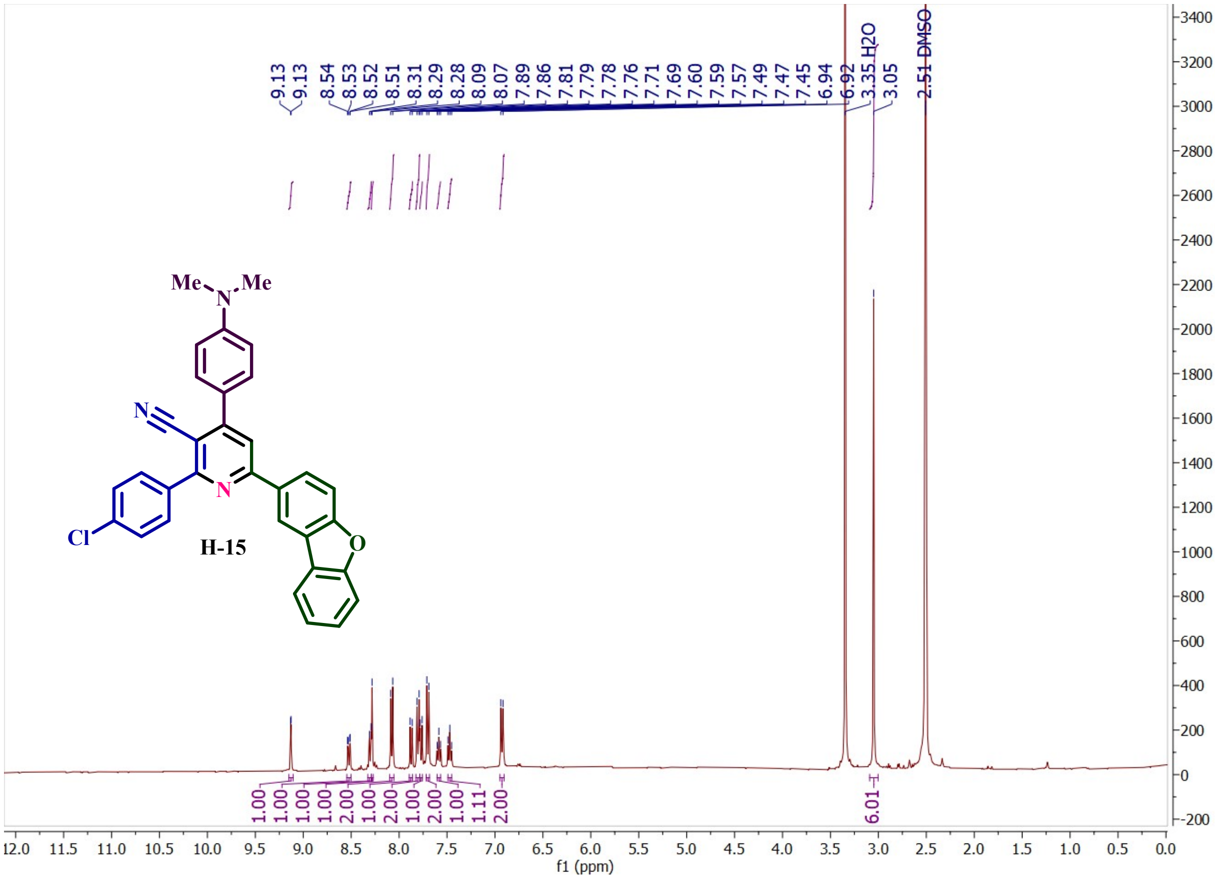

^{1}H-NMR spectrum of 2-(4-chlorophenyl)-6-(dibenzo[*b*,*d*]furan-2-yl)-4-(4-(dimethylamino)phenyl)nicotinonitrile (H-15)

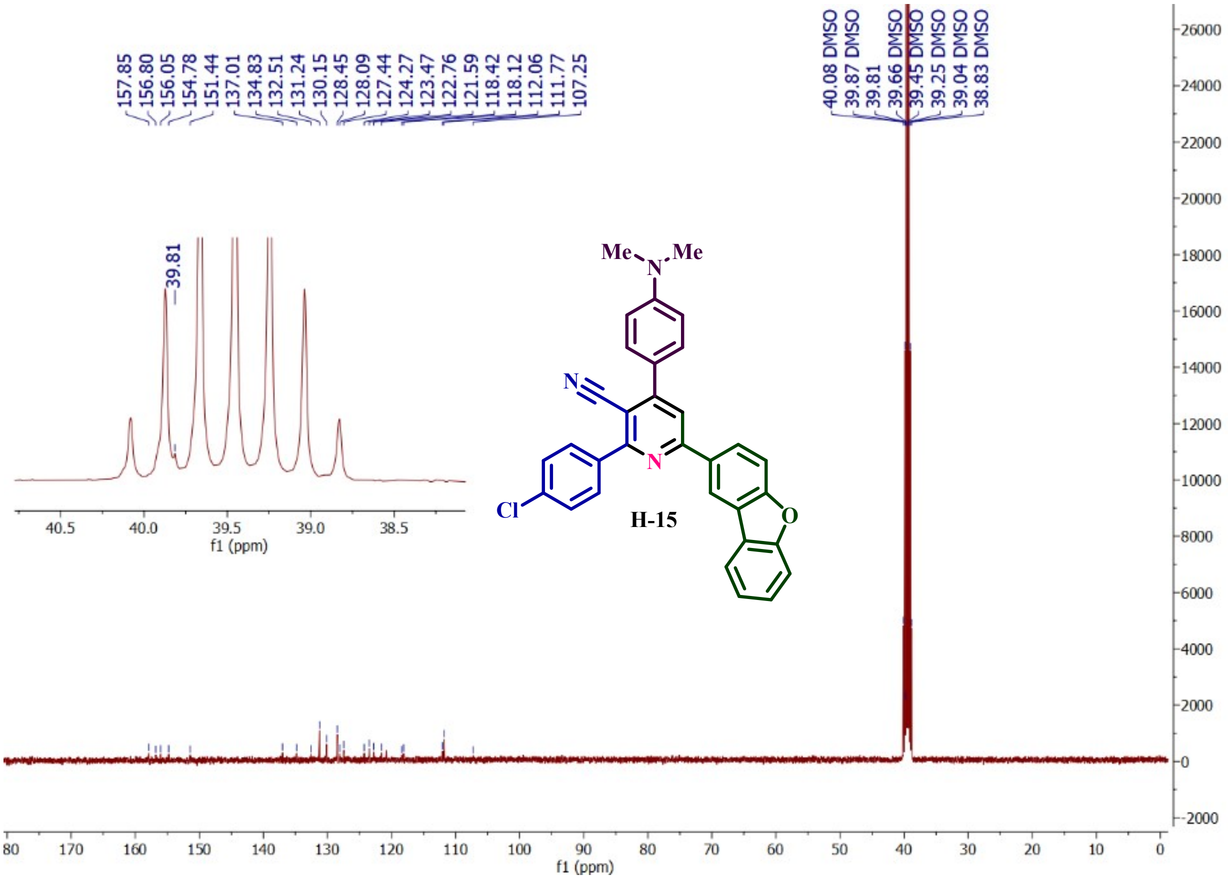

^{13}C-NMR spectrum of 2-(4-chlorophenyl)-6-(dibenzo[*b*,*d*]furan-2-yl)-4-(4-(dimethylamino)phenyl)nicotinonitrile (H-15)

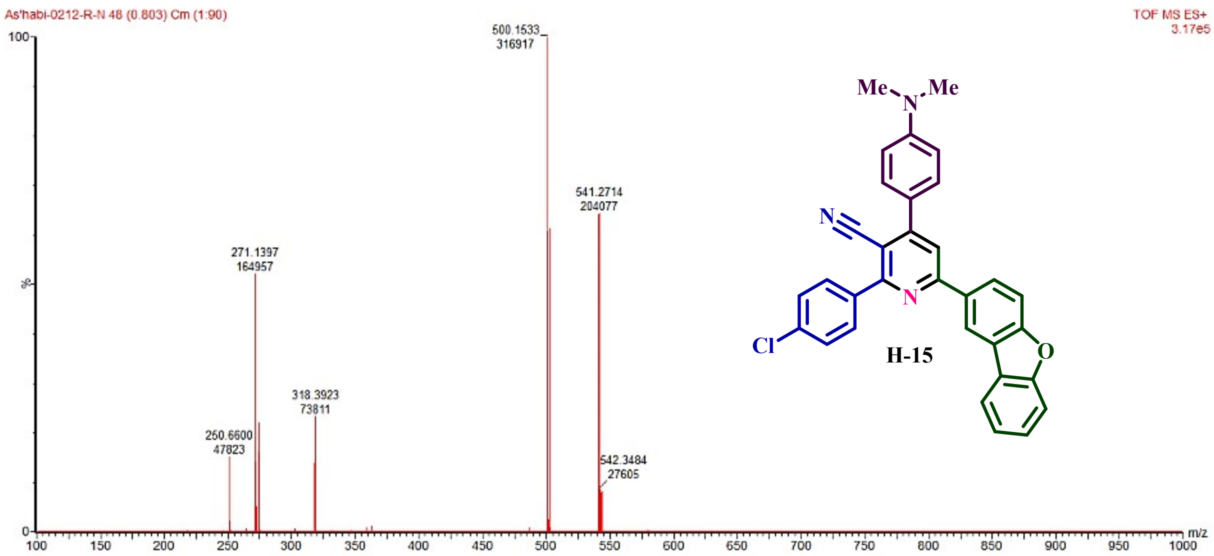

HR-Mass spectrum of 2-(4-chlorophenyl)-6-(dibenzo[*b*,*d*]furan-2-yl)-4-(4-(dimethylamino)phenyl)nicotinonitrile (H-15)

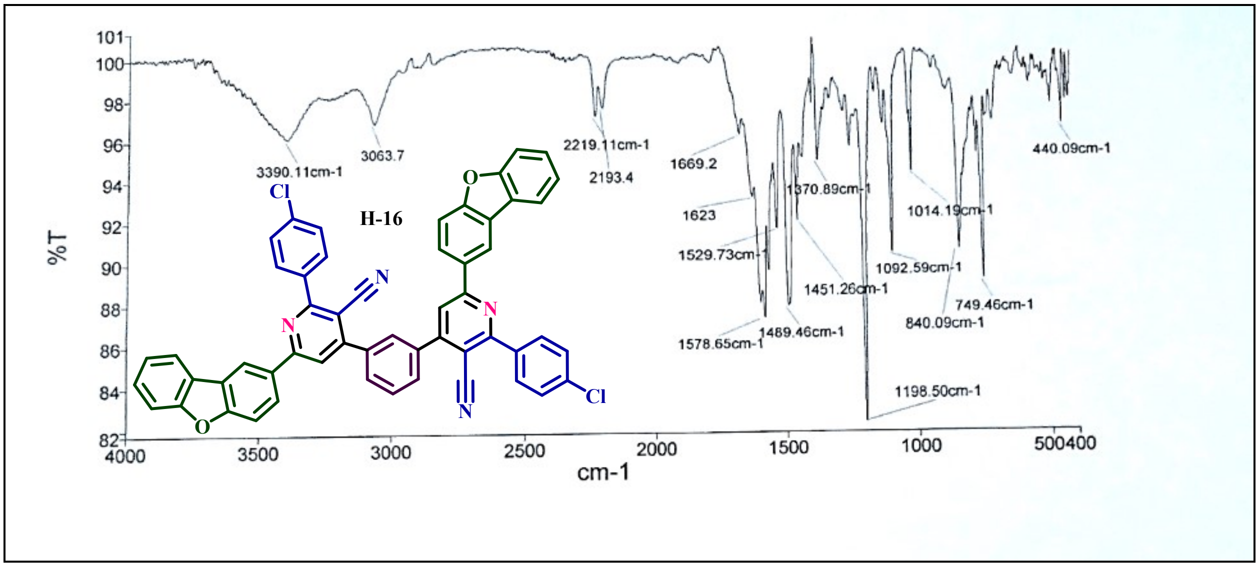

FT-IR spectrum of 4,4'-(1,3-phenylene)bis(2-(4-chlorophenyl)-6-(dibenzo[*b*,*d*]furan-2-yl)nicotinonitrile) (H-16)

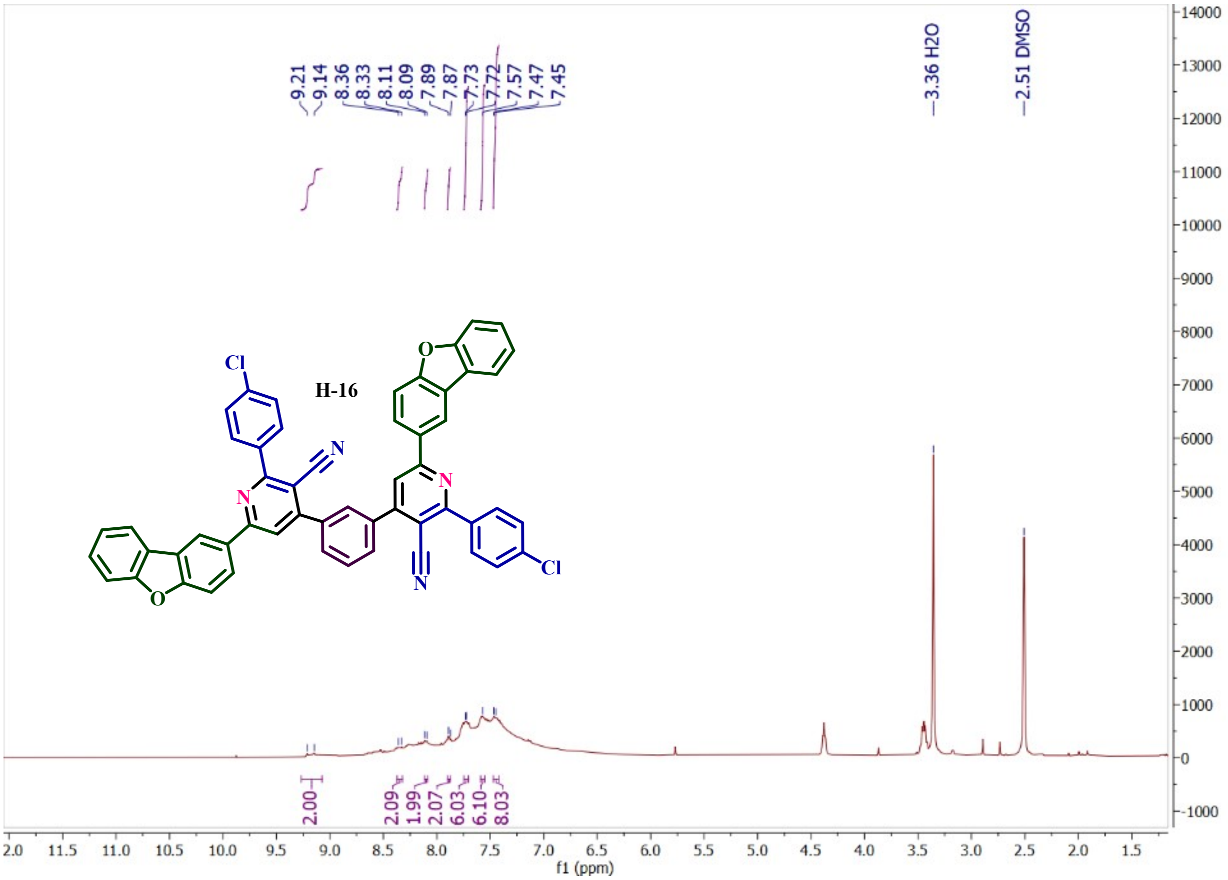

^{1}H-NMR spectrum of 4,4'-(1,3-phenylene)bis(2-(4-chlorophenyl)-6-(dibenzo[*b*,*d*]furan-2-yl)nicotinonitrile) (H-16)

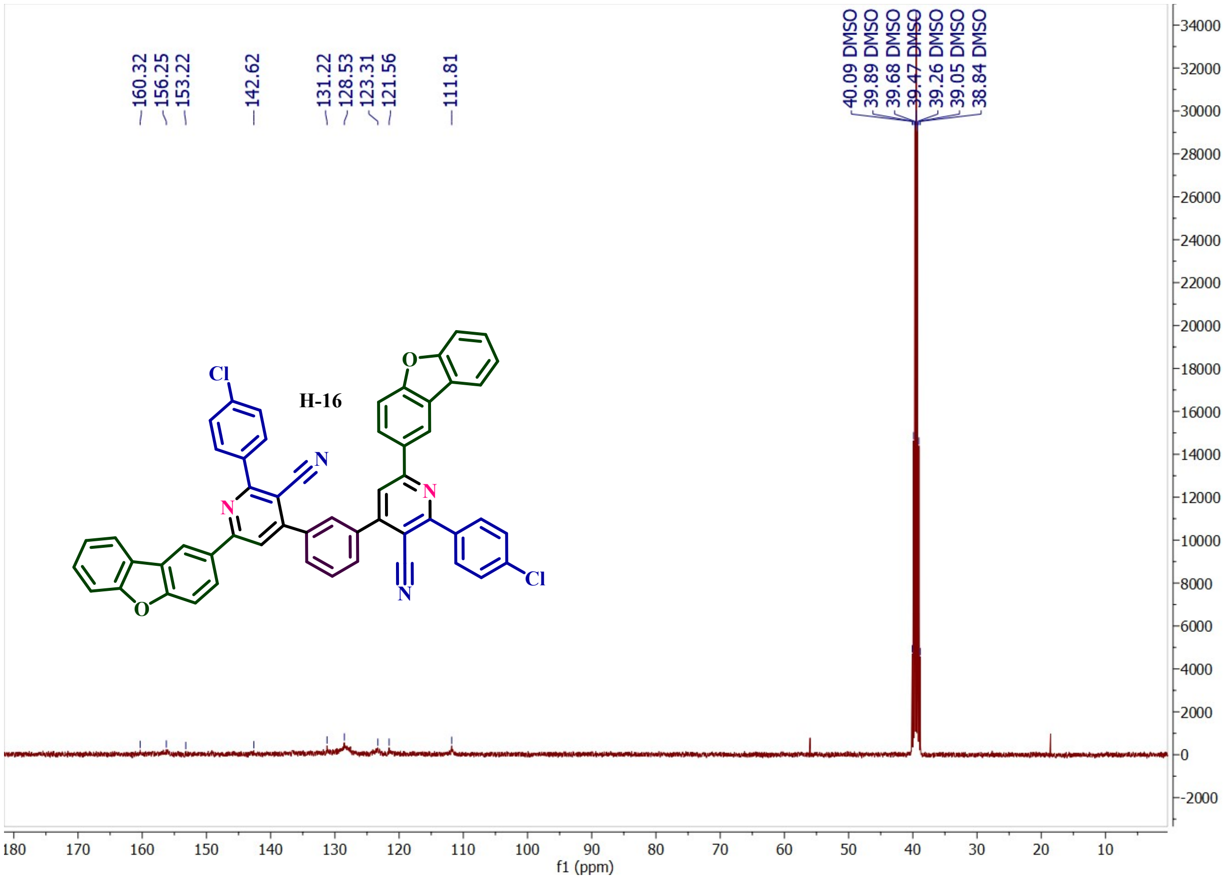

^{13}C-NMR spectrum of 4,4'-(1,3-phenylene)bis(2-(4-chlorophenyl)-6-(dibenzo[*b*,*d*]furan-2-yl)nicotinonitrile) (H-16)

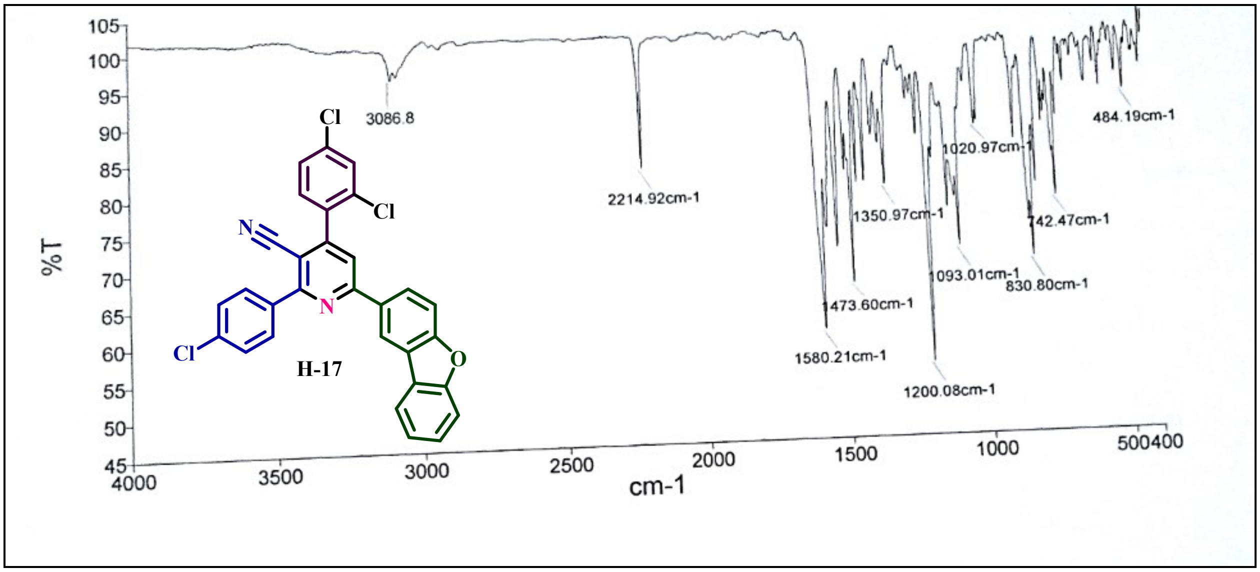

FT-IR spectrum of 2-(4-chlorophenyl)-6-(dibenzo[*b*,*d*]furan-2-yl)-4-(2,4-dichlorophenyl)nicotinonitrile (H-17)

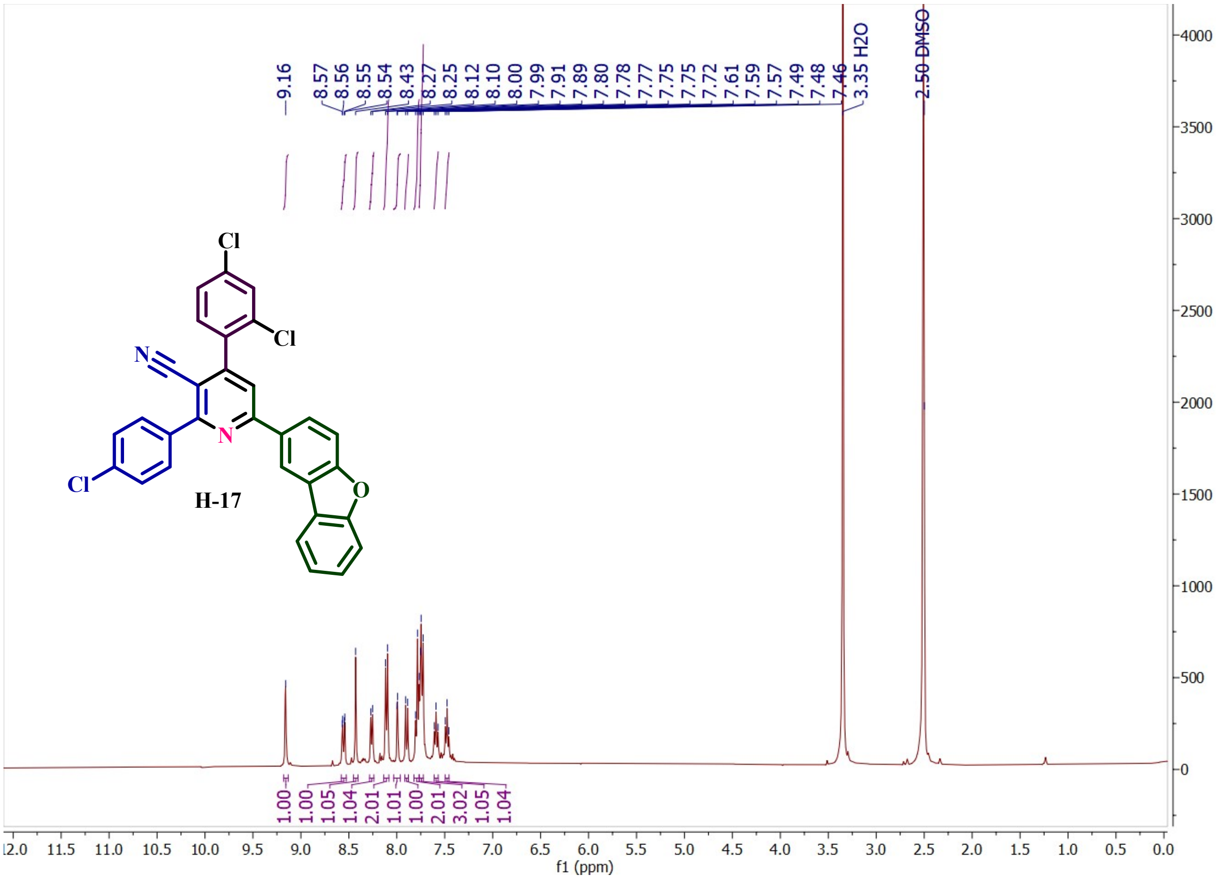

^{1}H-NMR spectrum of 2-(4-chlorophenyl)-6-(dibenzo[*b*,*d*]furan-2-yl)-4-(2,4-dichlorophenyl)nicotinonitrile (H-17)

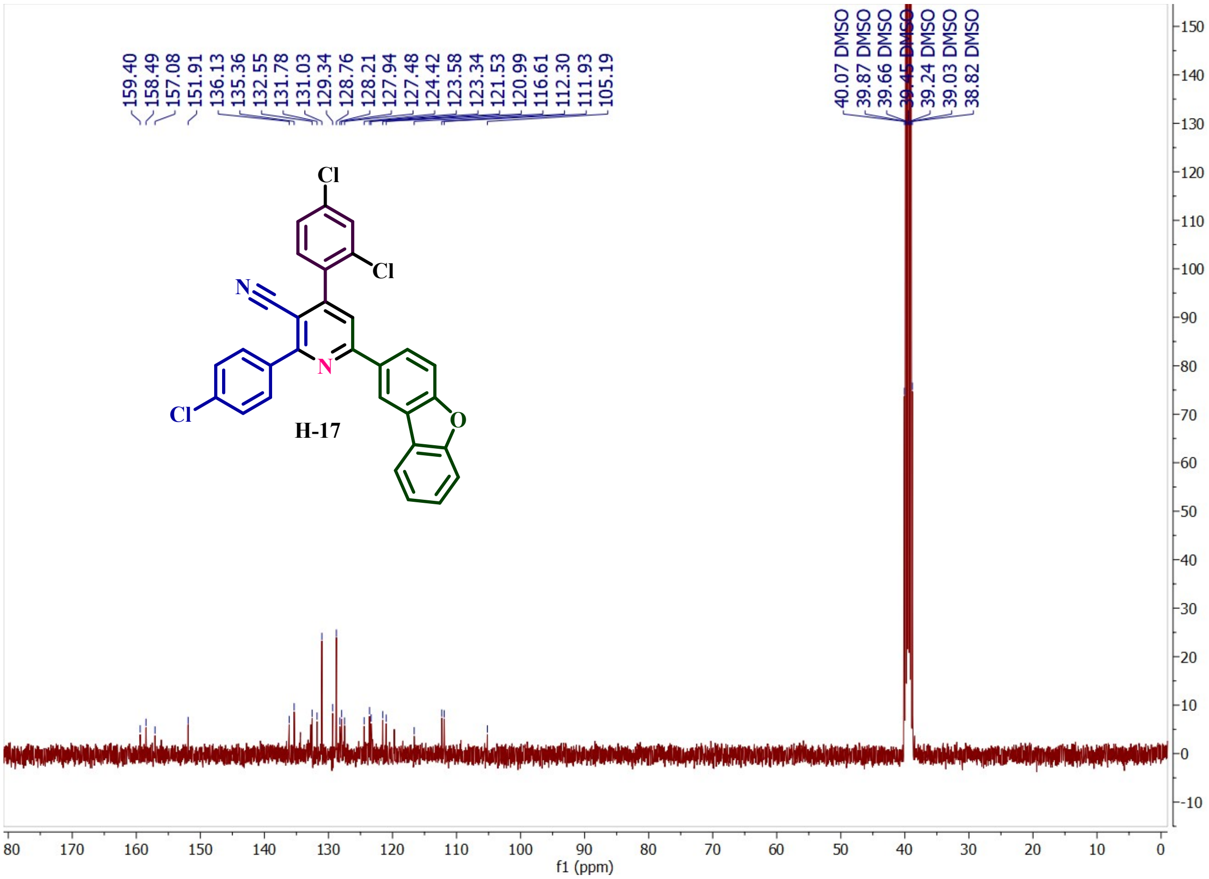

^{13}C-NMR spectrum of 2-(4-chlorophenyl)-6-(dibenzo[*b*,*d*]furan-2-yl)-4-(2,4-dichlorophenyl)nicotinonitrile (H-17)

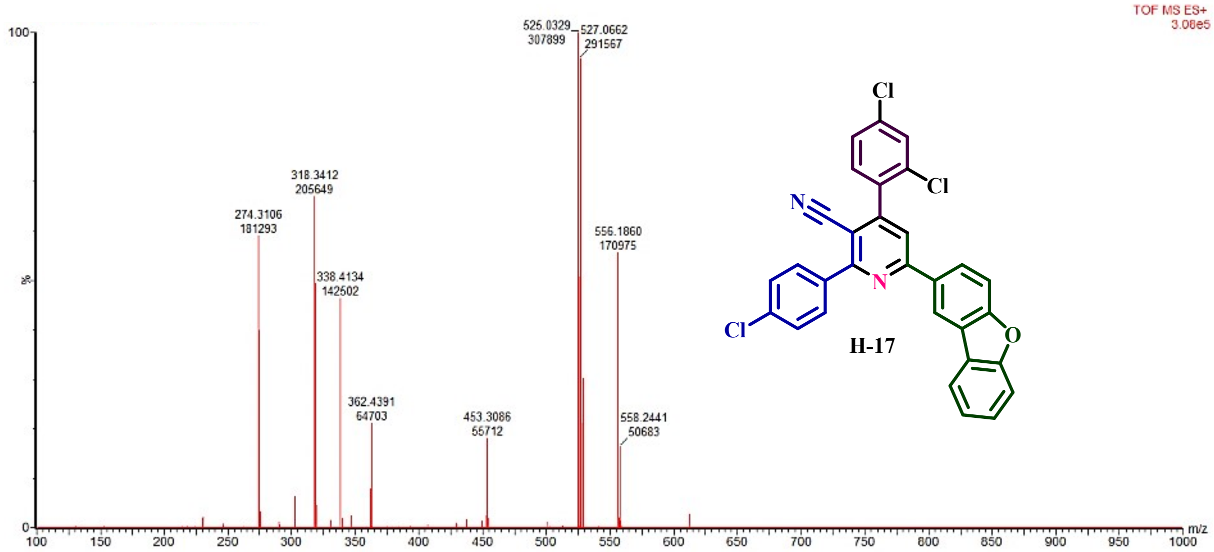

HR-Mass spectrum of 2-(4-chlorophenyl)-6-(dibenzo[*b*,*d*]furan-2-yl)-4-(2,4-dichlorophenyl)nicotinonitrile (H-17)

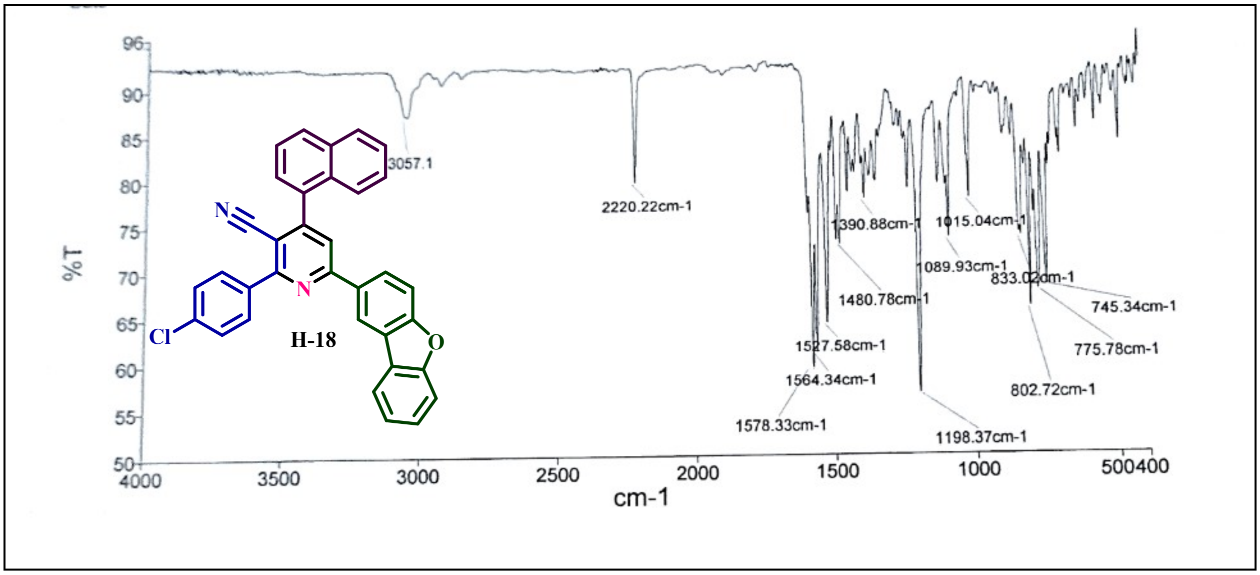

FT-IR spectrum of 2-(4-chlorophenyl)-6-(dibenzo[*b*,*d*]furan-2-yl)-4-(naphthalen-1-yl)nicotinonitrile (H-18)

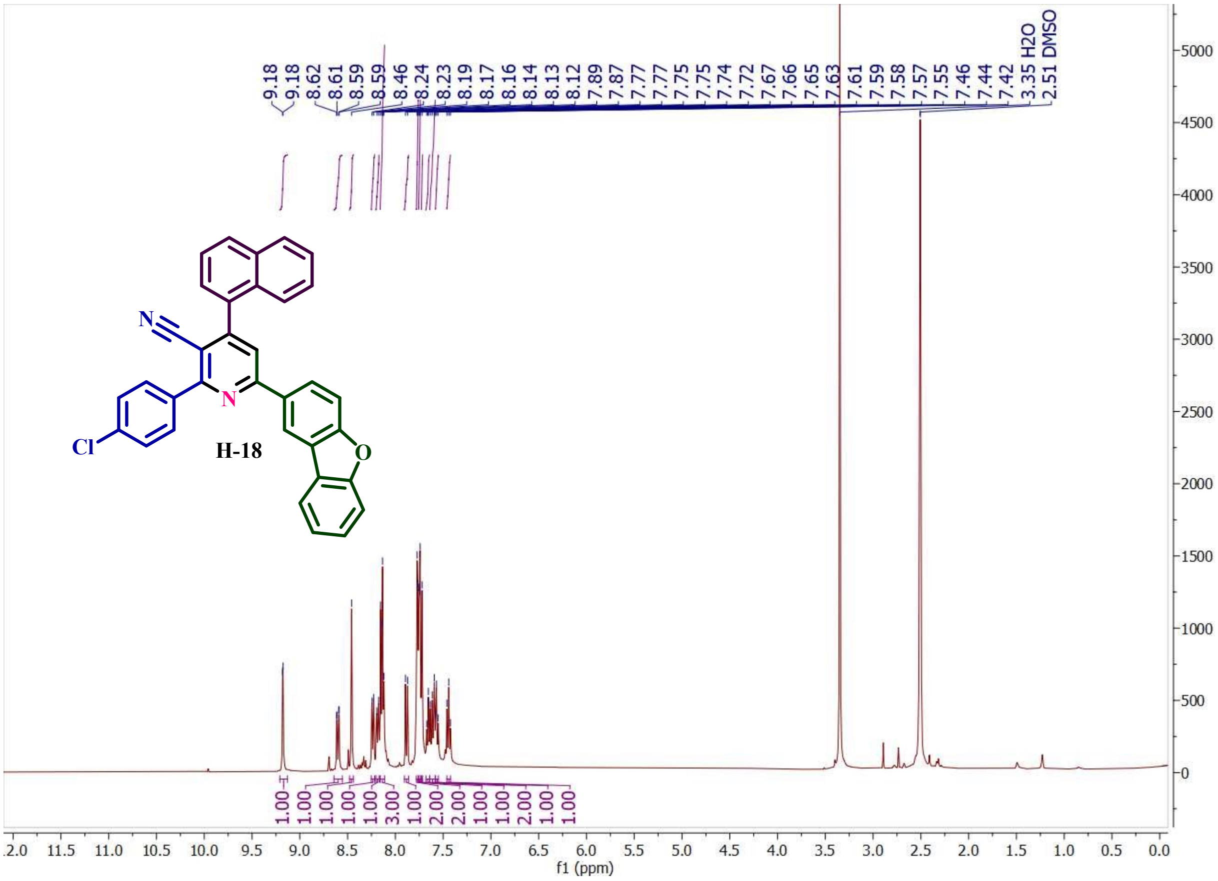

^{1}H-NMR spectrum of 2-(4-chlorophenyl)-6-(dibenzo[*b*,*d*]furan-2-yl)-4-(naphthalen-1-yl)nicotinonitrile (H-18)

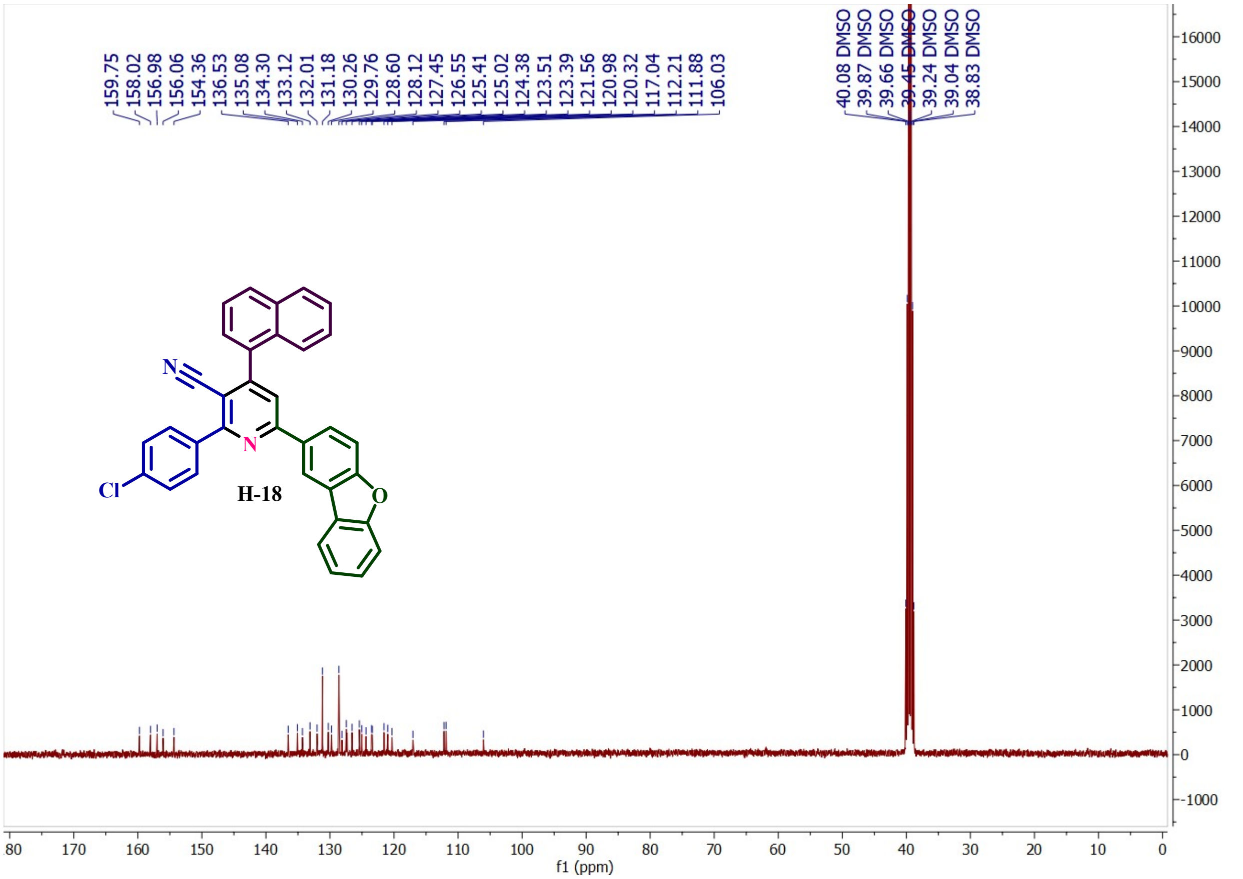

^{13}C-NMR spectrum of 2-(4-chlorophenyl)-6-(dibenzo[*b*,*d*]furan-2-yl)-4-(naphthalen-1-yl)nicotinonitrile (H-18)

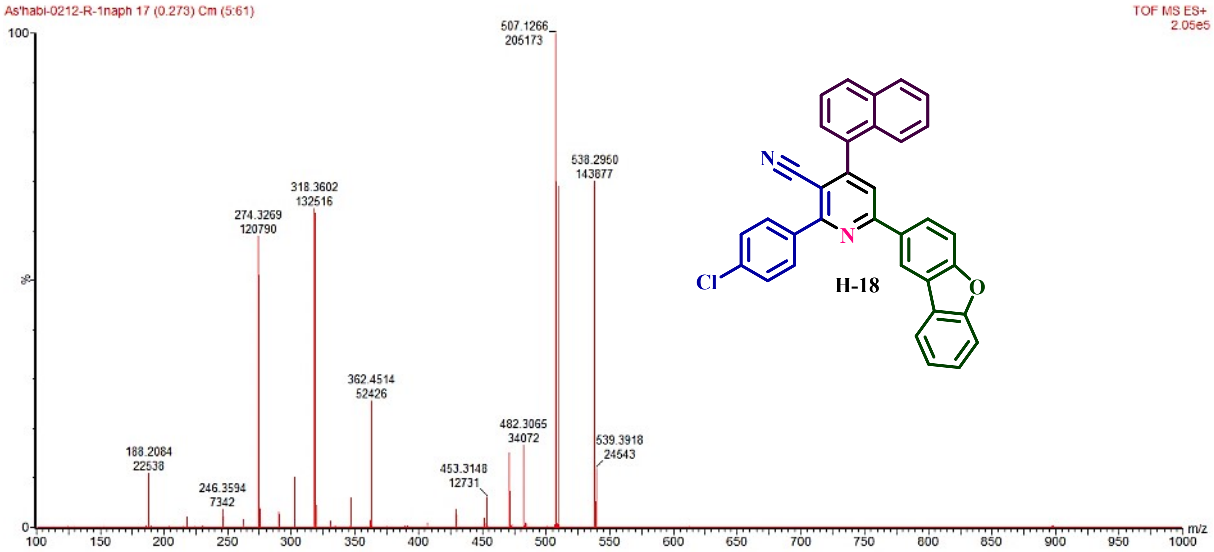

HR-Mass spectrum of 2-(4-chlorophenyl)-6-(dibenzo[*b*,*d*]furan-2-yl)-4-(naphthalen-1-yl)nicotinonitrile (H-18)

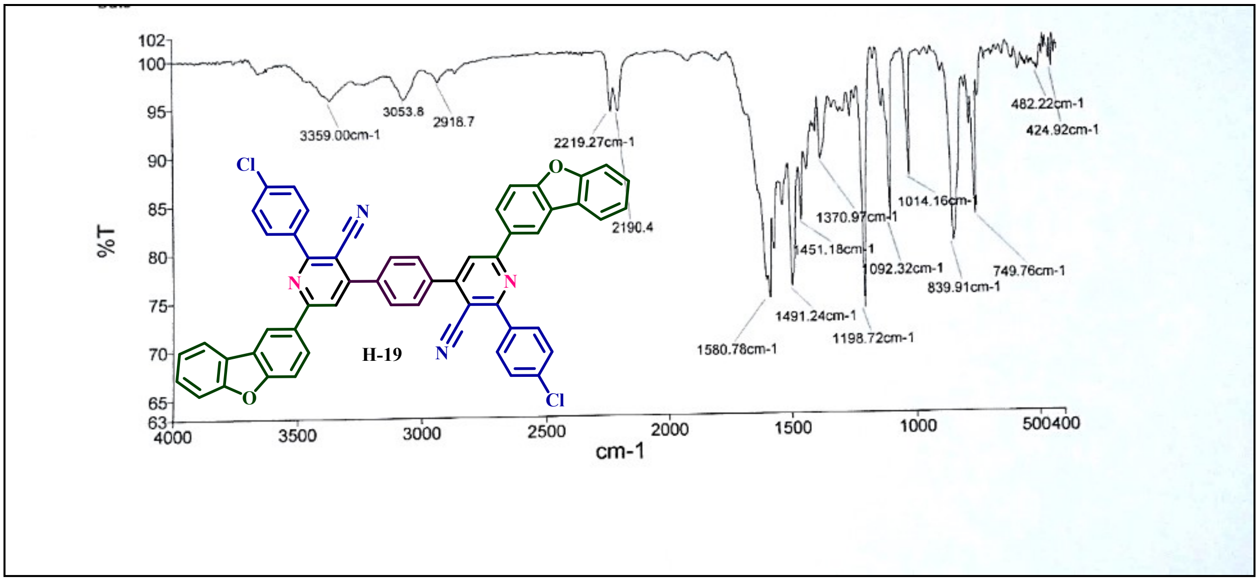

FT-IR spectrum of 4,4'-(1,4-phenylene)bis(2-(4-chlorophenyl)-6-(dibenzo[*b*,*d*]furan-2-yl)nicotinonitrile) (H-19)

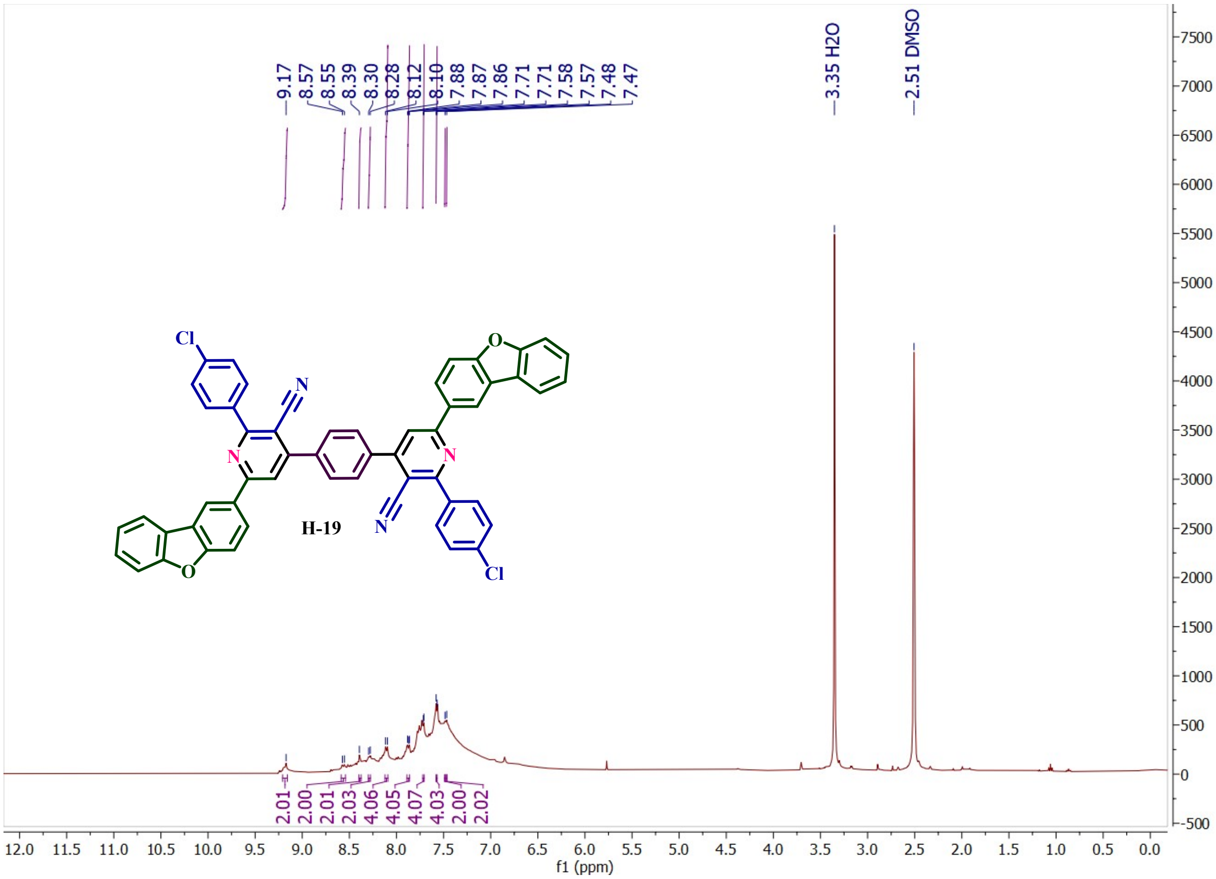

^{1}H-NMR spectrum of 4,4'-(1,4-phenylene)bis(2-(4-chlorophenyl)-6-(dibenzo[*b*,*d*]furan-2-yl)nicotinonitrile) (H-19)

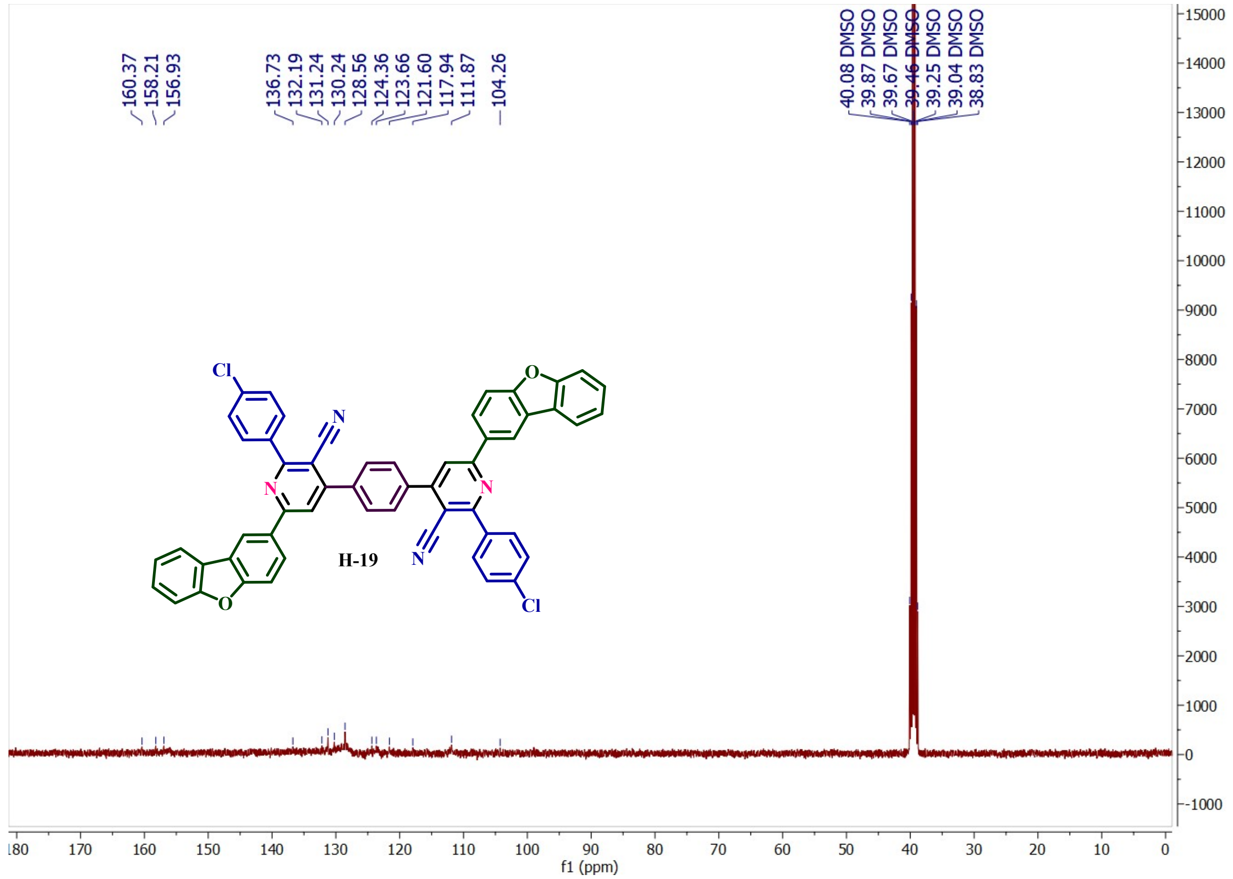

^{13}C-NMR spectrum of 4,4'-(1,4-phenylene)bis(2-(4-chlorophenyl)-6-(dibenzo[*b*,*d*]furan-2-yl)nicotinonitrile) (H-19)

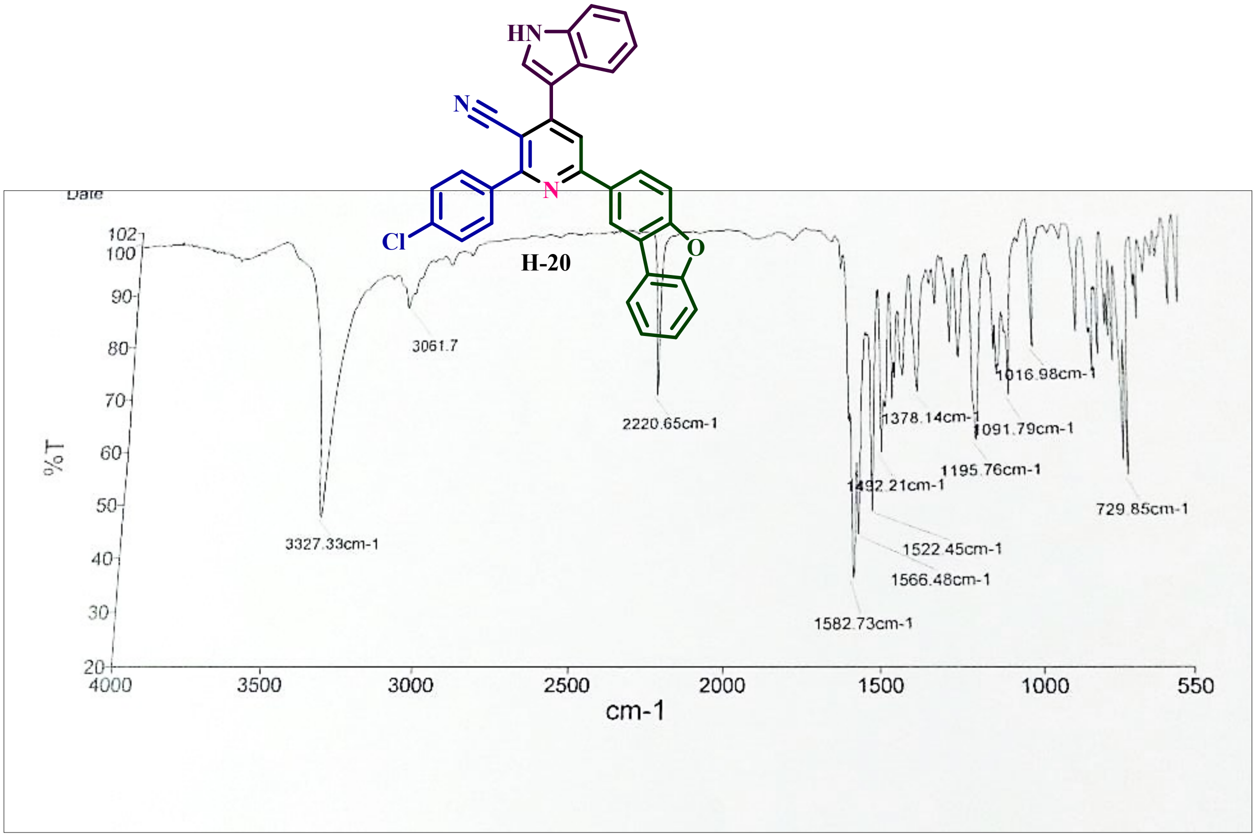

FT-IR spectrum of 2-(4-chlorophenyl)-6-(dibenzo[*b*,*d*]furan-2-yl)-4-(1*H*-indol-3-yl)nicotinonitrile (H-20)

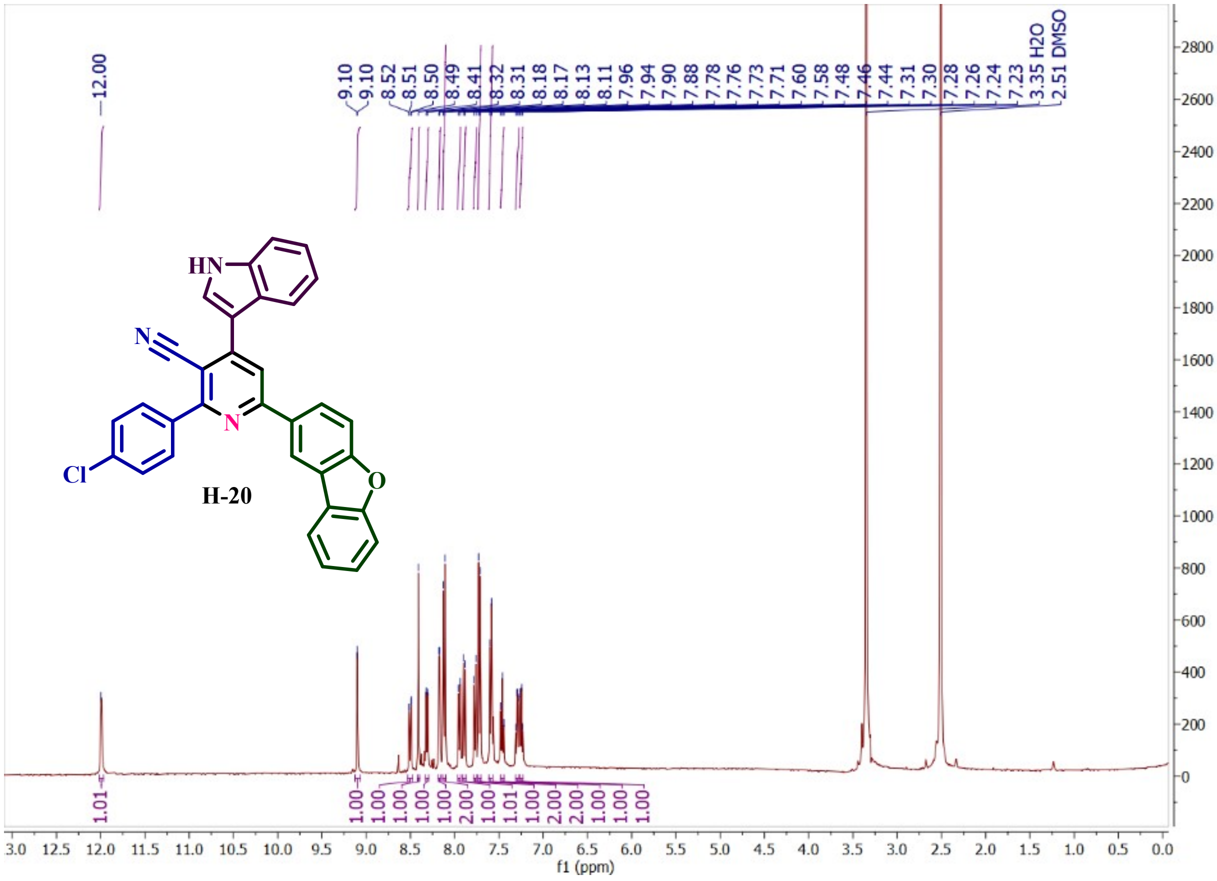

^{1}H-NMR spectrum of 2-(4-chlorophenyl)-6-(dibenzo[*b*,*d*]furan-2-yl)-4-(1*H*-indol-3-yl)nicotinonitrile (H-20)

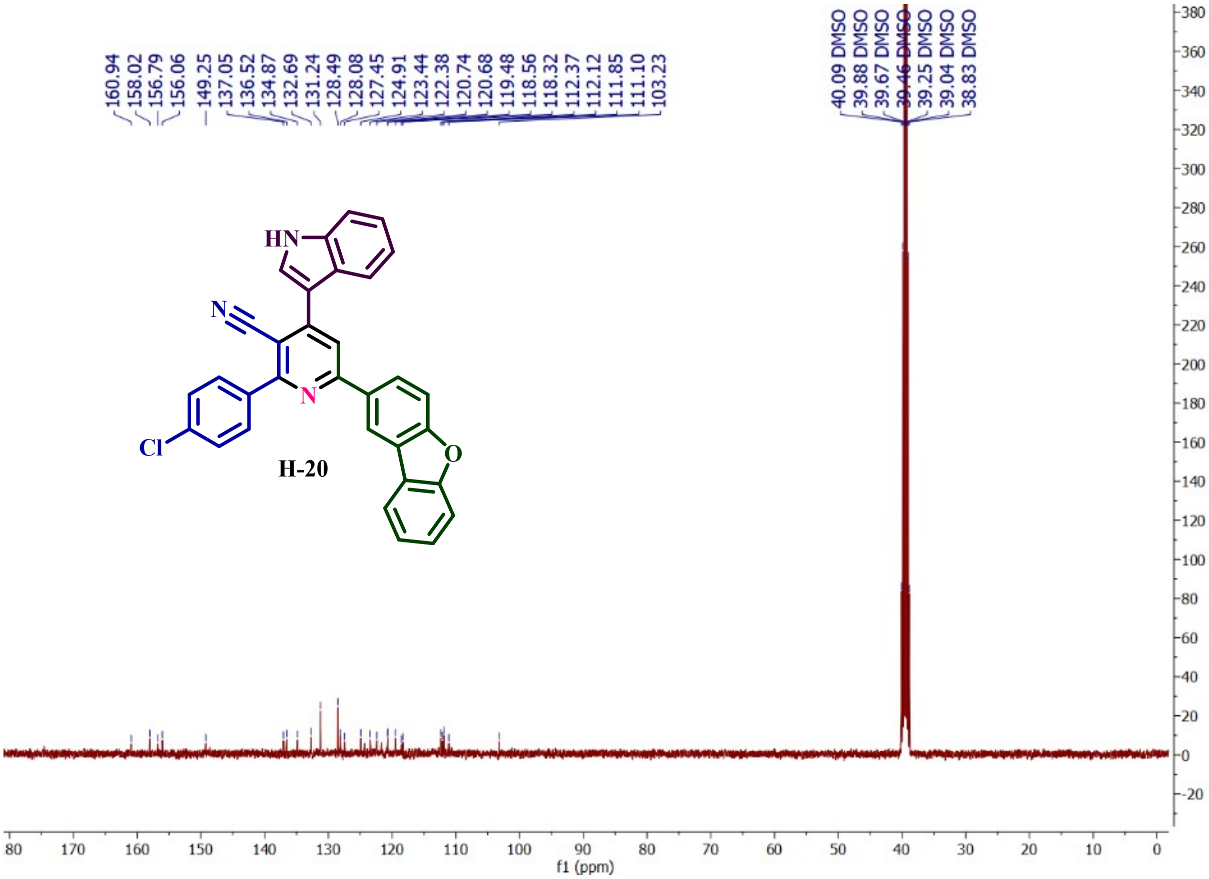

^{13}C-NMR spectrum of 2-(4-chlorophenyl)-6-(dibenzo[*b*,*d*]furan-2-yl)-4-(1*H*-indol-3-yl)nicotinonitrile (H-20)

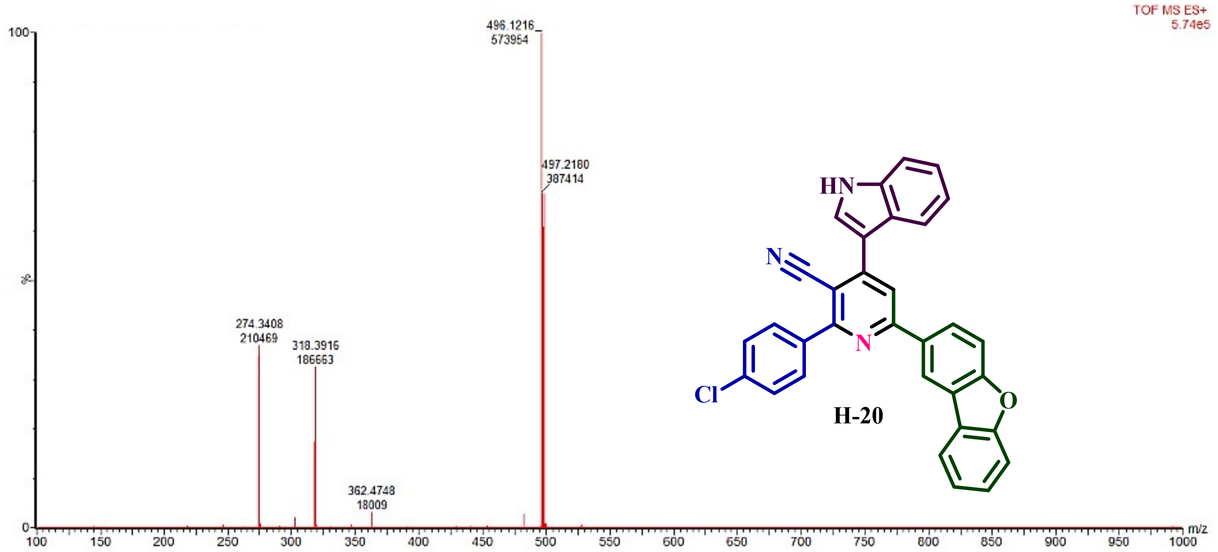

HR-Mass spectrum of 2-(4-chlorophenyl)-6-(dibenzo[*b*,*d*]furan-2-yl)-4-(1*H*-indol-3-yl)nicotinonitrile (H-20)
